# When carbonyls beat aromaticity: an additive framework for predicting tautomeric preferences

**DOI:** 10.1039/d6sc03354g

**Published:** 2026-10-13

**Authors:** Lucas Araujo, Felipe Fantuzzi, Pierre M. Esteves, Thiago M. Cardozo

**Affiliations:** a Instituto de Química, Universidade Federal do Rio de Janeiro Av. Athos da Silveira Ramos, 149, CT, A-622, Cid. Univ. Rio de Janeiro RJ 21941-909 Brazil thiago@iq.ufrj.br pesteves@iq.ufrj.br; b Chemistry and Forensic Science, School of Natural Sciences, University of Kent Park Wood Rd Canterbury CT2 7NH UK f.fantuzzi@kent.ac.uk

## Abstract

Aromatic structures are often regarded as privileged stabilising motifs, generally retained even when competing structural effects are present. Here, we show that functional-group energetics can overturn this expectation in heteroaromatic tautomerism. Analysis of the 2-hydroxypyridine/2-pyridone equilibrium reveals a near-cancellation between carbonyl stabilisation and the energetic penalty associated with perturbing the aromatic ring framework, accounting for the small experimental energy gap. Extending this approach to pyridines, diazines, and triazines reveals a simple additive pattern: hydroxy/carbonyl exchange favours carbonylated, non-aromatic tautomers, typically requiring two C=O/N–H units to invert the preference, whereas amino/imine and methyl/methylidene exchanges reinforce aromatic tautomers. Spin-coupled generalised valence bond (SCGVB) calculations analysed within the GPF-EP method show that the quasi-classical component favours the aromatic 6c–6e π framework, whereas quantum interference favours localised 2c–2e bonds, especially C=O. This provides an energy-decomposition expression of π-distortivity: without interference, the aromatic tautomers would predominate across all series. Complementary NBO analysis suggests that this trend reflects stronger lactam-type donor–acceptor delocalisation in carbonylated N-heteroaromatic tautomers than in the corresponding imine, methylidene, or all-carbon benzenoid keto analogues. The model rationalises benzenoid phenols as boundary cases and predicts tautomeric preferences along the deamination pathway from melamine to cyanuric acid. These results establish a framework for predicting tautomeric preferences and clarify how aromatic-ring stabilisation is balanced against functional-group energetics.

## Abstract 2

Carbonyl formation and π-distortivity can outweigh aromatic stabilisation, yielding an additive rule for heteroaromatic tautomerism from DFT, SCGVB/GPF-EP and NBO analyses.

## Graphical abstract

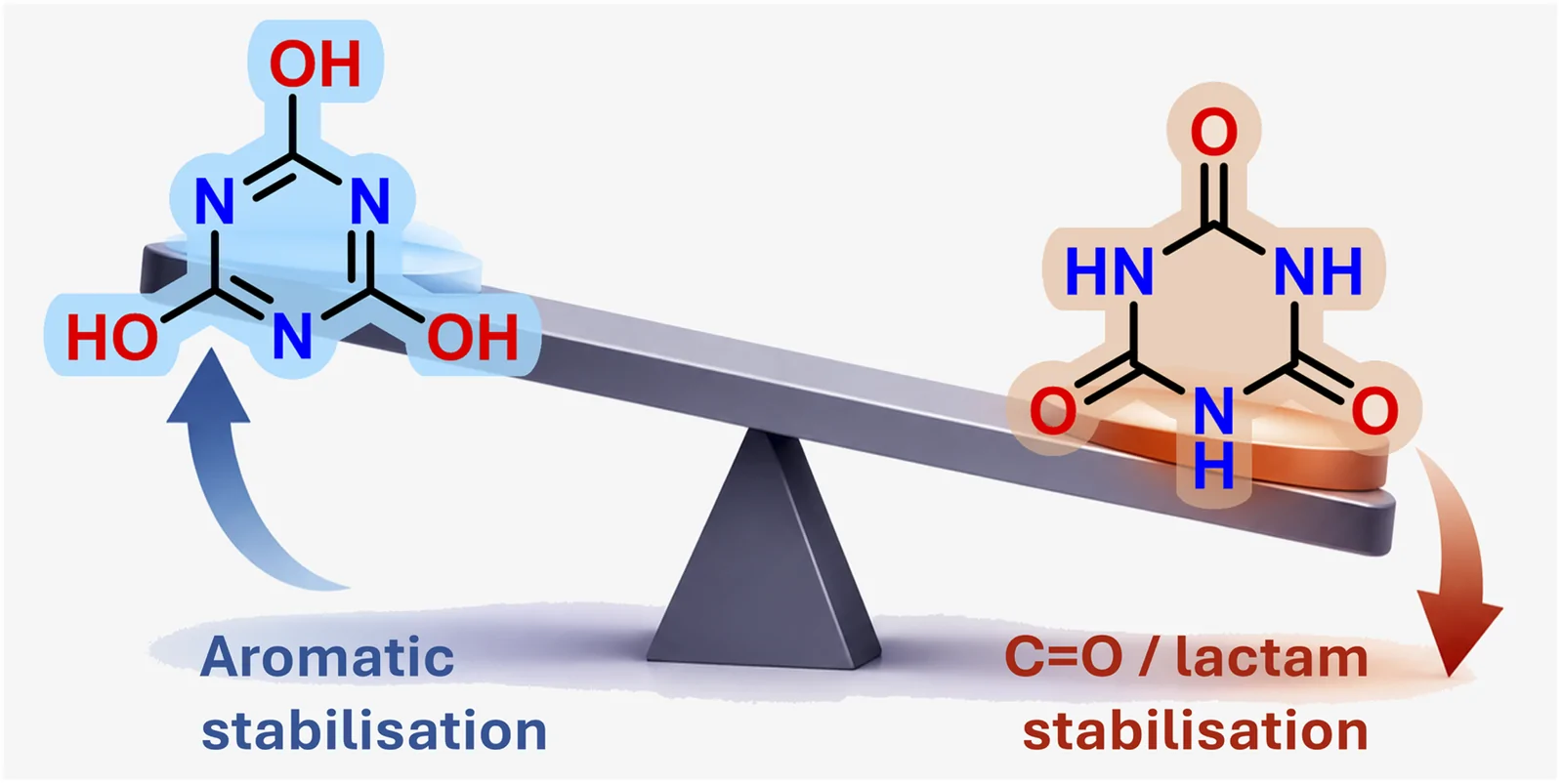

## 1 Introduction

Tautomerism is a fundamental chemical phenomenon that plays a key role in both organic and inorganic systems.^1,2^ Since the earliest days of structural chemical theory, tautomeric equilibria have shaped our understanding of molecular structure, stability, and reactivity.^3,4^ In organic chemistry, tautomerism influences measurable reactivity trends and synthetic outcomes—affecting, for example, polarographic reductions of carbonyl compounds,^5^ kinetic/thermodynamic regimes in halogenation,^6^ resonance-assisted intramolecular hydrogen bonding in heterocycles,^7^ Schiff base formation,^8^ azo–hydrazone chromophore switching,^9^ and acid-catalysed carbonyl activation in asymmetric catalysis.^10^ It also impacts drug-like scaffolds in solution,^11^ and extends to crystal phenomena, including self-inhibition of nucleation and growth^12^ and tautomerism-induced bending/twisting in biogenic crystals.^13^

In inorganic and organometallic chemistry, tautomerism reshapes coordination and reactivity: classic metal–carbonyl systems display C- *versus* O-bonding and µ-CO rearrangements,^14^ while tautomeric ligand designs can direct Pd-catalysed C–H hydroxylation with O_2_.^15^ Main-group platforms show analogous patterns, from phosphinylidene P(=O)H *⇌* P–OH interconversion with substituent-dependent kinetics^16^ to boride–borylene ligand tautomerism linked to remote C–C bond formation.^17^ In biological and medicinal settings, tautomerism underpins the activity of alkaloids,^18^ amino acids,^19^ and nucleic acids.^20–22^ Rare tautomers of DNA bases, for example, are directly implicated in mutagenic processes, with transient enol/imino forms hypothesised to mimic Watson–Crick geometry and promote misincorporation.^23^ This view is supported by high-resolution crystallography revealing near-isosteric mispairs,^24^ infrared photodissociation evidence for coexisting uracil tautomers,^22^ and multiscale simulations that map tautomerisation pathways from wobble mismatches to Watson–Crick-like geometries while clarifying environmental contributions.^25^ More broadly, tautomeric state control modulates binding modes and assay outcomes for drug-like scaffolds and small-molecule therapeutics, including programmes targeting neglected tropical diseases.^1,26^

One of the main conceptual challenges in tautomerism is its interplay with aromaticity. Aromaticity is among the most widely used yet debated ideas in chemistry, and lacks a universally accepted definition.^27–29^ While Hückel's rule offers a practical rule-of-thumb, its scope is limited.^29^ In general, equilibria involving an aromatic and a non-aromatic tautomer are expected to favour the aromatic species.^28,30^ Surprisingly, this expectation frequently breaks down: numerous tautomeric equilibria are dominated not by the aromatic species, but by carbonylated forms.^20,31,32^

Because tautomerism has long been linked to the concept of aromaticity,^33^ numerous theoretical approaches have sought to rationalise tautomeric preferences in these terms. Early work by Katritzky and co-workers introduced empirical resonance-energy schemes to quantify aromatic stabilisation across tautomeric series,^27,34–36^ while Schlegel and co-workers provided benchmark calculations for prototypical systems such as 2-pyridone.^37^ Complementary orbital-based analyses have also been employed. For example, Hejazi *et al.*^38^ used natural bond orbital (NBO)^39^ calculations to interpret the gas-phase preference for 2-hydroxypyridine (1a, Fig. 1a) in terms of steric and electrostatic effects within a Lewis-type framework. More generally, such approaches often invoke donor–acceptor interactions, including n(N) → π*(C=O) delocalisation in systems such as uracil derivatives.^40,41^ Alternative interpretations have also been proposed, including differing assessments of the roles of steric repulsion, hyperconjugation, and orbital interactions, although the relative importance of these effects remains debated and method-dependent.^42^ Despite this extensive literature, a simple and transferable framework for predicting tautomeric preferences across chemically diverse series remains lacking.

**Fig. 1:**
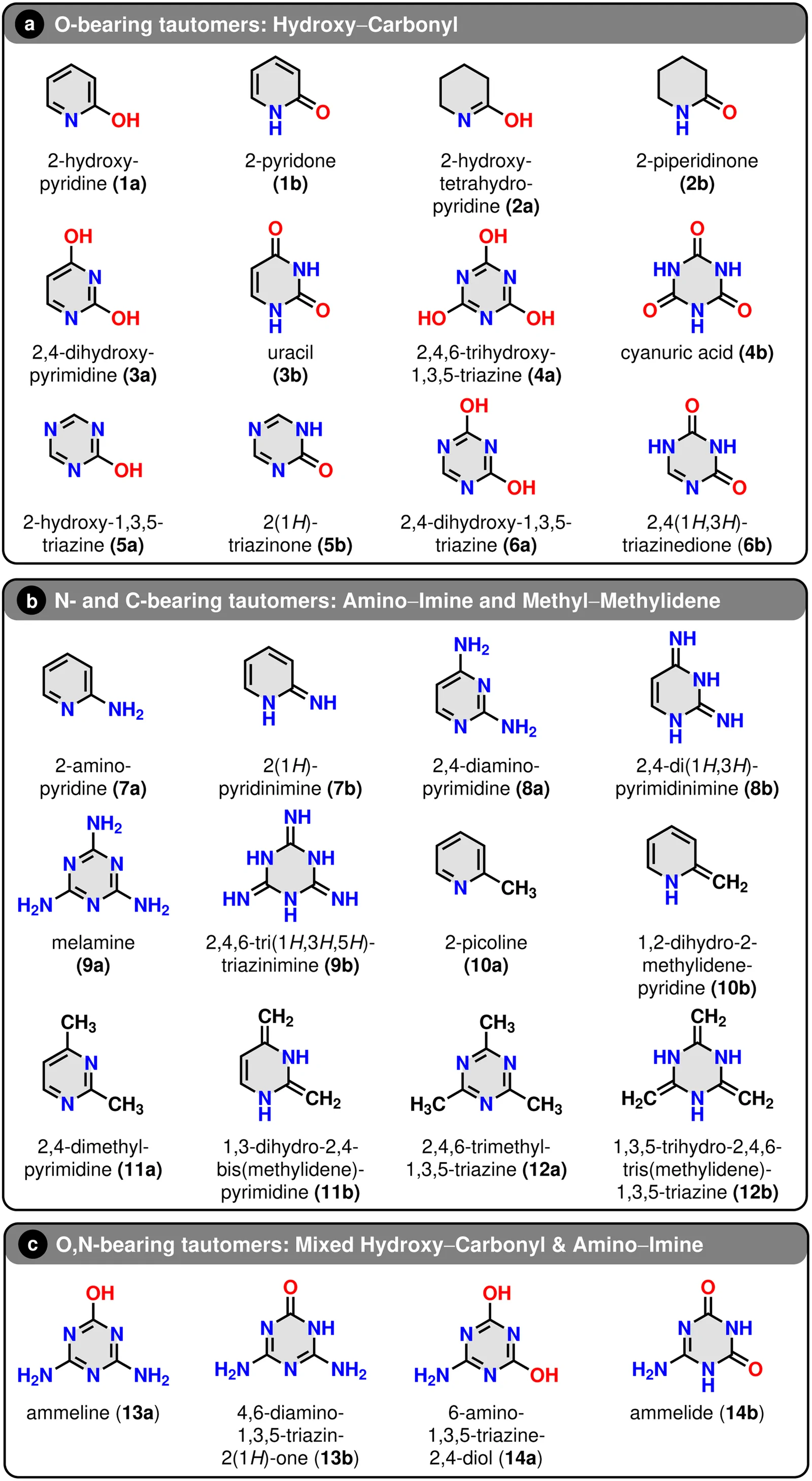
Molecular structures of the tautomeric systems investigated in this work: (a) hydroxy–carbonyl, (b) amino–imine and methyl–methylidene, and (c) mixed hydroxy–carbonyl and amino–imine systems.

The carbonyl group provides a powerful counterbalance to aromatic stabilisation. The bond dissociation energy of a C=O double bond, *ca.* 178 kcal mol^−1^ (Table S1), exceeds the combined strength of two single C–O bonds (*ca.* 86 kcal mol^−1^ each; *ca.* 172 kcal mol^−1^ in total), helping to explain the remarkable persistence of carbonyls in drugs, metabolites, and even in fundamental molecules such as carbon dioxide. These tabulated bond-enthalpy increments are not purely local quantities, but are averaged over different chemical structures and therefore implicitly contain stereoelectronic interactions present in those structures. In particular, the enhanced strength of the C=O bond has been associated with an anomeric-type 
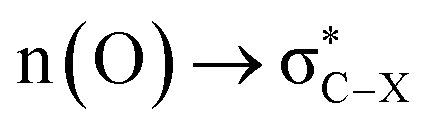
 interaction, which might contribute to the shortening and strengthening of the C=O bond relative to the corresponding C=N and C=C bonds.^43,44^ In nucleobases such as uracil and thymine, the equilibrium strongly favours the carbonylated tautomer, despite the availability of aromatic alternatives.^21^ The aromaticity of nitrogen-bearing bases has been extensively examined, and it has been suggested that the loss of aromaticity in non-aromatic tautomers may be compensated by stronger local bonding.^45^ This tension between heteroaromaticity and carbonyl stability defines the core problem of many tautomeric equilibria.

A prototypical case is the 2-hydroxypyridine (1a)/2-pyridone (1b) equilibrium (Fig. 1a), here labelled R1, which was first studied by Meyer in 1905.^46^ Experiments consistently show that the aromatic 1a is preferred in the gas phase, whereas the carbonylated 1b dominates in condensed phases.^47–54^ These systems have been characterised using mass spectrometry and IR/UV spectroscopy in the gas phase,^34,55,56^ p*K*_a_ measurements and NMR in aqueous solution,^57^ and X-ray diffraction and resonant inelastic X-ray scattering (RIXS) in the solid state.^54,58–60^ Another key equilibrium involves 2-aminopyridine (7a) and 2(1*H*)-pyridinimine (7b, Fig. 1b), which plays a role in heterocyclic reactivity,^37,61,62^ acid–base chemistry, and even RNA sequencing through thiolated nucleosides.^63^ Similar tautomeric preferences are also essential to base pairing and mutagenesis,^20,55^ and molecular aggregation in prebiotic chemistry.^64^

Predicting tautomeric preferences remains challenging because the balance between aromatic stabilisation and carbonyl stability is difficult to anticipate. Here, we introduce a simple qualitative model that captures the principal energetic contributions and enables reliable predictions across a range of heteroaromatic systems. A list of all molecules investigated in this work is shown in Fig. 1.

## 2 Computational details

Density functional theory (DFT) calculations were performed using the ORCA 6 software package.^65^ The choice of functional and basis set was validated against DLPNO-CCSD(T)/CBS calculations^66^ using the R1 equilibrium as the benchmark system, given its well-documented sensitivity to electronic-structure treatment. Among all functionals screened with the def2-TZVP basis set, ωB97M-D4rev^67^ delivered the smallest mean absolute deviation (MAD) relative to the coupled-cluster reference, with all range-separated functionals falling within chemical accuracy (Fig. S6a). A subsequent basis-set scan confirmed def2-TZVPP^68^ as the best compromise between cost and accuracy (Fig. S6b), and this combination was therefore adopted throughout. Geometry optimisations and harmonic frequency analyses were carried out at the ωB97M-D4rev/def2-TZVPP level to confirm stationary points as minima (no imaginary frequencies) and to obtain thermochemical functions within the ideal-gas, rigid-rotor harmonic-oscillator (RRHO) approximation. Unless stated otherwise, reported thermodynamic quantities are derived from these frequency calculations.

Valence bond calculations were performed using VB2000 interfaced with the GAMESS-US package.^69,70^ Chemical-bond analyses were carried out using the generalised product function energy partitioning (GPF-EP) method,^71^ as implemented in VB2000. This framework is particularly suited to the present problem because it provides direct access to the energetic contributions associated with self-consistently optimised non-orthogonal one-electron orbitals and the interference terms arising from their interactions.

GPF-EP applies the Ruedenberg density partitioning^72^ to a generalised product function (GPF),^73^ in which the N-electron wavefunction is expressed as an antisymmetrised product of strongly orthogonal group functions:1
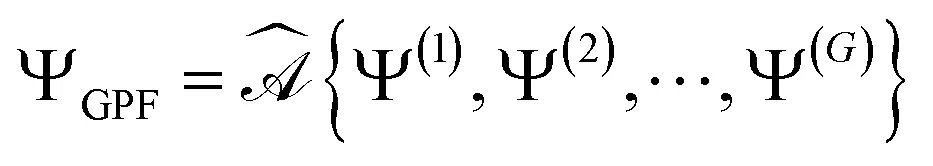


In Ruedenberg's formulation, the accumulation of electron density between bonded nuclei is a quantum-mechanical interference effect, analogous in origin to the density redistribution observed in the double-slit experiment.^72^ This density relocation by interference provides a quantum-mechanical basis for chemical bond formation and is illustrated schematically in Fig. 2. Within the GPF-EP framework, the total energy can therefore be separated into contributions with a quasi-classical interpretation, such as electrostatics and charge polarisation, and contributions arising from orbital interference. A useful feature of the method is that this partition can be applied to selected electron groups, such as individual 2c–2e bonds or larger delocalised groups, including the six π electrons of benzene. A complete derivation is given in Section S2 of the SI; here, we summarise the key elements.

**Fig. 2:**
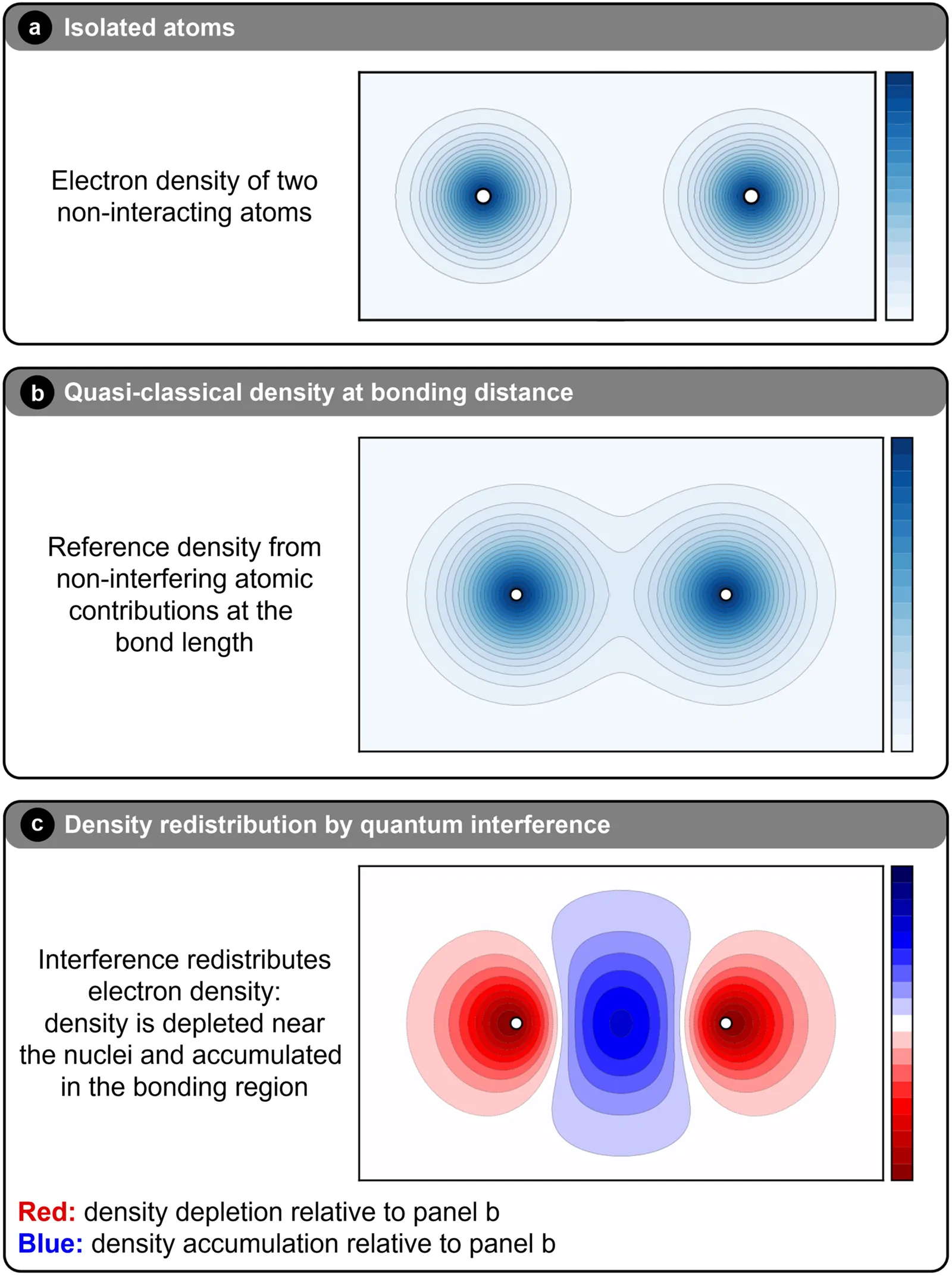
Schematic representation of the Ruedenberg density partition underlying the GPF-EP analysis. (a) Electron density of two isolated, non-interacting atoms. (b) Quasi-classical reference density obtained by placing the same atomic contributions at the bonding distance, without orbital interference. (c) Interference density, defined as the difference between the total density of the interacting system and the quasi-classical reference density in panel (b). Red regions indicate density depletion near the nuclei, whereas blue regions indicate density accumulation in the interatomic bonding region.

For each electron group µ, the one-electron density is exactly decomposed into a quasi-classical component, corresponding to the sum of squared individual orbital densities, and an interference component, arising from orbital non-orthogonality:

2 *ρ*^(µ)^ = *ρ*^(µ)^_QC_ + *ρ*^(µ)^_INT_

The interference density integrates to zero, but redistributes electron density within the molecule, most notably from the nuclear centres into bonding regions.

This density decomposition propagates through the pair density and, consequently, through the total energy:

3 *E*[TOT] = *E*[QC] + *E*[INT]

where *E*[QC] is the exchange-corrected quasi-classical energy, including independent-orbital density terms, Coulomb interactions, and inter-group Pauli exchange. Although exchange is quantum-mechanical in origin, it is included in *E*[QC] because it arises from antisymmetrisation between electron groups, rather than from the intra-group density redistribution caused by orbital interference. The *E*[INT] term is the total interference energy, associated with the energetic effect of density redistribution caused by constructive and destructive interference between non-orthogonal orbitals. In chemical terms, *E*[INT] captures the covalent stabilisation associated with bond formation, whereas *E*[QC] contains contributions that can be interpreted without invoking orbital interference, such as charge polarisation, electrostatic interactions and repulsion between electron pairs, among others.

All GPF calculations employed a two-group partition. One group comprised the six π electrons of the heteroaromatic ring, corresponding to the Hückel sextet in pyridines, diazines, and triazines. For the corresponding non-aromatic tautomers, the same group comprised the six electrons occupying the formal π framework derived from the ring, *i.e.*, the localised pairwise π bonds that replace the aromatic sextet. This group was treated with SCGVB, allowing all spin couplings among the six electrons. The second group contained the remaining electrons, including the σ framework, lone pairs, and exocyclic substituents, and was treated at the Hartree–Fock level. The interference analysis was carried out for the first group only, because previous work showed that energy changes associated with distortions of the σ framework are predominantly quasi-classical.^74^ Preliminary tests using alternative partitions and larger active groups indicated that this scheme provides a good compromise between wavefunction quality and the convergence demands of non-orthogonal SCGVB optimisation. The cc-pVDZ basis set was used throughout.

Before discussing the results, we briefly state the modern valence-bond picture of bonding in benzene-like aromatics that underpins our analysis. In this framework, the π system is described as a six-centre, six-electron (6c–6e) delocalised bond, an SCGVB description first articulated by Cooper *et al.*^75^ and subsequently refined in later work.^74,76^ From the interference perspective, adjacent *p*-like orbitals in the π framework exhibit net constructive interference around the ring, as reflected in both the pairwise interference energy terms and the corresponding interference density. The systems considered here are conventional cases in which the aromatic or non-aromatic character of the relevant tautomers is well established: the a-tautomers retain a cyclic six-π-electron framework, whereas the corresponding b-tautomers contain localised exocyclic double bonds and no longer retain the same delocalised ring pattern. Throughout this work, we therefore use the presence of a 6c–6e π bond with net constructive interference as an operational SCGVB/GPF-EP criterion to identify aromatic bonding patterns within these well-defined systems. We do not attempt to define or quantify aromaticity more broadly, given the continuing debate surrounding the term.^29^

## 3 Results and discussion

### 3.1 The 2-hydroxypyridine/2-pyridone equilibrium and related series

Following Katritzky's concept of group transferability,^34^ we hypothesise that tautomeric preferences can be rationalised by additive contributions from the ring and from the exchanging functional group. Focusing on the 2-hydroxypyridine (1a) *⇌* 2-pyridone (1b) equilibrium (Fig. 3, top), we define the energy difference as:

4 Δ*E* = *E*_1b_ − *E*_1a_ ≈ Δ*E*_R_ + Δ*E*_FG_

where Δ*E*_R_ captures changes in the ring π-framework (aromaticity gain/loss, ring strain, and polarisation) and Δ*E*_FG_ collects local bond-energy changes within the interconverted moiety, implicitly including differences in local effects such as electrostatic interactions and stereoelectronic effects involving the functional groups. For 1a → 1b, this moiety is (O–H, C=N) → (N–H, C=O); a rough estimate from tabulated bond enthalpies (Table S1) gives Δ*E*_FG_ ≈ −13.17 kcal mol^−1^, reflecting the strong C=O bond. The DFT gap is Δ*E*(1a → 1b) = 0.92 kcal mol^−1^ (Table S2, entry 1a → 1b), in line with experiment (0.77 kcal mol^−1^).^47^ Hence, Δ*E*_R_ is on the order of +13 kcal mol^−1^; specifically, Δ*E*_R_(1a → 1b) = +14.09 or +13.94 kcal mol^−1^ when using the DFT or experimental Δ*E*(1a → 1b), respectively—nearly compensating the carbonyl stabilisation. We use this simple decomposition to rationalise trends across related heteroaromatic series and to formulate an additive rule for substituent effects.

**Fig. 3:**
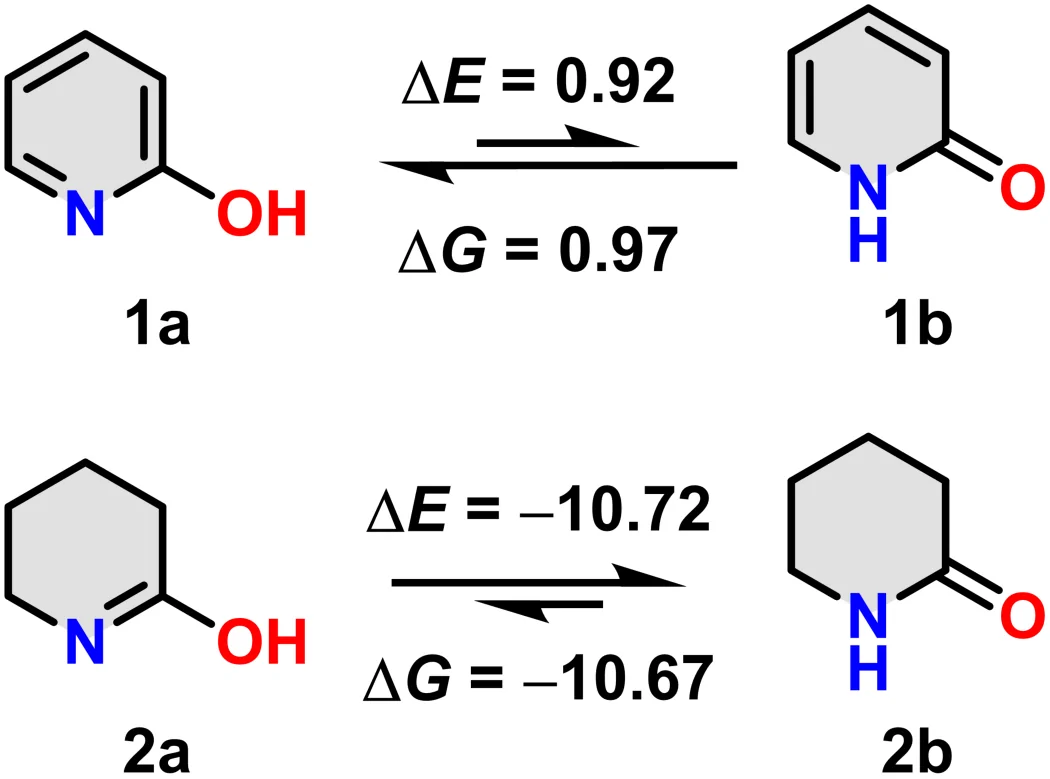
Energetics of the 2-hydroxypyridine (1a) *⇌* 2-pyridone (1b) equilibrium and its saturated reference, 2-hydroxytetrahydropyridine (2a) *⇌* 2-piperidinone (2b). Energies (in kcal mol^−1^) at the ωB97M-D4rev/def2-TZVPP level.

An independent estimate of Δ*E*_R_(1a → 1b) is obtained by comparing the pyridine pair with its non-aromatic analogue, the δ-lactim/δ-lactam equilibrium in 2-hydroxytetrahydropyridine (2a) to 2-piperidinone (2b; Fig. 3, bottom). In this saturated reference, the ring contribution is minimised, so the observed gap approximates the functional-group term, Δ*E*_FG_ ≈ Δ*E*_sat_. Accordingly:

5 Δ*E*_R_ ≈ Δ*E*_aromatic_ − Δ*E*_sat_

with Δ*E*_aromatic_ = Δ*E*(1a → 1b) and Δ*E*_sat_ = Δ*E*(2a → 2b). Using the tabulated values (Table S2, entries 1a → 1b and 2a → 2b) gives Δ*E*_R_(1a → 1b) ≈ +11.64 kcal mol^−1^, close to the bond-enthalpy-based estimate. While part of Δ*E*_R_(1a → 1b) can be ascribed to loss of the aromatic π pattern, residual ring-structure effects (*e.g.*, polarisation, geometry, hyperconjugation) also contribute. In net, the stabilisation from carbonyl formation is almost exactly offset by ring-framework penalties, yielding the observed small Δ*E*(1a → 1b).

We next analyse the uracil tautomeric pair: 2,4-dihydroxypyrimidine (3a, lactim) and uracil (3b, lactam). Assuming additivity for the functional-group term, introducing an additional (C=O, N–H) unit relative to the 1a/1b pair contributes approximately −13.17 kcal mol^−1^ to Δ*E*. With the convention Δ*E* = *E*_3b_ − *E*_3a_, this gives Δ*E*(3a → 3b) ≈ Δ*E*(1a → 1b) − 13.17 = −12.25 kcal mol^−1^, in excellent agreement with the DFT value, Δ*E*(3a/3b) = −11.11 kcal mol^−1^ (Table S2, entry 3a → 3b). Despite the simplicity of the additivity model, this consistency supports using the ring/functional-group decomposition to anticipate tautomeric preferences across related systems.

Although the present analysis focuses on gas-phase tautomeric preferences, the reader may be interested in the effects of solvation on tautomeric equilibria. It has long been established that, for equilibria such as R1, solvents with high dielectric constants favour the carbonylated form, whereas solvents with low dielectric constants tend to maintain a more balanced distribution of tautomers (see ref. 77). Experimentally, the dominance of the lactam form 1b in aqueous solution prompted us to examine the effect of implicit solvation on the prototypical R1 equilibrium. As shown in Fig. S2a, inclusion of water *via* the CPCM^78^ and SMD^79^ continuum models reverses the slight gas-phase preference (Δ*E* = +0.92 kcal mol^−1^ in favour of 1a), yielding Δ*E* ≈ −4 kcal mol^−1^ in favour of the carbonylated tautomer 1b in both solvation models. This result is consistent with experimental data obtained in aqueous solution.^80,81^ We attribute this reversal to differential electrostatic stabilisation: the larger dipole moment and enhanced hydrogen-bond-accepting capacity of the lactam form are better accommodated in a polar protic environment. This changes the effective magnitude of Δ*E*_R_ while leaving Δ*E*_FG_ comparatively unchanged (vide Fig. S2b). Consequently, the functional-group term changes only modestly in polar solvents, whereas the aromatic ring penalty is affected by dielectric screening and polarisation effects.

A comprehensive solvation analysis of all tautomeric pairs is beyond the scope of the present work. We emphasise that the predictive model developed here primarily describes gas-phase tautomeric preferences, and its direct application to solution-phase equilibria requires explicit treatment of solvation effects. Nevertheless, these results confirm that the ring/functional-group decomposition framework remains qualitatively valid across phases: solvation changes the effective ring/polarisation contribution in favour of the carbonylated tautomer, while the functional-group term remains comparatively transferable.

Finally, the additive framework developed here is most directly applicable to six-membered N-containing heteroaromatic rings, for which Δ*E*_R_ is well-defined and Δ*E*_FG_ can be approximated using transferable bond-enthalpy increments. Extension to n-membered heteroaromatic rings would require reparameterisation of Δ*E*_R_ due to differences in electronic structure and conformational effects. Moreover, the transferability of Δ*E*_FG_ is expected to be greatest when functional groups are directly conjugated to, or in close electronic communication with, the aromatic ring. Nevertheless, the underlying principle that cumulative formation of multiple C=O/N–H units can overcome aromatic stabilisation and shift equilibria towards carbonylated tautomers is expected to remain broadly applicable.

### 3.2 Carbonyls *vs.*6c–6e bonds

Fig. 4 summarises the relative energies for homologous tautomeric sets as the number and type of exchanging groups increase. The figure is organised as follows: (a) hydroxy–carbonyl pairs 1a/1b, 3a/3b, 4a/4b, increasing the number of OH groups in the enolic tautomer from 1 to 3 while simultaneously moving from pyridine to triazine; (b) hydroxy–carbonyl pairs on a fixed triazine core with a stepwise increase in OH groups (pairs 5a/5b, 6a/6b, 4a/4b); (c) amino–imine pairs traversing the heteroaromatic scaffold from pyridine to triazine (pairs 7a/7b, 8a/8b, 9a/9b); and (d) methyl–methylidene pairs, increasing from one to three CH_3_ substituents (pairs 10a/10b, 11a/11b, 12a/12b), while likewise progressing from pyridine to triazine. In each case, the upper portion shows the calculated Δ*E* and Δ*G* for the individual tautomeric transformations together with the corresponding structures, and the lower portion plots Δ*E* against the number of exchanging functional groups. Each transformation is labelled as RX, where X matches the label of the corresponding tautomeric pair. While each series is limited, chemical consistency of the slopes across structurally related series provides qualitative support for the additive model. We present these correlations as illustrations of the additivity principle rather than statistically validated quantitative predictions.

**Fig. 4:**
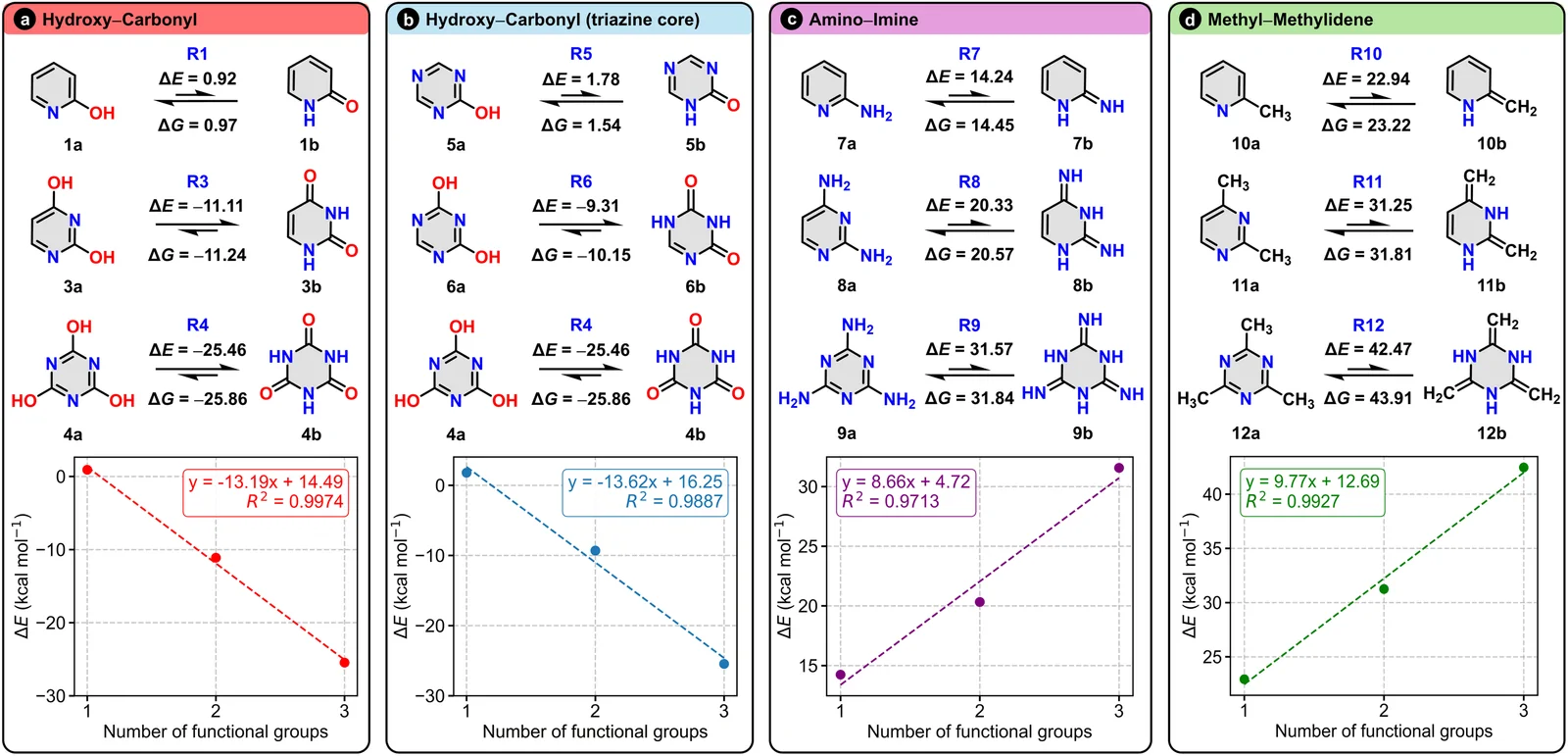
Relative energies (in kcal mol^−1^) for homologous tautomeric sets as the number and type of exchanging groups increase. (a) Hydroxy–carbonyl pairs 1a/1b, 3a/3b, 4a/4b; (b) hydroxy–carbonyl pairs on a fixed triazine core 5a/5b, 6a/6b, 4a/4b; (c) amino–imine pairs 7a/7b, 8a/8b, 9a/9b; (d) methyl–methylidene pairs 10a/10b, 11a/11b, 12a/12b. In each set, the upper part shows calculated Δ*E* and Δ*G* with structures; the lower plots Δ*E versus* the count of exchanging groups. Reactions are labelled RX, with X matching the tautomeric pair. Energies (in kcal mol^−1^) were obtained at the ωB97M-D4rev/def2-TZVPP level.

When only a single tautomeric group is present (*e.g.*, R1, R5), the aromatic tautomer is only marginally favoured; the small Δ*E*/Δ*G* reflects near-cancellation between the local stabilisation gained on forming a C=O/N–H unit and the penalty associated with perturbing the ring π-framework. Introducing additional O-bearing groups (hydroxy–carbonyl) drives a monotonic shift towards the carbonylated structures, both along the scaffold change from pyridine to triazine (R1 → R3 → R4) and on a fixed triazine core (R5 → R6 → R4). In the lower panels, Δ*E* varies approximately linearly with the number of (C=O, N–H) units, consistent with an additive model. Linear fits give slopes of −13.2 kcal mol^−1^ for (a) and −13.6 kcal mol^−1^ for (b), an average of −13.4 kcal mol^−1^ per C=O/N–H formed. Accordingly, inversion of the preference typically requires two such units. The corresponding Δ*G* values follow the same trend at ambient temperature, indicating that entropic contributions are modest relative to the dominant enthalpic term.

By contrast, for N- and C-bearing pairs (amino–imine; methyl–methylidene), the aromatic tautomer is reinforced: exocyclic C=NH or C=CH_2_ formation is disfavoured relative to retention of an NH_2_ or CH_3_ donor on an aromatic ring, which better supports the ring π-system and avoids the additional polarisation/strain associated with extra exocyclic multiple bonds. Quantitatively, the Δ*E*-count correlations display positive slopes of +8.7 and +9.8 kcal mol^−1^ for (c) and (d), respectively. Across these sets, Δ*E* spans 14.24–31.57 kcal mol^−1^ for (c) and 22.94–42.47 kcal mol^−1^ for (d), and the corresponding Δ*G* values closely track Δ*E* at ambient temperature, indicating minimal entropic modulation of the overall trend. As a result, the non-aromatic alternatives remain higher in energy throughout these series. A related boundary case is provided by benzenoid phenols: carbonyl formation stabilises the keto forms, but the net gas-phase Δ*E* remains in favour of the enol across phenol, resorcinol, and phloroglucinol; the enol–keto gap decreases as the number of OH groups increases but does not invert under these conditions (Table S3 and Fig. S1), as discussed further below.^82^

Building on these additive trends, we next examine how the energy balance is resolved within the SCGVB/GPF-EP framework. Fig. 5 decomposes the total energy (*E*[TOT]) into quasi-classical (*E*[QC]) and interference (*E*[INT]) contributions for representative tautomerisation reactions. Across all cases analysed, the two components act in opposition: the *E*[QC] term consistently favours an aromatic arrangement of the ring π system, whereas the *E*[INT] term favours localisation into stronger 2c–2e bonds, including carbonyl and other exocyclic double bonds. In other words, in the hypothetical absence of quantum interference, all series would prefer the aromatic a-forms; the preference for carbonyl-bearing tautomers, where observed, is enabled by the stabilising *E*[INT] contribution. This picture accords with π-distortivity:^83,84^ interference promotes double-bond formation and bond-length alternation, while *E*[QC] resists localisation. The corresponding pairwise p-orbital interference matrices and interference-density plots, reported in the SI, show this reorganisation explicitly: aromatic tautomers retain a delocalised 6c–6e signature, whereas carbonylated tautomers display localised patterns consistent with bond alternation. It also echoes our previous SCGVB/GPF-EP analysis of benzene,^74^ wherein interference stabilises Kekulé-type structures through the formation of three strong 2c–2e bonds, consistent with experimental indications of π-driven distortivity.^85,86^ Large shifts in tautomer preference arise when the interference gain from forming localised bonds (notably C=O) outweighs the *E*[QC] bias towards the aromatic structure. Small residual discrepancies in sign or magnitude in cases with small energy differences reflect modest missing dynamic correlation in the SCGVB description without altering these qualitative conclusions.

**Fig. 5:**
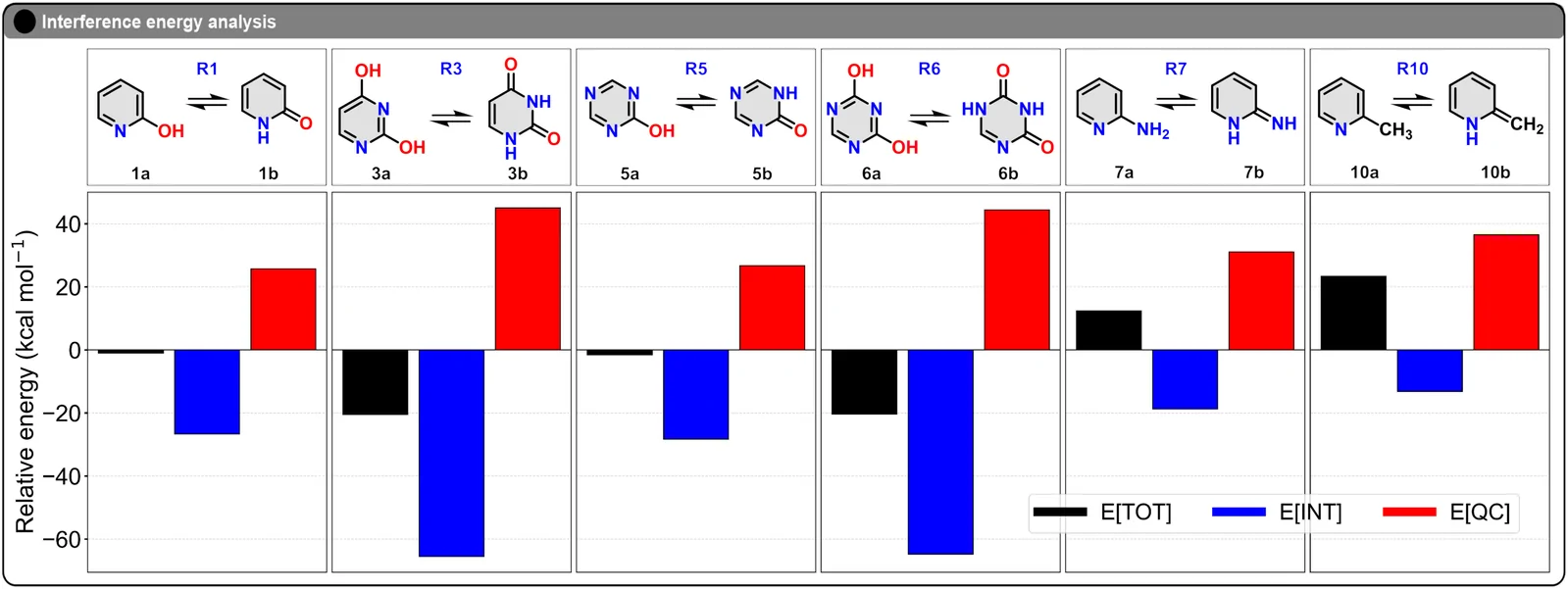
Interference-energy analysis of selected tautomeric equilibria within the SCGVB/GPF-EP framework. The total energy is partitioned into quasi-classical, *E*[QC], and interference, *E*[INT], contributions. In the absence of the interference contribution, the aromatic tautomers would prevail across the series. Numerical values for the total decomposition are provided in Tables S4 and S5, while the corresponding interference-density plots and pairwise p-orbital interference terms are shown in Fig. S3, S4 and Table S6.

To understand why carbonyl formation, but not imine or methylidene formation, can overturn the aromatic preference, we focus on the singly substituted equilibria R1, R7, and R10 (Fig. 6). These three systems undergo the same formal conversion from an aromatic a tautomer to a non-aromatic b tautomer, but differ in the exocyclic unit formed: C=O, C=NH, or C=CH_2_. The SCGVB orbitals of 1a, 7a, and 10a show the six non-orthogonal orbitals that combine to form the aromatic 6c–6e π bond (Fig. 6a). The close similarity among these orbital patterns indicates that the three aromatic tautomers share a comparable electronic structure before tautomerisation. We note that SCGVB orbitals are allowed to polarise, thereby incorporating simple ionic-like contributions into the wavefunction,^87^ which improves the quantitative description without changing the qualitative bonding assignment.

**Fig. 6:**
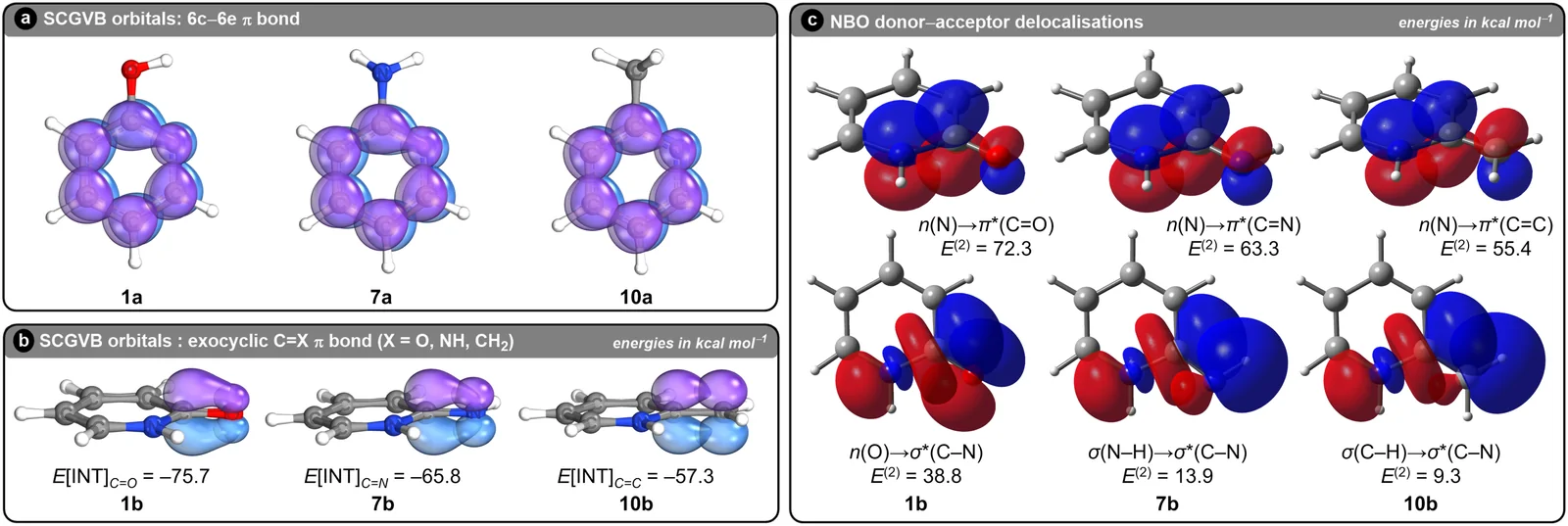
SCGVB/GPF-EP and NBO analysis of the singly substituted equilibria R1, R7, and R10. (a) SCGVB orbital representation of the aromatic 6c–6e π bond in 1a, 7a, and 10a. (b) Localised exocyclic C=X π bonds in 1b, 7b, and 10b (X = O, NH, CH_2_), with the corresponding pairwise interference energies, *E*[INT]_C=X_, in kcal mol^−1^. Negative values denote stabilising interference contributions. (c) Selected NBO donor–acceptor delocalisations in the non-aromatic tautomers. The upper row shows resonance-type n(N) → π*(C=X) interactions associated with the exocyclic C=X unit, whereas the lower row shows the principal σ*(C–N)-acceptor interactions used in the stereoelectronic comparison. Second-order perturbation energies, *E*^(2)^, are given in kcal mol^−1^ as positive stabilisation energies.

In the corresponding b tautomers, this 6c–6e arrangement is replaced by a localised exocyclic C=X π bond (Fig. 6b). The pairwise interference energies for these exocyclic bonds become progressively less stabilising from 1b to 7b and 10b, with *E*[INT]_C=X_ values of −75.7, −65.8, and −57.3 kcal mol^−1^, respectively. This local trend parallels the total Δ*E*[INT] values for the corresponding tautomerisations, which decrease in stabilising magnitude from R1 to R7 and R10 (−26.7, −21.1, and −14.3 kcal mol^−1^, respectively). Carbonyl formation therefore gives the largest interference stabilisation among the three exocyclic units, although the final tautomeric preference still depends on its balance with the quasi-classical penalty. The other side of the balance is the quasi-classical term. In all three reactions, *E*[QC] favours the aromatic tautomer, with Δ*E*[QC] values of +25.7, +34.6, and +40.3 kcal mol^−1^ for R1, R7, and R10, respectively. The slight preference for 1b therefore arises only because the interference stabilisation is large enough to overcome this quasi-classical penalty. In R7 and R10, the interference term is weaker and the quasi-classical penalty is larger, so the aromatic tautomers remain preferred.

We next used NBO analysis to relate these trends to a more conventional stereoelectronic description of tautomerism. Such analyses are often framed in terms of hyperconjugation and donor–acceptor delocalisation, in which occupied lone-pair, σ, or π donor orbitals interact with antibonding acceptor orbitals. This stereoelectronic language has been emphasised by Alabugin and co-workers in the context of hyperconjugation, anomeric effects, donor–acceptor orbital interactions, the stereoelectronic role of oxygen, and the stabilisation of carbonyl, amide, and urea motifs.^40,43,44,88^ The NBO results provide complementary explanation for why carbonyl formation has different consequences in N-heteroaromatic and all-carbon benzenoid systems. In the N-heteroaromatic series, carbonylated tautomers become preferred once two or more C=O/N–H units are present. In the benzenoid phenol series, carbonyl formation also reduces progressively the enol–keto energy gap but is not sufficient to invert the tautomeric preference under the gas-phase conditions considered here (Table S3 and Fig. S1).

The selected NBO interactions support two complementary comparisons (Fig. 6c and Table S7). First, the resonance-type n(N) → π*(C=X) interactions in the non-aromatic N-heteroaromatic tautomers decrease from the lactam-type carbonyl tautomer 1b (*E*^(2)^ = 72.3 kcal mol^−1^) to the imine tautomer 7b (*E*^(2)^ = 63.3 kcal mol^−1^) and the methylidene tautomer 10b (*E*^(2)^ = 55.4 kcal mol^−1^). Second, the all-carbon benzenoid keto tautomer provides two useful reference points (Fig. S5 and Table S7). Its dominant conjugative interaction is the enone-like π(C=C) → π*(C=O) donation (*E*^(2)^ = 29.1 kcal mol^−1^), which is much smaller than the lactam-type n(N) → π*(C=O) interaction in 1b. A more site-specific comparison is obtained by replacing the ring N-lone-pair donor in 1b with the benzenoid CH_2_ unit present in the keto tautomer of phenol. The corresponding σ(C–H) → π*(C=O) hyperconjugative donations are only 7.0 and 7.2 kcal mol^−1^, or 14.2 kcal mol^−1^ combined. Thus, replacing the lactam N-lone-pair donor by CH_2_ removes a strong resonance interaction, providing a stereoelectronic reason why carbonyl formation is less able to overcome aromaticity in the benzenoid series.

The selected σ*(C–N)-acceptor interactions shown in Fig. 6c give a related but less dramatic comparison. Within the non-aromatic b-tautomers, the carbonylated N-heteroaromatic tautomer 1b has the strongest interaction, n(O) → σ*(C–N) (*E*^(2)^ = 38.8 kcal mol^−1^). The corresponding interactions are weaker in the imine and methylidene tautomers: σ(N–H) → σ*(C–N) in 7b (*E*^(2)^ = 13.9 kcal mol^−1^) and σ(C–H) → σ*(C–N) in 10b (*E*^(2)^ = 9.3 kcal mol^−1^). The carbonylated tautomer 1b also has a stronger n(O) → σ*(C–N) interaction than the analogous n(O) → σ*(C–C_sp^3^_) interaction in the all-carbon benzenoid reference (*E*^(2)^ = 29.3 kcal mol^−1^). The imine case is conformationally sensitive. The higher-energy imine isomer 7b′ is included in Table S7 because it allows the more directly comparable n(N) → σ*(C–N) interaction; this term has *E*^(2)^ = 21.8 kcal mol^−1^, still substantially smaller than the corresponding carbonyl interaction in 1b.

A differential comparison between the aromatic a and non-aromatic b tautomers gives the same qualitative trend. For the selected σ*(C–N)-acceptor interactions associated with the tautomerising unit, the increase in *E*^(2)^ is largest for 1a → 1b (+30.9 kcal mol^−1^), smaller for 7a → 7b (+8.2 kcal mol^−1^), and smallest for 10a → 10b (+4.4 kcal mol^−1^). Because NBO *E*^(2)^ values are perturbative donor–acceptor descriptors rather than thermodynamic energy components, this comparison is used qualitatively; nevertheless, it supports the same ordering inferred from the SCGVB/GPF-EP analysis.

This stereoelectronic picture complements the SCGVB/GPF-EP energy decomposition. Within the SCGVB/GPF-EP framework, carbonyl formation gives strong local interference stabilisation through formation of localised 2c–2e C=O bonds, whereas *E*[QC] favours retention of the aromatic tautomer. In N-heteroaromatic systems, the additional donor–acceptor stabilisation associated with lactam-type n(N) → π*(C=O) resonance and n(O) → σ*(C–N) interactions helps shift this balance towards the carbonylated tautomer.^44^ In all-carbon benzenoid systems, the corresponding site-specific CH_2_-to-carbonyl hyperconjugation and 
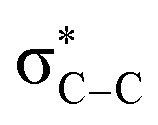
-based interactions are weaker, so carbonyl formation stabilises the keto forms but is not sufficient to overcome the aromatic preference. In the imine and methylidene series, the σ*(C–N)-acceptor interactions increase only modestly upon tautomerisation, by +8.2 and +4.4 kcal mol^−1^, respectively, compared with +30.9 kcal mol^−1^ for the carbonylated pair. These smaller stereoelectronic gains are therefore insufficient to offset the weaker interference stabilisation and larger quasi-classical penalties in R7 and R10. Thus, the observed tautomeric preference is determined by the relative magnitude of these opposing contributions, rather than by aromaticity, carbonyl strength, or donor–acceptor effects alone.

### 3.3 Tautomeric preferences along a deamination pathway

To illustrate the predictive power of the additive model, we examine the successive deamination pathway of melamine (9a) to ammeline (13a), ammelide (14b), and cyanuric acid (4b).^89^ Each intermediate admits hydroxy–carbonyl (lactim–lactam) tautomerism on the 1,3,5-triazine core, and in the literature these metabolites are often sketched interchangeably in either form. In practice, however, a single tautomer dominates for each species, and using the wrong depiction propagates confusion in reaction schemes. A consolidated scheme with the computed electronic and free-energy differences for each tautomeric transformation and deamination step is provided in Fig. 7.

**Fig. 7:**
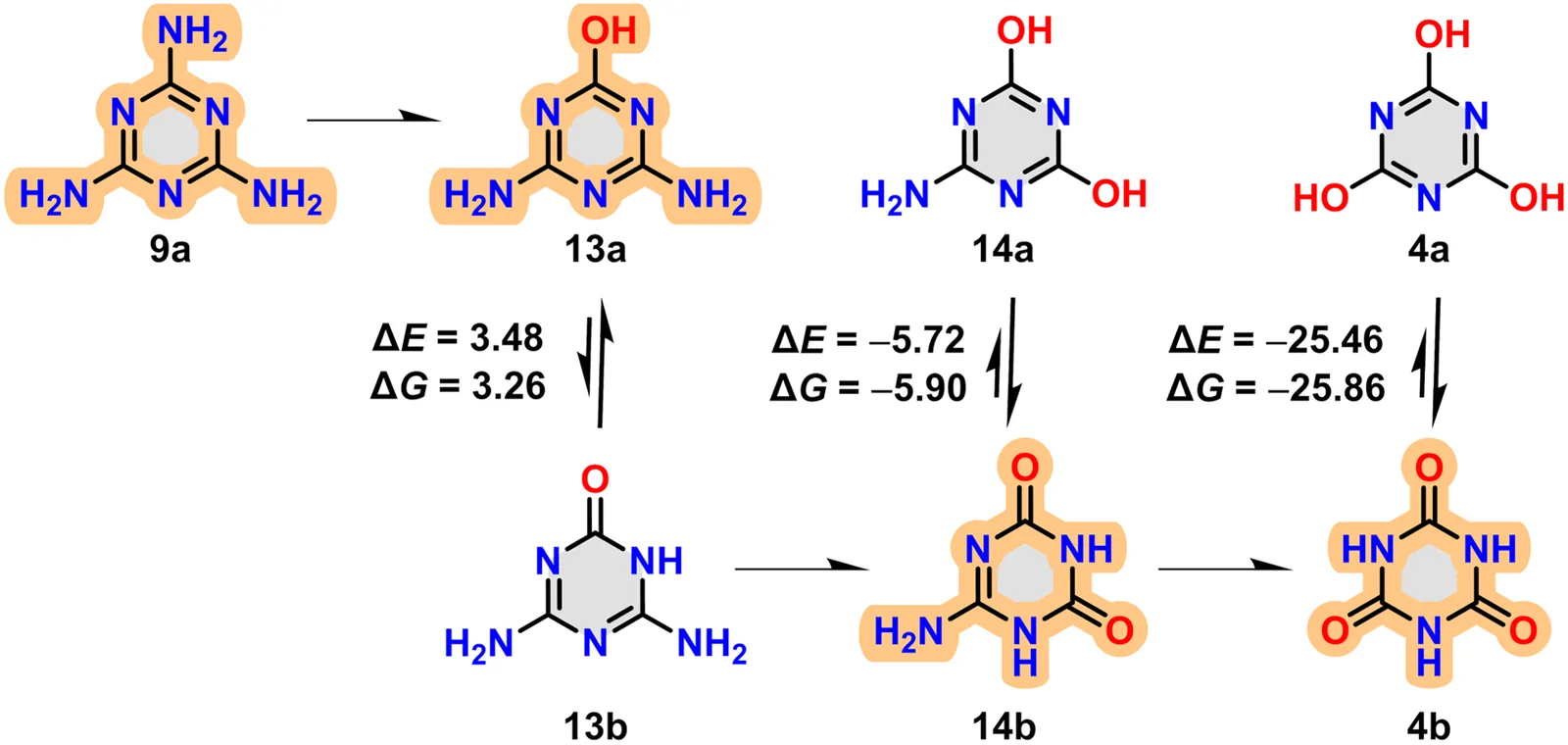
Successive deamination of melamine on a 1,3,5-triazine core, showing dominant tautomers and computed Δ*E*/Δ*G* for each tautomerisation and deamination step. Amino/imine substituents stabilise the aromatic tautomer, whereas hydroxy/carbonyl units favour the localised, non-aromatic (lactam) tautomer; two C=O/N–H units typically overturn the aromatic preference. Accordingly, melamine (9a) and ammeline (13a) are aromatic; ammelide (14b) and cyanuric acid (4b) are non-aromatic. Energies (in kcal mol^−1^) at the ωB97M-D4rev/def2-TZVPP level.

Within this framework, amino/imine substituents stabilise the aromatic tautomer, whereas hydroxy/carbonyl units favour the localised, non-aromatic alternative; in practice, two C=O/N–H units are usually required to overturn the aromatic preference. Applied to this series: melamine (three NH_2_) strongly prefers the aromatic tautomer; ammeline (two NH_2_ plus one OH) remains aromatic; ammelide (one NH_2_ plus two C=O/N–H) crosses over to the non-aromatic, lactam-dominated tautomer; and cyanuric acid (three C=O/N–H) is firmly non-aromatic. These assignments align with the trends quantified above and rationalise which depictions are chemically faithful at each stage of the pathway.

## 4 Conclusions

In summary, we investigated heteroaromatic tautomeric equilibria and showed that their preferences arise primarily from the relative energetic balance between the exchanging functional groups and the aromatic ring framework. A simple ring/functional-group model rationalises the 2-hydroxypyridine/2-pyridone benchmark and extends across related series, where the introduction of C=O/N–H units shifts the equilibrium in an approximately additive manner, typically requiring two such units to overturn the aromatic preference. In contrast, amino/imine and methyl/methylidene exchanges reinforce the aromatic tautomer, consistent with the disfavouring of exocyclic C=NH and C=CH_2_ formation.

The SCGVB/GPF-EP analysis provides a mechanistic interpretation of these trends. Specifically, *E*[QC] favours aromatic arrangements associated with a 6c–6e π-bonding pattern, whereas *E*[INT] favours the formation of localised, electron-precise 2c–2e bonds, particularly C=O units. This opposition provides an energy-decomposition expression of π-distortivity: interference, which encapsulates covalent contributions, stabilises bond localisation and bond-length alternation, while the quasi-classical term favours retention of the delocalised aromatic arrangement. The observed tautomeric preference therefore reflects the balance between these contributions, with carbonyl groups distinguished by a sufficiently large interference stabilisation to overcome the quasi-classical preference for aromaticity. The accompanying NBO analysis suggests a complementary stereoelectronic interpretation, where lactam-type carbonylated tautomers benefit from stronger n(N) → π*(C=O) resonance and associated σ*(C–N)-acceptor interactions than the corresponding imine, methylidene, or all-carbon keto references.

The additive model is most directly applicable to six-membered N-heteroaromatic systems, but it also successfully explains energy differences between tautomeric forms in other aromatic series. In benzenoid phenols, for example, a similar regime is observed, although it leads to a different outcome: carbonyl formation also stabilises the keto forms, but the aromatic tautomer remains preferred even as the enol–keto gap decreases with increasing hydroxyl substitution, reflecting the larger penalty associated with disrupting an all-carbon π framework. The same balance between quasi-classical and interference contributions therefore applies, but the relative magnitudes of these terms lead to a different tautomeric outcome.

Finally, the model rationalises the evolution of tautomeric preferences along the deamination pathway from melamine to cyanuric acid. Melamine and ammeline retain aromatic structures, whereas ammelide and cyanuric acid cross over to the carbonylated, non-aromatic forms once two or more C=O/N–H units are present. Although quantitative values may vary with improved correlation and solvation, this approach offers a simple and transferable basis for understanding and predicting tautomeric preferences across related molecular scaffolds.

## Author contributions

L. A.: methodology; software; formal analysis; investigation; data curation; visualisation; writing – original draft; writing – review & editing. F. F.: software; validation; resources; visualisation; supervision; writing – original draft; writing – review & editing; funding acquisition. P. M. E.: conceptualisation; resources; writing – review & editing; supervision. T. M. C.: methodology; software; writing – review & editing; supervision; project administration; funding acquisition.

## Conflicts of interest

There are no conflicts to declare.

## Supplementary Material

SC-OLF-D6SC03354G-s001

## Data Availability

The data supporting this article have been included as part of the supplementary information (SI). Supplementary information: bond enthalpies; energies for all tautomers; complete SCGVB/GPF-EP energy analysis; NBO results; cartesian coordinates. See DOI: https://doi.org/10.1039/d6sc03354g.
