# Supplementary material for: When carbonyls beat aromaticity: an additive framework for predicting tautomeric preferences
Source: Chem Sci. 2026 Oct 13. Online ahead of print. doi: 10.1039/d6sc03354g (PMC13684874; doi:10.1039/d6sc03354g)
Supplement: Supplementary file 1 [file SC-OLF-D6SC03354G-s001.pdf]

Supplementary Information (SI) for Chemical Science.
This journal is © The Royal Society of Chemistry

Supporting Information

When carbonyls beat aromaticity: An additive framework for predicting tautomeric preferences

Lucas Araujo,[a‡] Felipe Fantuzzi,*[b] Pierre M. Esteves*[a] and Thiago M. Cardozo*[a]

[a] Instituto de Química, Universidade Federal do Rio de Janeiro, Av. Athos da Silveira Ramos, 149, CT, A-622, Cid. Univ., Rio de Janeiro, RJ 21941-909, Brazil. E-mails: (T.M.C.) thiago@iq.ufrj.br; (P.M.E.) pesteves@iq.ufrj.br.

[b] Chemistry and Forensic Science, School of Natural Sciences, University of Kent, Park Wood Rd, Canterbury CT2 7NH, UK. E-mail: f.fantuzzi@kent.ac.uk.

‡ Present address: Center for Theoretical Chemistry, Faculty of Chemistry and Biochemistry, Ruhr University Bochum, Universitätsstraße 150, 44801 Bochum, Germany.

Contents

S1 Energetics data **S2**

S2 Quantum interference and the GPF-EP method **S5**
- S2.1 Quantum interference in chemical systems . . . S5
- S2.2 The GPF wavefunction . . . S5
- S2.3 Reduced density matrices . . . S6
- S2.4 Energy partitioning . . . S7
- S2.5 Limiting case and relevance to tautomeric preferences . . . S9

S3 SCGVB and GPF-EP data **S11**

S4 NBO donor–acceptor analysis **S14**

S5 DFT validation **S34**

S6 VB2000 example **S36**

S7 DFT optimised cartesian coordinates and vibrational frequencies **S37**

S1 Energetics data

Table S1. Average bond enthalpies obtained from reference experimental thermochemical data in kcal mol^{-1}. Taken from P. W. Atkins, J. De Paula and J. Keeler, *Atkins' physical chemistry*, Oxford University Press, 2023.

Bond	ΔH
C=O	177.5
C=N	146.4
O–H	110.6
N–H	92.67

Table S2. Absolute energies and free energies computed at the ωB97M-D4rev/def2-TZVPP level of all the heterocyclic tautomers investigated in the work. Energy differences in kcal mol^{-1}.

	tautomer *a*		tautomer *b*			
	E	G	E	G	ΔE	ΔG
1a→1b	−323.742 699 9	−323.677 331 4	−323.741 238 1	−323.675 780 3	0.92	0.97
2a→2b	−326.147 734 3	−326.036 946 1	−326.164 826 9	−326.053 957 5	−10.73	−10.67
3a→3b	−415.092 704 3	−415.035 077 2	−415.110 411 9	−415.052 987 6	−11.11	−11.24
4a→4b	−506.453 198 8	−506.402 874 6	−506.493 767 5	−506.444 080 9	−25.46	−25.86
5a→5b	−355.855 563 4	−355.813 124 2	−355.852 712	−355.810 664 6	1.79	1.54
6a→6b	−431.153 975 4	−431.106 935 4	−431.168 807 9	−431.123 108 5	−9.31	−10.15
7a→7b	−303.860 641 2	−303.783 452 6	−303.837 946 3	−303.760 425 1	14.24	14.45
8a→8b	−375.335 016 1	−375.254 069 7	−375.302 623 6	−375.221 288 6	20.33	20.57
9a→9b	−446.825 408 8	−446.740 853	−446.775 097 5	−446.690 106 6	31.57	31.84
10a→10b	−287.796 420 9	−287.709 436	−287.759 871 5	−287.672 434 6	22.94	23.22
11a→11b	−343.196 748 2	−343.096 872 5	−343.146 942 2	−343.046 184 6	31.25	31.81
12a→12b	−398.604 894 9	−398.492 472 4	−398.537 217 5	−398.422 496 3	42.47	43.91
13a→13b	−466.702 325 4	−466.630 031 9	−466.696 768	−466.624 835 3	3.49	3.26
14a→14b	−486.578 332 4	−486.517 217 1	−486.587 444 1	−486.526 612 4	−5.72	−5.90

Table S3. Absolute energies and free energies computed at the ωB97M-D4rev/def2-TZVPP level of the benzenoid tautomers investigated in the work. Energy differences in kcal mol^{-1}.

	tautomer *a*		tautomer *b*			
	E	G	E	G	ΔE	ΔG
phenol	−307.679 931 4	−307.603 645 9	−307.648 520 6	−307.574 185 7	19.71	18.49
resorcinol	−382.956 927 3	−382.877 808 6	−382.937 225 6	−382.860 006 1	12.36	11.17
phloroglucinol	−458.234 564 4	−458.152 495 4	−458.223 335 6	−458.143 737 7	7.05	5.50

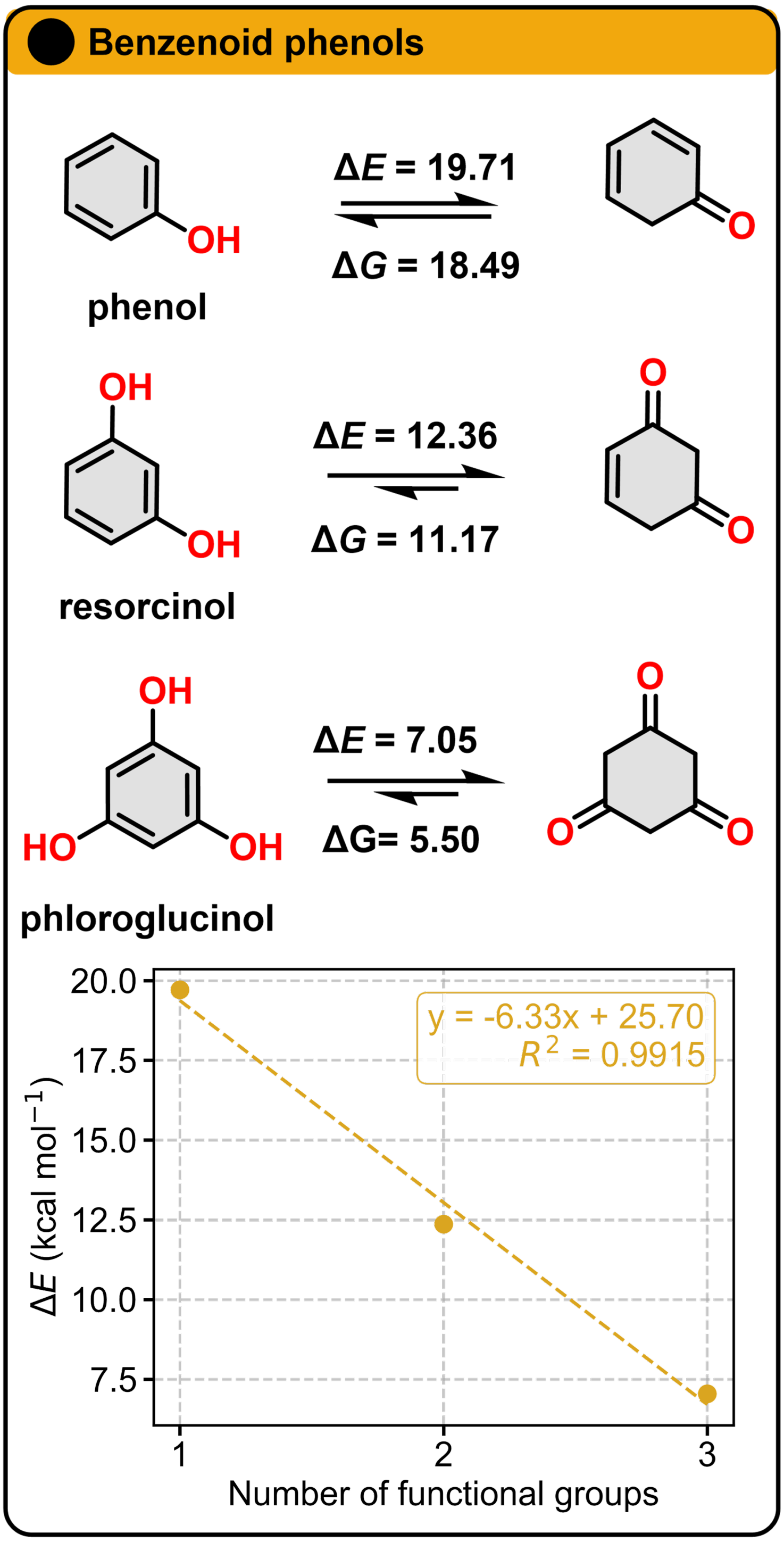

Figure S1. Relative energies (in kcal mol^{-1}) for phenol, resorcinol and phloroglucinol and their corresponding keto tautomers. The upper part shows calculated ΔE and ΔG with structures; the lower plots ΔE versus the count of exchanging groups. Energies (in kcal mol^{-1}) were obtained at the ωB97M-D4rev/def2-TZVPP level.

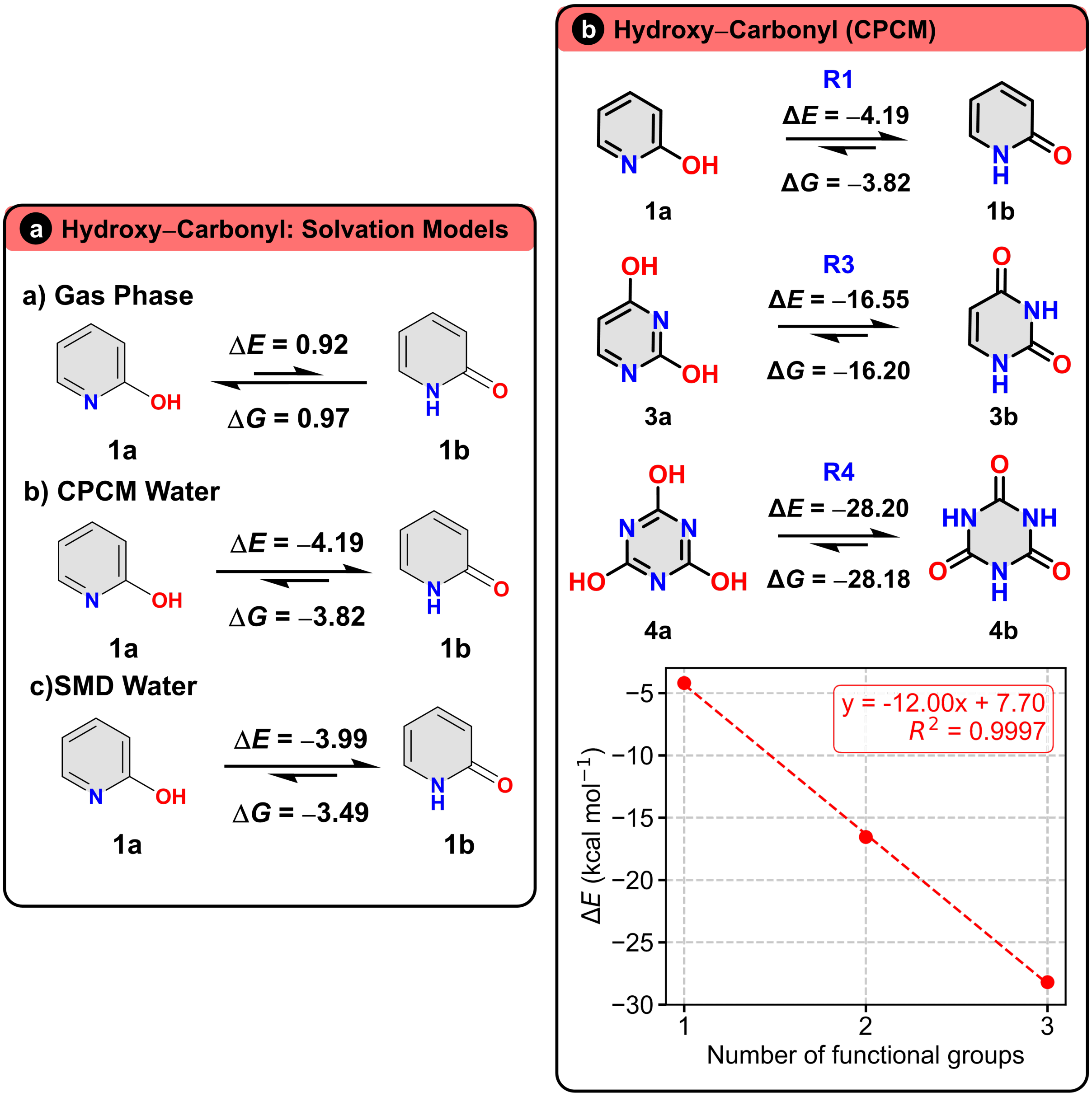

Figure S2. Effect of implicit solvation in the relative energies (in kcal mol^{-1}) for (a) 2-hydroxypyridine/2-pyridone (**1a**/**1b**) tautomeric equilibria using different solvation models. (b) For the hydroxy–carbonyl pairs **1a**/**1b**, **3a**/**3b**, **4a**/**4b**. Energies (in kcal mol^{-1}) were obtained at the ωB97M-D4rev/def2-TZVPP level.

S2 Quantum interference and the GPF-EP method

S2.1 Quantum interference in chemical systems

To familiarise the reader with a key part of the theoretical framework used in this work, we provide a concise account of quantum interference and its role in chemical bonding. Since the seminal work of Ruedenberg in 1962, interference has provided a rigorous way to analyse the density redistribution associated with covalent bond formation,[1] although this concept remains less common in the language of organic structure and reactivity.

In this context, interference refers to the density redistribution that arises from cross terms between non-orthogonal one-electron functions. The idea is closely related to Feynman's formulation of quantum-mechanical amplitudes: when an event can occur through alternative indistinguishable pathways, the corresponding probability amplitudes are added before the probability is obtained by taking the squared modulus.[2] The double-slit experiment provides the familiar physical illustration of this rule, where adding amplitudes rather than probabilities gives rise to interference fringes. In chemical bonding, an analogous interference term appears when orbital amplitudes are coherently combined.

For two interacting atomic orbitals, ϕ_1 and ϕ_2, a purely additive density picture would contain only the independent-orbital terms. We denote this classical-like, non-interfering reference density as P_{cl}:

$$P_{\mathrm{cl}} = \phi_1^2 + \phi_2^2 \tag{S1}$$

In the quantum-mechanical description, however, the coherent combination of the two orbital amplitudes gives a density containing an additional cross term:

$$P = [\phi_1 + \phi_2]^2 = \underbrace{\phi_1^2 + \phi_2^2}_{\text{independent-orbital}} + \underbrace{2\phi_1\phi_2}_{\text{interference}} \tag{S2}$$

The cross term $2\phi_1\phi_2$ is the interference contribution. In a bonding combination, this term redistributes electron density from the separated atomic regions toward the internuclear region, without changing the total electron count. Ruedenberg showed that this interference-driven density redistribution provides a quantum-mechanical basis for covalent bond formation.[1]

The generalised product function energy partitioning (GPF-EP) method[3,4] provides, for a given GPF wavefunction, an exact decomposition of the molecular electronic energy into quasi-classical and interference contributions rooted in Ruedenberg's interference analysis of chemical bonding.[1] The derivation below is self-contained: we start from the GPF wavefunction ansatz, establish the block structure of the reduced density matrices (RDMs), introduce the Ruedenberg density decomposition, and define each term in the partition $E[\mathrm{TOT}] = E[\mathrm{QC}] + E[\mathrm{INT}]$. Throughout, $\vec{r}$ denotes spatial coordinates (spin coordinates are integrated out wherever they do not affect the final expressions), Greek indices μ, ν label electron groups, and Latin indices j, k label non-orthogonal SCGVB orbitals within a group.

S2.2 The GPF wavefunction

A generalised product function (GPF)[5] partitions the N electrons of the system into G groups of N_μ electrons each ($\sum_\mu N_\mu = N$, $\mu = 1, \ldots, G$). The many-electron wavefunction is expressed as the inter-group antisymmetrised product of group functions:

$$\Psi_{\mathrm{GPF}}(\vec{x}_1, \ldots, \vec{x}_N) = \hat{\mathcal{A}}\Big\{\Psi^{(1)}(\vec{x}_1, \ldots, \vec{x}_{N_1})\, \Psi^{(2)}(\vec{x}_{N_1+1}, \ldots) \,\cdots\, \Psi^{(G)}(\ldots, \vec{x}_N)\Big\} \tag{S3}$$

where $\vec{x}_i = (\vec{r}_i, \sigma_i)$ combines spatial and spin coordinates of electron i, and $\hat{\mathcal{A}}$ is the inter-group antisymmetriser that enforces the Pauli principle across group boundaries. The group functions

must satisfy the *strong orthogonality* (SO) condition: for any $\mu \neq \nu$ and any fixed values of $\vec{x}_2, \vec{y}_2, \ldots$,

$$\int \Psi^{(\mu)*}(\vec{x}_1, \vec{x}_2, \ldots)\, \Psi^{(\nu)}(\vec{x}_1, \vec{y}_2, \ldots)\, \mathrm{d}\vec{x}_1 = 0 \tag{S4}$$

This means every orbital of group μ is orthogonal to every orbital of group ν. The SO condition does *not* impose orthogonality among orbitals within the same group: intra-group orbitals overlap freely, and it is precisely this intra-group overlap that produces the interference terms below.

Typically, valence electrons are placed in a group or groups, described by a SCGVB group wavefunction, and the remaining electrons (σ framework, lone pairs, core) form a second group treated at the Hartree–Fock (HF) level. Because HF orbitals are orthonormal, the HF group has no intra-group interference terms and contributes only through the quasi-classical and inter-group exchange terms (see Section S2.4).

S2.3 Reduced density matrices

All energy contributions of the GPF can be expressed through two objects: the first-order reduced density matrix (1-RDM) and the pair density (diagonal of the second-order reduced density matrix).

The **1-RDM** is:

$$\gamma_1(\vec{r}; \vec{r}') = N \sum_{\sigma} \int \Psi^*(\vec{r}, \sigma; \vec{x}_2, \ldots, \vec{x}_N)\, \Psi(\vec{r}', \sigma; \vec{x}_2, \ldots, \vec{x}_N)\, \mathrm{d}\vec{x}_2 \cdots \mathrm{d}\vec{x}_N \tag{S5}$$

Its diagonal is the one-electron density, $\rho(\vec{r}) = \gamma_1(\vec{r}; \vec{r})$, which integrates to N. The **pair density** is:

$$\pi(\vec{r}_1, \vec{r}_2) = N(N-1) \sum_{\sigma_1, \sigma_2} \int |\Psi(\vec{r}_1, \sigma_1; \vec{r}_2, \sigma_2; \vec{x}_3, \ldots)|^2\, \mathrm{d}\vec{x}_3 \cdots \mathrm{d}\vec{x}_N \tag{S6}$$

and integrates to $N(N-1)$. It gives the probability density of finding one electron at $\vec{r}_1$ and a different electron at $\vec{r}_2$ simultaneously.

Within group μ, the 1-RDM is represented in the basis of N_μ non-orthogonal, self-consistently optimised SCGVB orbitals $\{\phi_j^{(\mu)}(\vec{r})\}$:

$$\gamma_1^{(\mu)}(\vec{r}; \vec{r}') = \sum_{j=1}^{N_\mu} \sum_{k=1}^{N_\mu} \phi_j^{(\mu)}(\vec{r})\, p(j|k)\, \phi_k^{(\mu)}(\vec{r}') \tag{S7}$$

where $p(j|k)$ is element (j, k) of the first-order density matrix in the non-orthogonal orbital basis. Taking the diagonal gives the group density:

$$\rho^{(\mu)}(\vec{r}) = \underbrace{\sum_{j} \big[\phi_j^{(\mu)}(\vec{r})\big]^2 p(j|j)}_{\text{diagonal terms}} + \underbrace{\sum_{\substack{j,k \\ j \neq k}} \phi_j^{(\mu)}(\vec{r})\, \phi_k^{(\mu)}(\vec{r})\, p(j|k)}_{\text{cross terms}} \tag{S8}$$

Following Ruedenberg,[1] the cross product $\phi_j \phi_k$ $(j \neq k)$ is decomposed into an *interference function* $\langle j, k \rangle^{(\mu)}$ and a *compensation term* built from the individual squared densities:

$$\phi_j^{(\mu)}(\vec{r})\, \phi_k^{(\mu)}(\vec{r}) = \underbrace{\phi_j^{(\mu)} \phi_k^{(\mu)} - \frac{\xi(j,k)}{2} \Big[\big(\phi_j^{(\mu)}\big)^2 + \big(\phi_k^{(\mu)}\big)^2 \Big]}_{\langle j,k \rangle^{(\mu)}} + \frac{\xi(j,k)}{2} \Big[\big(\phi_j^{(\mu)}\big)^2 + \big(\phi_k^{(\mu)}\big)^2 \Big] \tag{S9}$$

where $\xi(j,k) = \langle \phi_j^{(\mu)} | \phi_k^{(\mu)} \rangle$ is the overlap integral between orbitals j and k within group μ. The interference function has a fundamental property: it integrates to zero,

$$\int \langle j,k \rangle^{(\mu)} \, \mathrm{d}\vec{r} = \xi(j,k) - \frac{\xi(j,k)}{2}(1+1) = 0 \tag{S10}$$

This is an important fact: orbital interference cannot create or destroy electron density. It only *redistributes* the existing density. Where $\langle j,k \rangle^{(\mu)} > 0$, constructive interference accumulates electron density (bonding region between the nuclei); where $\langle j,k \rangle^{(\mu)} < 0$, destructive interference depletes it.

Substituting Eq. S9 into Eq. S8 and collecting all terms involving only squared orbital densities, one obtains the *quasi-classical density* and the *interference density*. After this substitution, the coefficient multiplying each squared orbital density $\left[\phi_j^{(\mu)}(\vec{r})\right]^2$ becomes:

$$p(j|j) + \sum_{k \neq j} \xi(j,k) p(j|k) = [S\Gamma]_{jj} \tag{S11}$$

where $S_{jk} = \xi(j,k)$ is the overlap matrix and $\Gamma_{jk} = p(j|k)$ is the first-order density matrix in the non-orthogonal orbital basis. For the singly occupied spin-coupled orbitals used in the present SCGVB group, this quantity is equal to one for every orbital j. The quasi-classical density therefore reduces to the simple sum of squared orbital densities, while the remaining cross terms define the interference density. The density decomposition then reads:

$$\rho^{(\mu)}(\vec{r}) = \rho_{\mathrm{QC}}^{(\mu)}(\vec{r}) + \rho_{\mathrm{I}}^{(\mu)}(\vec{r}) \tag{S12}$$

with

$$\rho_{\mathrm{QC}}^{(\mu)}(\vec{r}) = \sum_{j=1}^{N_\mu} \left[\phi_j^{(\mu)}(\vec{r})\right]^2 \tag{S13}$$

$$\rho_{\mathrm{I}}^{(\mu)}(\vec{r}) = \sum_{\substack{j,k=1 \\ j \neq k}}^{N_\mu} \langle j,k \rangle^{(\mu)}(\vec{r}) \, p(j|k) \tag{S14}$$

$\rho_{\mathrm{QC}}^{(\mu)}$ (Eq. S13) is the sum of squared orbital densities — the density that would exist if each SCGVB orbital contributed independently, with no mutual overlap. It integrates to N_μ and is everywhere non-negative. $\rho_{\mathrm{I}}^{(\mu)}$ (Eq. S14) is the correction from orbital overlap. By Eq. S10 it integrates to zero: interference redistributes density without changing the electron count. Since Eq. S12 holds for every group, the *total* density also decomposes exactly:

$$\rho(\vec{r}) = \rho_{\mathrm{QC}}(\vec{r}) + \rho_{\mathrm{I}}(\vec{r}), \qquad \rho_{\mathrm{QC}} = \sum_\mu \rho_{\mathrm{QC}}^{(\mu)}, \quad \rho_{\mathrm{I}} = \sum_\mu \rho_{\mathrm{I}}^{(\mu)} \tag{S15}$$

S2.4 Energy partitioning

The molecular Hamiltonian (in atomic units) is:

$$\hat{H} = \sum_i \hat{h}(\vec{r}_i) + \frac{1}{2} \sum_{i \neq j} \frac{1}{r_{ij}} + V_{nn}, \qquad \hat{h}(\vec{r}) = -\tfrac{1}{2}\nabla^2 + \hat{v}_{ne}(\vec{r}) \tag{S16}$$

and the total energy is expressed exactly through the 1-RDM and pair density:

$$E[\mathrm{TOT}] = \int \gamma_1(\vec{r};\vec{r}')\, \hat{h}(\vec{r}) \Big|_{\vec{r}'=\vec{r}} \mathrm{d}\vec{r} + \frac{1}{2} \iint \frac{\pi(\vec{r}_1,\vec{r}_2)}{r_{12}} \, \mathrm{d}\vec{r}_1 \mathrm{d}\vec{r}_2 + V_{nn} \tag{S17}$$

We now classify every contribution to Eq. S17 according to whether the density factors involved are quasi-classical (QC) or interference (I).

The one-electron operator $\hat{h}$ is linear in the 1-RDM. Using the orbital-basis representation (Eq. S7), the contribution of group μ splits naturally into diagonal ($j = k$) and off-diagonal ($j \neq k$) terms:

$$\int \gamma_1^{(\mu)} \hat{h}\, \mathrm{d}\vec{r} = \underbrace{\sum_j p(j|j) \langle \phi_j^{(\mu)} | \hat{h} | \phi_j^{(\mu)} \rangle}_{h[\mathrm{QC}]^{(\mu)}} + \underbrace{\sum_{\substack{j,k \\ j \neq k}} p(j|k) \langle \phi_j^{(\mu)} | \hat{h} | \phi_k^{(\mu)} \rangle}_{h[\mathrm{I}]^{(\mu)}} \tag{S18}$$

$h[\mathrm{QC}]^{(\mu)}$ involves only the diagonal density-matrix elements and captures the kinetic plus nuclear-attraction energy of the individual squared orbital densities. $h[\mathrm{I}]^{(\mu)}$ involves only the off-diagonal elements and includes two parts: (a) $T[\mathrm{I}]^{(\mu)}$, the *kinetic interference energy*, computed from the off-diagonal 1-RDM using the kinetic operator $-\nabla^2/2$; and (b) $V_{en}[\mathrm{I}]^{(\mu)}$, the nuclear-attraction energy of the interference density. Although $\int \rho_{\mathrm{I}}^{(\mu)}\, \mathrm{d}\vec{r} = 0$, neither $T[\mathrm{I}]$ nor $V_{en}[\mathrm{I}]$ individually vanishes, and it is the *lowering* of $T[\mathrm{I}]$ associated with constructive interference between orbitals that is the quantum-mechanical origin of the covalent bond in Ruedenberg's picture.[1,3,6]

For the electron–electron repulsion between groups $\mu \neq \nu$, the inter-group pair density yields two distinct contributions. The Coulomb term $J^{(\mu\nu)}$ is:

$$J^{(\mu\nu)} = \iint \frac{\rho^{(\mu)}(\vec{r}_1)\, \rho^{(\nu)}(\vec{r}_2)}{r_{12}}\, \mathrm{d}\vec{r}_1 \mathrm{d}\vec{r}_2 \tag{S19}$$

The exchange term $K^{(\mu\nu)}$ originates from the inter-group antisymmetry of $\hat{\mathcal{A}}$:

$$K^{(\mu\nu)} = \iint \frac{\gamma_1^{(\mu)}(\vec{r}_1, \vec{r}_2)\, \gamma_1^{(\nu)}(\vec{r}_2, \vec{r}_1)}{r_{12}}\, \mathrm{d}\vec{r}_1 \mathrm{d}\vec{r}_2 \tag{S20}$$

This term is purely quantum-mechanical in origin (no classical analogue). It uses the *full* off-diagonal 1-RDM and it is collected entirely into $E[X]$.

The intra-group pair density $\pi^{(\mu)}(\vec{r}_1, \vec{r}_2)$ involves the full intra-group 2-RDM. When expanded in the orbital basis of group μ and the products of two orbital densities are classified using Eq. S9, the same three types of contributions arise: QC×QC (contributes to $E[\mathrm{REF}]$), QC×I (contributes to $E[I]$), and I×I (contributes to $E[II]$). For the complete orbital-basis expressions of the intra-group terms, see refs 3 and 4.

Collecting all contributions by the order of interference density factors involved gives the four-term partition:[3]

$$E[\mathrm{REF}] = \sum_\mu h[\mathrm{QC}]^{(\mu)} + \frac{1}{2} \sum_\mu J[\mathrm{QC}]^{(\mu\mu)} + \sum_{\mu<\nu} J[\mathrm{QC}]^{(\mu\nu)} + V_{nn} \tag{S21}$$

$$E[X] = -\sum_{\mu<\nu} K^{(\mu\nu)} \tag{S22}$$

$$E[I] = \sum_\mu h[\mathrm{I}]^{(\mu)} + \frac{1}{2} \sum_\mu J[\mathrm{QC}{\times}\mathrm{I}]^{(\mu\mu)} + \sum_{\mu<\nu} J[\mathrm{QC}{\times}\mathrm{I}]^{(\mu\nu)} \tag{S23}$$

$$E[II] = \frac{1}{2} \sum_\mu J[\mathrm{I}{\times}\mathrm{I}]^{(\mu\mu)} + \sum_{\mu<\nu} J[\mathrm{I}{\times}\mathrm{I}]^{(\mu\nu)} \tag{S24}$$

where $J[\mathrm{QC}]^{(\mu\mu)}/2$ is the intra-group quasi-classical Coulomb repulsion (the factor 1/2 prevents double-counting of electron pairs), and similarly for the I×I intra-group term.$E[II]$ involves no

one-electron contribution (there is no kinetic or nuclear-attraction term involving two interference densities simultaneously, because $\hat{h}$ is a one-electron operator linear in the 1-RDM).

The total energy is therefore:

$$E[\mathrm{TOT}] = E[\mathrm{REF}] + E[X] + E[I] + E[II] \tag{S25}$$

Defining the exchange-corrected quasi-classical energy and the total interference energy:

$$E[\mathrm{QC}] = E[\mathrm{REF}] + E[X] \tag{S26}$$

$$E[\mathrm{INT}] = E[I] + E[II] \tag{S27}$$

one obtains the two-term partition used in the main text:

$$\boxed{E[\mathrm{TOT}] = E[\mathrm{QC}] + E[\mathrm{INT}]} \tag{S28}$$

Definitions are as follows:

E[REF]. The energy the molecule would have if every SCGVB orbital contributed only its own squared density $[\phi_j^{(\mu)}]^2$, with no interference between different orbitals of the same group. It includes: the kinetic and nuclear-attraction energies of the individual squared orbital densities; all intra-group and inter-group Coulomb repulsions between quasi-classical densities; all energy contributions from the HF group (for which no interference analysis is carried out); and the nuclear repulsion V_{nn}. This is the "classical orbital" energy.

$E[X]$. The inter-group exchange correction arising from the antisymmetry $\hat{\mathcal{A}}$ of the GPF (Eq. S22). This term arises from enforcing antisymmetry between electron groups and is identically zero if only one SCGVB group is present. Together with E[REF], it forms the exchange-corrected quasi-classical energy E[QC].

$E[I]$. The first-order interference energy: all contributions in which *exactly one* density factor is an interference density. The one-electron part $h[\mathrm{I}]^{(\mu)} = T[\mathrm{I}]^{(\mu)} + V_{en}[\mathrm{I}]^{(\mu)}$ is the most physically significant component. The kinetic part T[I] is substantially *negative* in bonding regions. In Ruedenberg's analysis, this kinetic-energy lowering is a central signature of the quantum-mechanical origin of covalent binding.[1,6]

$E[II]$. The second-order interference energy: all two-electron contributions in which *both* density factors are interference densities (Eq. S24). This is a purely two-electron term ($E[II]$ has no one-electron component) and is generally smaller in magnitude than $E[I]$, but it is non-negligible when orbital overlaps are large, as in highly polarised bonds (see Table S4 for the numerical values in the present systems).

S2.5 Limiting case and relevance to tautomeric preferences

If all intra-group orbital overlaps vanish ($\xi(j,k) \to 0$ for every $j \neq k$ within every group), then $\langle j,k\rangle^{(\mu)} \to 0$ and $\rho_{\mathrm{I}}^{(\mu)} \to 0$ for all μ. In this limit, $E[\mathrm{INT}] \to 0$ and the total energy reduces to the quasi-classical value, $E[\mathrm{TOT}] \to E[\mathrm{QC}]$. Thus, within Ruedenberg's analysis, covalent bond formation requires interference-driven density redistribution; in the zero-overlap limit, the interference contribution vanishes and covalent stabilisation is lost.[1]

In the present work, E[QC] consistently favours the aromatic tautomers, reflecting the preference of the quasi-classical term for the more symmetric, delocalised arrangement of the ring p-like orbital densities. By contrast, E[INT] favours localisation into stronger two-centre, two-electron

(2c–2e) bonds, most notably C=O bonds. The tautomeric outcome is therefore determined by the competition between the quasi-classical preference for aromatic arrangements and the interference stabilisation associated with localised bond formation, as discussed in detail in the main text.

S3 SCGVB and GPF-EP data

Table S4. GPF-EP energy partitioning for pyridine, triazine, and selected tautomeric species, evaluated according to Eq. S25. Calculations at the SCGVB(6,6)/cc-pVDZ//ωB97M-D4rev/def2-TZVPP level. Energies in hartree.

	E[TOT]	E[REF]	E[X]	E[I]	E[II]
Pyridine	−246.777 559	−245.630 690	−0.971 704	−0.165 348	−0.009 817
Triazine	−278.781 322	−277.505 890	−1.083 357	−0.178 235	−0.013 840
**1a**	−321.653 994	−320.475 253	−0.996 062	−0.169 693	−0.012 986
**1b**	−321.655 567	−320.327 459	−1.102 897	−0.219 492	−0.005 718
**3a**	−412.535 995	−411.254 741	−1.083 567	−0.177 126	−0.020 561
**3b**	−412.568 658	−411.007 840	−1.258 723	−0.304 891	0.002 795
**5a**	−353.661 853	−352.347 888	−1.114 770	−0.180 169	−0.019 026
**5b**	−353.664 330	−352.205 734	−1.214 318	−0.236 789	−0.007 489
**6a**	−428.543 885	−427.191 347	−1.145 960	−0.180 804	−0.025 775
**6b**	−428.576 360	−426.950 585	−1.315 939	−0.312 205	0.002 370
**7a**	−301.818 751	−300.640 424	−0.998 234	−0.167 060	−0.013 032
**7b**	−301.799 027	−300.562 032	−1.027 028	−0.204 105	−0.005 863
**10a**	−285.815 047	−284.654 126	−0.984 461	−0.166 066	−0.010 395
**10b**	−285.777 990	−284.617 261	−0.961 521	−0.193 485	−0.005 723

Table S5. Interference energy analysis of selected tautomeric equilibria within the SCGVB/GPF-EP framework. Calculations at the SCGVB(6,6)/cc-pVDZ//ωB97M-D4rev/def2-TZVPP level. Energies in kcal mol^{-1}.

	E[TOT]	E[INT]	E[QC]
**1a**→**1b**	−0.99	−26.69	25.70
**3a**→**3b**	−20.50	−65.44	44.95
**5a**→**5b**	−1.55	−28.29	26.74
**6a**→**6b**	−20.38	−64.79	44.42
**7a**→**7b**	12.38	−18.75	31.12
**10a**→**10b**	26.04	−14.26	40.31

Before discussing the individual pairwise p-like interference contributions in detail, it is useful to visualise the overall interference pattern for representative tautomers. Fig. S3 shows selected pairwise interference energies as heat maps for the aromatic **a** forms and the corresponding localised **b** forms. The aromatic tautomers display six stabilising negative adjacent contributions around the ring, consistent with a delocalised 6c–6e π bond. By contrast, the **b** tautomers exhibit an alternating pattern of negative and positive adjacent contributions, with three dominant negative terms corresponding to three localised 2c–2e π bonds. In the carbonyl, imine, and methylidene cases, the exocyclic double-bond contribution corresponds to $P(1,2)$ in **1b**, $P(1,2)$ in **7b**, and $P(5,6)$ in **10b**, respectively. This representation anticipates the more detailed pairwise analysis given below.

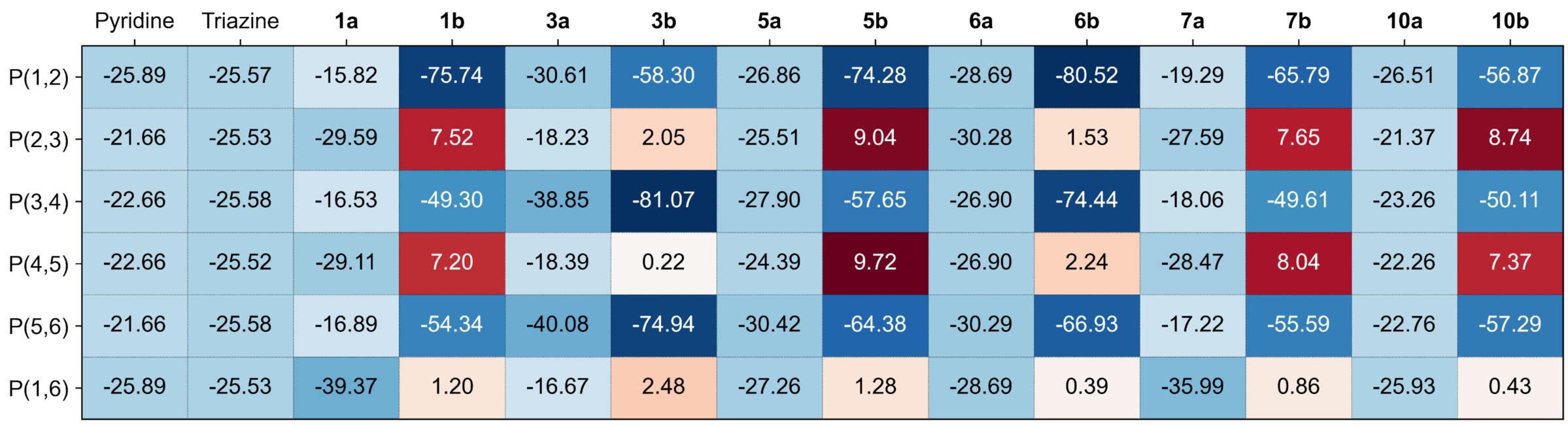

Figure S3. Selected pairwise *p*-like interference energies (kcal mol^{-1}) shown as heat maps for representative aromatic and non-aromatic tautomers. Aromatic **a** forms display six stabilising negative adjacent contributions, consistent with a delocalised 6c–6e π bond. In the corresponding **b** forms, the pattern becomes alternating, with three dominant negative contributions associated with three localised 2c–2e π bonds. The exocyclic double-bond contribution corresponds to $P(1,2)$ in **1b**, $P(1,2)$ in **7b**, and $P(5,6)$ in **10b**.

Table S6. Complete pairwise *p*-like orbital interference matrices for the structures analysed in Table S4 (adjacent and non-adjacent pairs). Aromatic tautomers display a transferable 6c–6e signature, whereas the corresponding **b** tautomers show localised patterns consistent with bond alternation. Energies in kcal mol^{-1}.

	Pyridine	Triazine	**1a**	**1b**	**3a**	**3b**	**5a**
$P(1,2)$	−25.89	−25.57	−15.82	−75.74	−30.61	−58.30	−26.86
$P(1,3)$	2.91	2.36	5.05	2.62	2.69	0.80	2.12
$P(2,3)$	3.67	5.17	5.52	1.30	7.36	−0.07	5.98
$P(1,4)$	2.91	2.36	8.38	−0.02	2.28	7.47	2.08
$P(2,4)$	−25.89	−25.53	−39.37	1.20	−16.67	2.48	−27.26
$P(3,4)$	−21.66	−25.53	−29.59	7.52	−18.23	2.05	−25.51
$P(1,5)$	3.94	6.29	3.43	4.56	8.75	0.36	5.97
$P(2,5)$	5.01	5.17	5.30	−0.22	5.31	6.56	5.97
$P(3,5)$	5.51	6.29	2.46	3.67	5.98	1.32	8.96
$P(4,5)$	−22.66	−25.58	−16.53	−49.30	−38.85	−81.07	−27.90
$P(1,6)$	3.78	2.36	3.98	4.79	2.00	1.50	2.19
$P(2,6)$	5.01	5.17	3.83	0.92	6.23	0.42	6.59
$P(3,6)$	−22.66	−25.52	−29.11	7.20	−18.39	0.22	−24.39
$P(4,6)$	3.94	6.29	2.88	8.12	11.09	−0.05	9.45
$P(5,6)$	−21.66	−25.58	−16.89	−54.34	−40.08	−74.94	−30.42

	**5b**	**6a**	**6b**	**7a**	**7b**	**10a**	**10b**
$P(1,2)$	−74.28	−28.69	−80.52	−19.29	−65.79	−26.51	−56.87
$P(1,3)$	2.49	1.91	0.20	2.79	3.01	2.86	7.79
$P(2,3)$	1.72	6.86	−0.05	3.98	1.45	3.83	1.38
$P(1,4)$	−0.03	1.91	−0.09	2.41	−0.07	2.81	2.31
$P(2,4)$	1.28	−28.69	0.39	−35.99	0.86	−25.93	0.43
$P(3,4)$	9.04	−30.28	1.53	−27.59	7.65	−21.37	8.74
$P(1,5)$	5.79	8.90	0.41	4.05	4.99	4.03	5.01
$P(2,5)$	0.10	7.84	0.83	5.39	−0.31	5.12	−0.38
$P(3,5)$	3.49	12.03	2.10	8.38	3.12	5.92	−0.10
$P(4,5)$	−57.65	−26.90	−74.44	−18.06	−49.61	−23.26	−50.11
$P(1,6)$	3.11	2.13	8.70	3.44	4.78	3.68	5.01
$P(2,6)$	1.23	7.84	7.97	5.85	1.10	5.19	1.61
$P(3,6)$	9.72	−26.90	2.24	−28.47	8.04	−22.26	7.37
$P(4,6)$	9.79	8.90	1.75	5.49	8.29	4.47	3.68
$P(5,6)$	−64.38	−30.29	−66.93	−17.22	−55.59	−22.76	−57.29

To complement the pairwise interference-energy analysis, Fig. S4 shows selected interference-

density plots for representative aromatic and non-aromatic tautomers. These real-space maps provide a direct visualisation of the density redistribution associated with orbital interference in the π space. In the aromatic **a** tautomers, the interference density is distributed around the ring in a manner consistent with a delocalised 6c–6e π bond. In the corresponding **b** tautomers, the pattern becomes localised into three main 2c–2e π-bonding regions, including the exocyclic carbonyl contribution when present. The particularly intense interference associated with the C=O bond is also evident, consistent with the strong local stabilisation discussed in the main text.

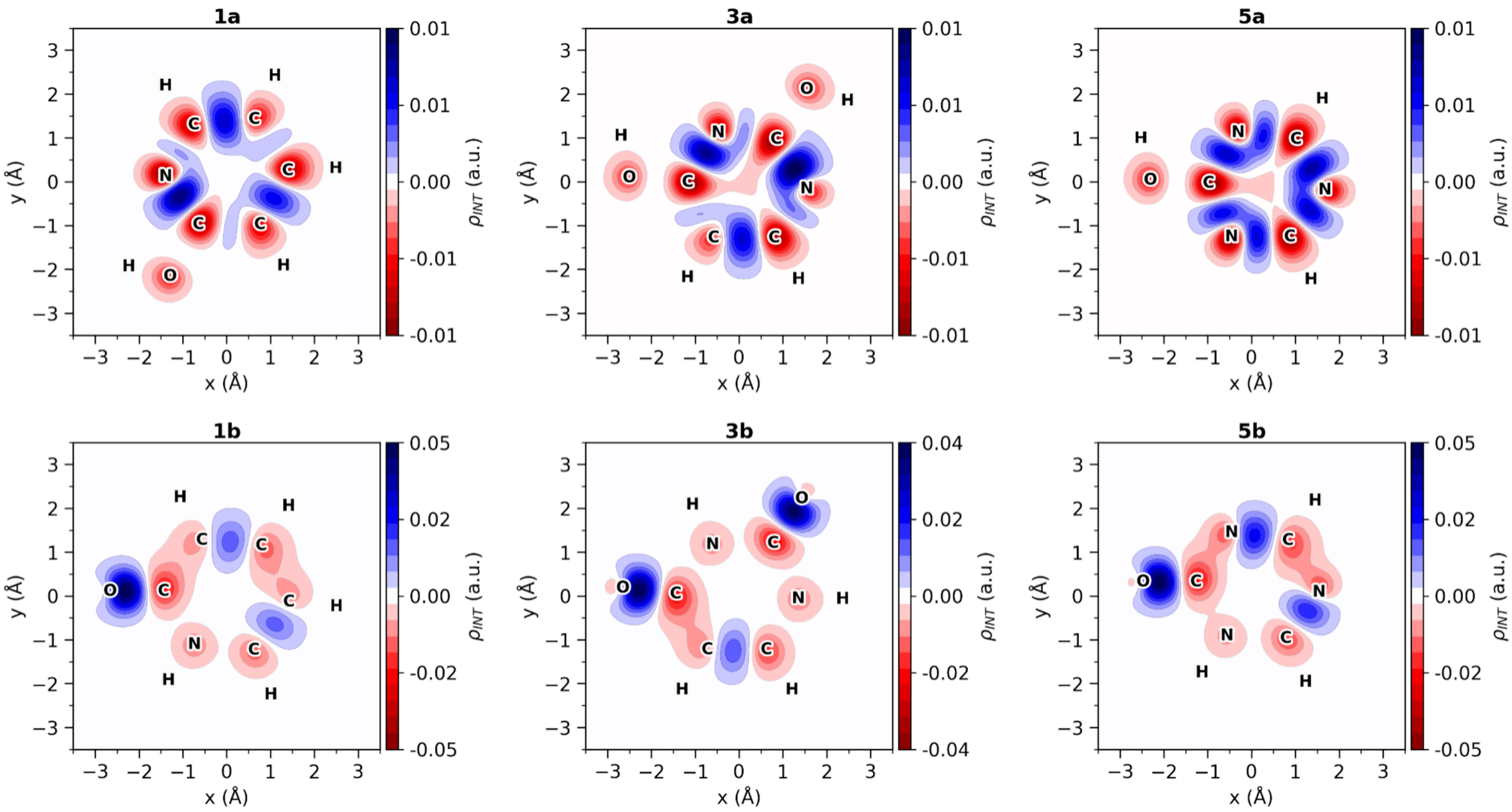

Figure S4. Selected interference-density plots, ρ_{INT}, for representative **a** and **b** tautomers in the π space. The aromatic **a** forms (top) show a delocalised pattern consistent with a 6c–6e π bond around the ring. In the corresponding **b** tautomers (bottom), the interference density is localised into three main 2c–2e π-bonding regions, consistent with bond localisation. The strong local contribution associated with the carbonyl π bond is clearly visible in the carbonylated tautomers. Density cuts were taken at $z = 0.3$ Å from the molecular plane. Blue and red indicate opposite signs of ρ_{INT}.

S4 NBO donor–acceptor analysis

Table S7. Selected dominant NBO donor–acceptor interactions used in the stereoelectronic analysis. Second-order perturbation energies, $E^{(2)}$, are given in kcal mol^{-1}. The resonance-type interactions compare amide-like, imine-like, methylidene, and benzenoid conjugation. The site-replacement rows compare the interaction available when the ring N–H unit in the lactam tautomer is replaced by a benzenoid CH_2 group.

System	Motif	Donor	Acceptor	$E^{(2)}$
Resonance-type interactions involving π^* *acceptors*				
**1b**	lactam-type carbonyl tautomer	n(N)	π^*(C=O)	72.26
**7b**	imine tautomer	n(N)	π^*(C=N)	63.32
**7b'**	higher-energy E imine isomer	n(N)	π^*(C=N)	63.50
**10b**	methylidene tautomer	n(N)	π^*(C=C)	55.36
Keto tautomer of phenol	benzenoid enone conjugation	π(C=C)	π^*(C=O)	29.13
Site-replacement comparison: N-lone-pair donation versus CH_2 *hyperconjugation*				
**1b**	lactam N-site donor	n(N)	π^*(C=O)	72.26
Keto tautomer of phenol	benzenoid CH_2 donor	σ(C–H)	π^*(C=O)	6.98
Keto tautomer of phenol	benzenoid CH_2 donor	σ(C–H)	π^*(C=O)	7.17
Keto tautomer of phenol	combined CH_2 donation	$2 \times \sigma$(C–H)	π^*(C=O)	14.15
Selected σ^**-acceptor interactions*				
**1b**	carbonylated N-heteroaromatic tautomer	n(O)	σ^*(C–N)	38.83
**7b**	lowest-energy imine tautomer	σ(N–H)	σ^*(C–N)	13.94
**7b'**	higher-energy E imine isomer	n(N)	σ^*(C–N)	21.83
**10b**	methylidene tautomer	σ(C–H)	σ^*(C–N)	9.30
Keto tautomer of phenol	benzenoid keto reference	n(O)	σ^*(C–C_{sp^3})	29.30

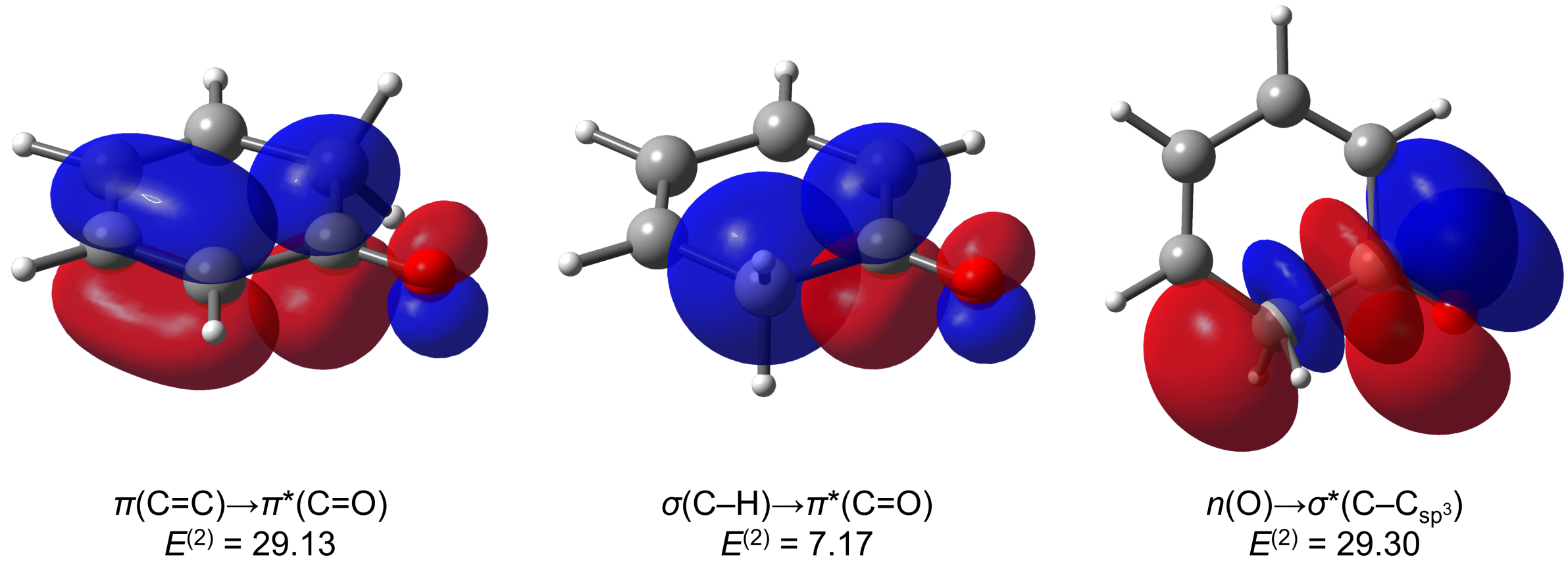

Figure S5. Selected dominant NBO donor–acceptor interactions in the keto tautomer of phenol, cyclohexa-2,4-dien-1-one. The left panel shows the main enone-like conjugative interaction, π(C=C) $\rightarrow$ π^*(C=O), with $E^{(2)} = 29.13$ kcal mol^{-1}. The middle panel shows one of the two CH_2-based hyperconjugative donations into the carbonyl antibonding orbital, σ(C–H) $\rightarrow$ π^*(C=O), with $E^{(2)} = 7.17$ kcal mol^{-1}; the second analogous σ(C–H) $\rightarrow$ π^*(C=O) interaction has $E^{(2)} = 6.98$ kcal mol^{-1}. The right panel shows the strongest anomeric-type interaction involving the carbonyl oxygen lone pair, n(O) $\rightarrow$ σ^*(C–C_{sp^3}), with $E^{(2)} = 29.30$ kcal mol^{-1}. These interactions provide all-carbon benzenoid reference points for comparison with the corresponding lactam-type n(N) $\rightarrow$ π^*(C=O) and n(O) $\rightarrow$ σ^*(C–N) interactions in **1b**.

Table S8. Complete NBO donor–acceptor interactions from the second-order perturbation analysis for the singly substituted tautomeric pairs. Only interactions above the printing threshold of 0.50 kcal mol^{-1} are listed. Second-order perturbation energies, $E^{(2)}$, are given in kcal mol^{-1}.

Compound	Donor no.	Donor NBO	Acceptor no.	Acceptor NBO	$E^{(2)}$
**Compound 1a**					
**1a**	8	LP (1) N6	26	BD*(1) C1–C2	14.27
**1a**	8	LP (1) N6	29	BD*(1) C1–O7	9.17
**1a**	8	LP (1) N6	35	BD*(1) C4–C5	10.93
**1a**	8	LP (1) N6	39	BD*(1) C5–H11	5.43
**1a**	8	LP (1) N6	40	BD*(1) O7–H12	0.79
**1a**	8	LP (1) N6	41	RY (1) C1	4.19
**1a**	8	LP (1) N6	145	RY (1) C5	3.23
**1a**	9	LP (1) O7	26	BD*(1) C1–C2	0.63
**1a**	9	LP (1) O7	27	BD*(1) C1–N6	7.94
**1a**	9	LP (1) O7	41	RY (1) C1	1.07
**1a**	9	LP (1) O7	42	RY (2) C1	2.71
**1a**	9	LP (1) O7	277	RY (3) H12	0.84
**1a**	10	LP (2) O7	28	BD*(2) C1–N6	53.95
**1a**	10	LP (2) O7	43	RY (3) C1	2.02
**1a**	10	LP (2) O7	276	RY (2) H12	1.71
**1a**	11	BD (1) C1–C2	27	BD*(1) C1–N6	0.70
**1a**	11	BD (1) C1–C2	30	BD*(1) C2–C3	0.84
**1a**	11	BD (1) C1–C2	34	BD*(1) C3–H9	2.90
**1a**	11	BD (1) C1–C2	40	BD*(1) O7–H12	1.74
**1a**	11	BD (1) C1–C2	93	RY (1) C3	1.30
**1a**	11	BD (1) C1–C2	94	RY (2) C3	1.08
**1a**	11	BD (1) C1–C2	171	RY (1) N6	1.15
**1a**	12	BD (1) C1–N6	26	BD*(1) C1–C2	1.29
**1a**	12	BD (1) C1–N6	32	BD*(1) C2–H8	1.47
**1a**	12	BD (1) C1–N6	39	BD*(1) C5–H11	2.40
**1a**	12	BD (1) C1–N6	146	RY (2) C5	2.77
**1a**	13	BD (2) C1–N6	31	BD*(2) C2–C3	17.36
**1a**	13	BD (2) C1–N6	36	BD*(2) C4–C5	44.63
**1a**	13	BD (2) C1–N6	69	RY (3) C2	0.59
**1a**	13	BD (2) C1–N6	147	RY (3) C5	2.47
**1a**	13	BD (2) C1–N6	197	RY (1) O7	1.03
**1a**	14	BD (1) C1–O7	30	BD*(1) C2–C3	1.19

Continued on next page

Table S8. Complete NBO donor–acceptor interactions (continued).

Compound	Donor no.	Donor NBO	Acceptor no.	Acceptor NBO	$E^{(2)}$
**1a**	14	BD (1) C1–O7	38	BD*(1) C5–N6	2.23
**1a**	14	BD (1) C1–O7	171	RY (1) N6	0.51
**1a**	15	BD (1) C2–C3	26	BD*(1) C1–C2	1.03
**1a**	15	BD (1) C2–C3	29	BD*(1) C1–O7	3.73
**1a**	15	BD (1) C2–C3	32	BD*(1) C2–H8	0.83
**1a**	15	BD (1) C2–C3	33	BD*(1) C3–C4	1.18
**1a**	15	BD (1) C2–C3	37	BD*(1) C4–H10	2.70
**1a**	15	BD (1) C2–C3	41	RY (1) C1	1.10
**1a**	15	BD (1) C2–C3	119	RY (1) C4	1.43
**1a**	15	BD (1) C2–C3	121	RY (3) C4	0.52
**1a**	16	BD (2) C2–C3	28	BD*(2) C1–N6	47.11
**1a**	16	BD (2) C2–C3	36	BD*(2) C4–C5	24.43
**1a**	16	BD (2) C2–C3	47	RY (7) C1	0.92
**1a**	16	BD (2) C2–C3	120	RY (2) C4	1.68
**1a**	17	BD (1) C2–H8	27	BD*(1) C1–N6	4.81
**1a**	17	BD (1) C2–H8	29	BD*(1) C1–O7	0.83
**1a**	17	BD (1) C2–H8	33	BD*(1) C3–C4	3.98
**1a**	17	BD (1) C2–H8	34	BD*(1) C3–H9	0.67
**1a**	17	BD (1) C2–H8	42	RY (2) C1	0.77
**1a**	17	BD (1) C2–H8	44	RY (4) C1	0.58
**1a**	17	BD (1) C2–H8	93	RY (1) C3	1.33
**1a**	18	BD (1) C3–C4	30	BD*(1) C2–C3	1.10
**1a**	18	BD (1) C3–C4	32	BD*(1) C2–H8	2.93
**1a**	18	BD (1) C3–C4	35	BD*(1) C4–C5	1.04
**1a**	18	BD (1) C3–C4	37	BD*(1) C4–H10	0.57
**1a**	18	BD (1) C3–C4	39	BD*(1) C5–H11	2.63
**1a**	18	BD (1) C3–C4	67	RY (1) C2	1.23
**1a**	18	BD (1) C3–C4	70	RY (4) C2	0.71
**1a**	18	BD (1) C3–C4	145	RY (1) C5	0.89
**1a**	18	BD (1) C3–C4	146	RY (2) C5	0.80
**1a**	19	BD (1) C3–H9	26	BD*(1) C1–C2	3.75
**1a**	19	BD (1) C3–H9	32	BD*(1) C2–H8	0.71
**1a**	19	BD (1) C3–H9	35	BD*(1) C4–C5	3.79
**1a**	19	BD (1) C3–H9	37	BD*(1) C4–H10	0.68

Continued on next page

Table S8. Complete NBO donor–acceptor interactions (continued).

Compound	Donor no.	Donor NBO	Acceptor no.	Acceptor NBO	$E^{(2)}$
**1a**	19	BD (1) C3–H9	67	RY (1) C2	1.84
**1a**	19	BD (1) C3–H9	71	RY (5) C2	0.59
**1a**	19	BD (1) C3–H9	119	RY (1) C4	1.66
**1a**	20	BD (1) C4–C5	33	BD*(1) C3–C4	1.03
**1a**	20	BD (1) C4–C5	34	BD*(1) C3–H9	2.82
**1a**	20	BD (1) C4–C5	37	BD*(1) C4–H10	0.58
**1a**	20	BD (1) C4–C5	93	RY (1) C3	1.39
**1a**	20	BD (1) C4–C5	94	RY (2) C3	0.57
**1a**	20	BD (1) C4–C5	171	RY (1) N6	0.87
**1a**	20	BD (1) C4–C5	172	RY (2) N6	0.61
**1a**	21	BD (2) C4–C5	28	BD*(2) C1–N6	24.98
**1a**	21	BD (2) C4–C5	31	BD*(2) C2–C3	42.32
**1a**	21	BD (2) C4–C5	36	BD*(2) C4–C5	0.63
**1a**	21	BD (2) C4–C5	96	RY (4) C3	1.06
**1a**	21	BD (2) C4–C5	173	RY (3) N6	1.56
**1a**	22	BD (1) C4–H10	30	BD*(1) C2–C3	3.84
**1a**	22	BD (1) C4–H10	34	BD*(1) C3–H9	0.64
**1a**	22	BD (1) C4–H10	38	BD*(1) C5–N6	4.75
**1a**	22	BD (1) C4–H10	39	BD*(1) C5–H11	0.67
**1a**	22	BD (1) C4–H10	93	RY (1) C3	1.38
**1a**	22	BD (1) C4–H10	146	RY (2) C5	1.38
**1a**	22	BD (1) C4–H10	150	RY (6) C5	0.52
**1a**	23	BD (1) C5–N6	29	BD*(1) C1–O7	4.74
**1a**	23	BD (1) C5–N6	35	BD*(1) C4–C5	0.67
**1a**	23	BD (1) C5–N6	37	BD*(1) C4–H10	1.80
**1a**	23	BD (1) C5–N6	42	RY (2) C1	0.79
**1a**	23	BD (1) C5–N6	44	RY (4) C1	1.15
**1a**	24	BD (1) C5–H11	27	BD*(1) C1–N6	4.66
**1a**	24	BD (1) C5–H11	33	BD*(1) C3–C4	4.09
**1a**	24	BD (1) C5–H11	37	BD*(1) C4–H10	0.68
**1a**	24	BD (1) C5–H11	119	RY (1) C4	1.54
**1a**	24	BD (1) C5–H11	171	RY (1) N6	0.86
**1a**	25	BD (1) O7–H12	26	BD*(1) C1–C2	6.01
**1a**	25	BD (1) O7–H12	27	BD*(1) C1–N6	0.97

Continued on next page

Table S8. Complete NBO donor–acceptor interactions (continued).

Compound	Donor no.	Donor NBO	Acceptor no.	Acceptor NBO	$E^{(2)}$
**1a**	25	BD (1) O7–H12	42	RY (2) C1	0.92
**Compound 1b**					
**1b**	8	LP (1) O1	28	BD*(1) C2–C3	3.15
**1b**	8	LP (1) O1	29	BD*(1) C2–N7	1.98
**1b**	8	LP (1) O1	67	RY (1) C2	13.47
**1b**	9	LP (2) O1	28	BD*(1) C2–C3	25.14
**1b**	9	LP (2) O1	29	BD*(1) C2–N7	38.83
**1b**	9	LP (2) O1	38	BD*(1) C6–N7	0.91
**1b**	9	LP (2) O1	68	RY (2) C2	2.49
**1b**	9	LP (2) O1	71	RY (5) C2	1.26
**1b**	10	LP (1) N7	27	BD*(2) O1–C2	72.26
**1b**	10	LP (1) N7	36	BD*(2) C5–C6	67.23
**1b**	10	LP (1) N7	69	RY (3) C2	0.85
**1b**	10	LP (1) N7	72	RY (6) C2	1.12
**1b**	10	LP (1) N7	173	RY (3) C6	3.18
**1b**	10	LP (1) N7	276	RY (2) H12	1.09
**1b**	11	BD (1) O1–C2	28	BD*(1) C2–C3	0.98
**1b**	11	BD (1) O1–C2	30	BD*(1) C3–C4	0.62
**1b**	11	BD (1) O1–C2	38	BD*(1) C6–N7	1.82
**1b**	12	BD (2) O1–C2	27	BD*(2) O1–C2	1.52
**1b**	12	BD (2) O1–C2	31	BD*(2) C3–C4	5.27
**1b**	13	BD (1) C2–C3	26	BD*(1) O1–C2	0.55
**1b**	13	BD (1) C2–C3	30	BD*(1) C3–C4	0.72
**1b**	13	BD (1) C2–C3	34	BD*(1) C4–H11	3.22
**1b**	13	BD (1) C2–C3	40	BD*(1) N7–H12	2.61
**1b**	13	BD (1) C2–C3	41	RY (1) O1	1.02
**1b**	13	BD (1) C2–C3	119	RY (1) C4	1.10
**1b**	13	BD (1) C2–C3	120	RY (2) C4	1.44
**1b**	13	BD (1) C2–C3	197	RY (1) N7	0.80
**1b**	14	BD (1) C2–N7	32	BD*(1) C3–H10	1.50
**1b**	14	BD (1) C2–N7	39	BD*(1) C6–H9	1.84
**1b**	14	BD (1) C2–N7	41	RY (1) O1	0.52
**1b**	14	BD (1) C2–N7	171	RY (1) C6	1.10
**1b**	14	BD (1) C2–N7	172	RY (2) C6	1.40

Continued on next page

Table S8. Complete NBO donor–acceptor interactions (continued).

Compound	Donor no.	Donor NBO	Acceptor no.	Acceptor NBO	$E^{(2)}$
**1b**	15	BD (1) C3–C4	26	BD*(1) O1–C2	2.30
**1b**	15	BD (1) C3–C4	28	BD*(1) C2–C3	0.54
**1b**	15	BD (1) C3–C4	32	BD*(1) C3–H10	0.91
**1b**	15	BD (1) C3–C4	33	BD*(1) C4–C5	1.12
**1b**	15	BD (1) C3–C4	34	BD*(1) C4–H11	0.61
**1b**	15	BD (1) C3–C4	37	BD*(1) C5–H8	2.26
**1b**	15	BD (1) C3–C4	68	RY (2) C2	0.61
**1b**	15	BD (1) C3–C4	145	RY (1) C5	1.34
**1b**	16	BD (2) C3–C4	27	BD*(2) O1–C2	36.14
**1b**	16	BD (2) C3–C4	36	BD*(2) C5–C6	18.56
**1b**	16	BD (2) C3–C4	146	RY (2) C5	1.60
**1b**	17	BD (1) C3–H10	29	BD*(1) C2–N7	4.38
**1b**	17	BD (1) C3–H10	30	BD*(1) C3–C4	0.57
**1b**	17	BD (1) C3–H10	33	BD*(1) C4–C5	5.18
**1b**	17	BD (1) C3–H10	34	BD*(1) C4–H11	0.92
**1b**	17	BD (1) C3–H10	70	RY (4) C2	0.83
**1b**	17	BD (1) C3–H10	119	RY (1) C4	1.57
**1b**	18	BD (1) C4–C5	30	BD*(1) C3–C4	0.94
**1b**	18	BD (1) C4–C5	32	BD*(1) C3–H10	3.08
**1b**	18	BD (1) C4–C5	35	BD*(1) C5–C6	1.00
**1b**	18	BD (1) C4–C5	39	BD*(1) C6–H9	3.40
**1b**	18	BD (1) C4–C5	93	RY (1) C3	1.17
**1b**	18	BD (1) C4–C5	94	RY (2) C3	1.03
**1b**	18	BD (1) C4–C5	171	RY (1) C6	0.91
**1b**	18	BD (1) C4–C5	172	RY (2) C6	0.71
**1b**	19	BD (1) C4–H11	28	BD*(1) C2–C3	5.06
**1b**	19	BD (1) C4–H11	32	BD*(1) C3–H10	0.89
**1b**	19	BD (1) C4–H11	35	BD*(1) C5–C6	3.54
**1b**	19	BD (1) C4–H11	37	BD*(1) C5–H8	0.64
**1b**	19	BD (1) C4–H11	93	RY (1) C3	2.24
**1b**	19	BD (1) C4–H11	99	RY (7) C3	0.52
**1b**	19	BD (1) C4–H11	145	RY (1) C5	1.52
**1b**	20	BD (1) C5–C6	33	BD*(1) C4–C5	1.15
**1b**	20	BD (1) C5–C6	34	BD*(1) C4–H11	2.17

Continued on next page

Table S8. Complete NBO donor–acceptor interactions (continued).

Compound	Donor no.	Donor NBO	Acceptor no.	Acceptor NBO	$E^{(2)}$
**1b**	20	BD (1) C5–C6	37	BD*(1) C5–H8	0.75
**1b**	20	BD (1) C5–C6	38	BD*(1) C6–N7	0.53
**1b**	20	BD (1) C5–C6	39	BD*(1) C6–H9	0.80
**1b**	20	BD (1) C5–C6	40	BD*(1) N7–H12	2.51
**1b**	20	BD (1) C5–C6	119	RY (1) C4	1.39
**1b**	20	BD (1) C5–C6	197	RY (1) N7	1.15
**1b**	21	BD (2) C5–C6	27	BD*(2) O1–C2	1.07
**1b**	21	BD (2) C5–C6	31	BD*(2) C3–C4	29.35
**1b**	21	BD (2) C5–C6	36	BD*(2) C5–C6	1.49
**1b**	21	BD (2) C5–C6	121	RY (3) C4	1.23
**1b**	21	BD (2) C5–C6	198	RY (2) N7	1.75
**1b**	22	BD (1) C5–H8	30	BD*(1) C3–C4	3.41
**1b**	22	BD (1) C5–H8	34	BD*(1) C4–H11	0.55
**1b**	22	BD (1) C5–H8	38	BD*(1) C6–N7	5.58
**1b**	22	BD (1) C5–H8	39	BD*(1) C6–H9	0.69
**1b**	22	BD (1) C5–H8	119	RY (1) C4	1.29
**1b**	22	BD (1) C5–H8	171	RY (1) C6	1.49
**1b**	23	BD (1) C6–N7	26	BD*(1) O1–C2	1.74
**1b**	23	BD (1) C6–N7	35	BD*(1) C5–C6	0.68
**1b**	23	BD (1) C6–N7	37	BD*(1) C5–H8	2.15
**1b**	23	BD (1) C6–N7	67	RY (1) C2	0.55
**1b**	23	BD (1) C6–N7	68	RY (2) C2	0.51
**1b**	23	BD (1) C6–N7	147	RY (3) C5	0.61
**1b**	24	BD (1) C6–H9	29	BD*(1) C2–N7	5.31
**1b**	24	BD (1) C6–H9	33	BD*(1) C4–C5	4.00
**1b**	24	BD (1) C6–H9	37	BD*(1) C5–H8	0.68
**1b**	24	BD (1) C6–H9	40	BD*(1) N7–H12	0.82
**1b**	24	BD (1) C6–H9	145	RY (1) C5	2.03
**1b**	24	BD (1) C6–H9	197	RY (1) N7	1.31
**1b**	25	BD (1) N7–H12	26	BD*(1) O1–C2	0.55
**1b**	25	BD (1) N7–H12	28	BD*(1) C2–C3	3.12
**1b**	25	BD (1) N7–H12	35	BD*(1) C5–C6	3.33
**1b**	25	BD (1) N7–H12	39	BD*(1) C6–H9	0.96
**1b**	25	BD (1) N7–H12	70	RY (4) C2	1.20

Continued on next page

Table S8. Complete NBO donor–acceptor interactions (continued).

Compound	Donor no.	Donor NBO	Acceptor no.	Acceptor NBO	$E^{(2)}$
**1b**	25	BD (1) N7–H12	171	RY (1) C6	1.53
**Compound 7a**					
**7a**	8	LP (1) N1	29	BD*(1) C2–C3	11.55
**7a**	8	LP (1) N1	31	BD*(1) C2–H7	5.84
**7a**	8	LP (1) N1	37	BD*(1) C5–C6	14.30
**7a**	8	LP (1) N1	39	BD*(1) C6–N11	5.75
**7a**	8	LP (1) N1	68	RY (1) C2	3.35
**7a**	8	LP (1) N1	172	RY (1) C6	4.03
**7a**	9	LP (1) N11	28	BD*(2) N1–C6	54.63
**7a**	9	LP (1) N11	174	RY (3) C6	1.13
**7a**	9	LP (1) N11	176	RY (5) C6	0.68
**7a**	9	LP (1) N11	277	RY (2) H12	1.02
**7a**	9	LP (1) N11	289	RY (1) H13	1.19
**7a**	10	BD (1) N1–C2	29	BD*(1) C2–C3	0.72
**7a**	10	BD (1) N1–C2	33	BD*(1) C3–H8	1.80
**7a**	10	BD (1) N1–C2	39	BD*(1) C6–N11	4.39
**7a**	10	BD (1) N1–C2	172	RY (1) C6	0.54
**7a**	10	BD (1) N1–C2	173	RY (2) C6	1.96
**7a**	11	BD (1) N1–C6	31	BD*(1) C2–H7	2.44
**7a**	11	BD (1) N1–C6	37	BD*(1) C5–C6	1.08
**7a**	11	BD (1) N1–C6	38	BD*(1) C5–H10	1.72
**7a**	11	BD (1) N1–C6	41	BD*(1) N11–H13	1.59
**7a**	11	BD (1) N1–C6	69	RY (2) C2	2.72
**7a**	12	BD (2) N1–C6	30	BD*(2) C2–C3	48.21
**7a**	12	BD (2) N1–C6	35	BD*(2) C4–C5	17.85
**7a**	12	BD (2) N1–C6	41	BD*(1) N11–H13	0.79
**7a**	12	BD (2) N1–C6	70	RY (3) C2	2.56
**7a**	12	BD (2) N1–C6	148	RY (3) C5	0.65
**7a**	12	BD (2) N1–C6	250	RY (1) N11	1.44
**7a**	13	BD (1) C2–C3	32	BD*(1) C3–C4	1.05
**7a**	13	BD (1) C2–C3	33	BD*(1) C3–H8	0.59
**7a**	13	BD (1) C2–C3	36	BD*(1) C4–H9	2.93
**7a**	13	BD (1) C2–C3	42	RY (1) N1	0.93
**7a**	13	BD (1) C2–C3	43	RY (2) N1	0.73

Continued on next page

Table S8. Complete NBO donor–acceptor interactions (continued).

Compound	Donor no.	Donor NBO	Acceptor no.	Acceptor NBO	$E^{(2)}$
**7a**	13	BD (1) C2–C3	120	RY (1) C4	1.34
**7a**	13	BD (1) C2–C3	121	RY (2) C4	0.55
**7a**	14	BD (2) C2–C3	28	BD*(2) N1–C6	24.23
**7a**	14	BD (2) C2–C3	30	BD*(2) C2–C3	0.74
**7a**	14	BD (2) C2–C3	35	BD*(2) C4–C5	44.28
**7a**	14	BD (2) C2–C3	44	RY (3) N1	1.30
**7a**	14	BD (2) C2–C3	123	RY (4) C4	1.00
**7a**	15	BD (1) C2–H7	27	BD*(1) N1–C6	5.11
**7a**	15	BD (1) C2–H7	32	BD*(1) C3–C4	4.13
**7a**	15	BD (1) C2–H7	33	BD*(1) C3–H8	0.69
**7a**	15	BD (1) C2–H7	42	RY (1) N1	0.89
**7a**	15	BD (1) C2–H7	94	RY (1) C3	1.51
**7a**	16	BD (1) C3–C4	29	BD*(1) C2–C3	1.06
**7a**	16	BD (1) C3–C4	31	BD*(1) C2–H7	2.62
**7a**	16	BD (1) C3–C4	33	BD*(1) C3–H8	0.59
**7a**	16	BD (1) C3–C4	34	BD*(1) C4–C5	1.17
**7a**	16	BD (1) C3–C4	38	BD*(1) C5–H10	3.02
**7a**	16	BD (1) C3–C4	68	RY (1) C2	0.93
**7a**	16	BD (1) C3–C4	69	RY (2) C2	0.74
**7a**	16	BD (1) C3–C4	146	RY (1) C5	1.31
**7a**	16	BD (1) C3–C4	149	RY (4) C5	0.81
**7a**	17	BD (1) C3–H8	26	BD*(1) N1–C2	4.68
**7a**	17	BD (1) C3–H8	31	BD*(1) C2–H7	0.66
**7a**	17	BD (1) C3–H8	34	BD*(1) C4–C5	3.85
**7a**	17	BD (1) C3–H8	36	BD*(1) C4–H9	0.63
**7a**	17	BD (1) C3–H8	69	RY (2) C2	1.38
**7a**	17	BD (1) C3–H8	73	RY (6) C2	0.51
**7a**	17	BD (1) C3–H8	120	RY (1) C4	1.38
**7a**	18	BD (1) C4–C5	32	BD*(1) C3–C4	1.23
**7a**	18	BD (1) C4–C5	33	BD*(1) C3–H8	2.74
**7a**	18	BD (1) C4–C5	37	BD*(1) C5–C6	1.30
**7a**	18	BD (1) C4–C5	38	BD*(1) C5–H10	0.81
**7a**	18	BD (1) C4–C5	39	BD*(1) C6–N11	3.64
**7a**	18	BD (1) C4–C5	94	RY (1) C3	1.37

Continued on next page

Table S8. Complete NBO donor–acceptor interactions (continued).

Compound	Donor no.	Donor NBO	Acceptor no.	Acceptor NBO	$E^{(2)}$
**7a**	18	BD (1) C4–C5	96	RY (3) C3	0.51
**7a**	18	BD (1) C4–C5	172	RY (1) C6	0.59
**7a**	18	BD (1) C4–C5	173	RY (2) C6	0.98
**7a**	19	BD (2) C4–C5	28	BD*(2) N1–C6	45.56
**7a**	19	BD (2) C4–C5	30	BD*(2) C2–C3	23.12
**7a**	19	BD (2) C4–C5	95	RY (2) C3	1.74
**7a**	19	BD (2) C4–C5	177	RY (6) C6	0.50
**7a**	20	BD (1) C4–H9	29	BD*(1) C2–C3	3.73
**7a**	20	BD (1) C4–H9	33	BD*(1) C3–H8	0.67
**7a**	20	BD (1) C4–H9	37	BD*(1) C5–C6	4.09
**7a**	20	BD (1) C4–H9	38	BD*(1) C5–H10	0.76
**7a**	20	BD (1) C4–H9	94	RY (1) C3	1.68
**7a**	20	BD (1) C4–H9	146	RY (1) C5	1.84
**7a**	21	BD (1) C5–C6	27	BD*(1) N1–C6	0.62
**7a**	21	BD (1) C5–C6	34	BD*(1) C4–C5	1.01
**7a**	21	BD (1) C5–C6	36	BD*(1) C4–H9	2.85
**7a**	21	BD (1) C5–C6	40	BD*(1) N11–H12	1.97
**7a**	21	BD (1) C5–C6	42	RY (1) N1	1.15
**7a**	21	BD (1) C5–C6	120	RY (1) C4	1.30
**7a**	21	BD (1) C5–C6	121	RY (2) C4	1.01
**7a**	21	BD (1) C5–C6	251	RY (2) N11	0.76
**7a**	22	BD (1) C5–H10	27	BD*(1) N1–C6	5.06
**7a**	22	BD (1) C5–H10	32	BD*(1) C3–C4	4.02
**7a**	22	BD (1) C5–H10	36	BD*(1) C4–H9	0.68
**7a**	22	BD (1) C5–H10	120	RY (1) C4	1.38
**7a**	22	BD (1) C5–H10	173	RY (2) C6	1.33
**7a**	23	BD (1) C6–N11	26	BD*(1) N1–C2	2.67
**7a**	23	BD (1) C6–N11	34	BD*(1) C4–C5	1.45
**7a**	23	BD (1) C6–N11	42	RY (1) N1	0.69
**7a**	24	BD (1) N11–H12	27	BD*(1) N1–C6	0.60
**7a**	24	BD (1) N11–H12	28	BD*(2) N1–C6	0.57
**7a**	24	BD (1) N11–H12	37	BD*(1) C5–C6	4.87
**7a**	24	BD (1) N11–H12	175	RY (4) C6	1.02
**7a**	25	BD (1) N11–H13	27	BD*(1) N1–C6	4.46

Continued on next page

Table S8. Complete NBO donor–acceptor interactions (continued).

Compound	Donor no.	Donor NBO	Acceptor no.	Acceptor NBO	$E^{(2)}$
**7a**	25	BD (1) N11–H13	28	BD*(2) N1–C6	1.51
**7a**	25	BD (1) N11–H13	172	RY (1) C6	0.82
**Compound 7b**					
**7b**	8	LP (1) N1	29	BD*(1) C2–C3	17.48
**7b**	8	LP (1) N1	30	BD*(1) C2–N7	5.42
**7b**	8	LP (1) N1	68	RY (1) C2	6.28
**7b**	8	LP (1) N1	277	RY (2) H12	0.75
**7b**	9	LP (1) N7	27	BD*(2) N1–C2	63.32
**7b**	9	LP (1) N7	37	BD*(2) C5–C6	69.70
**7b**	9	LP (1) N7	70	RY (3) C2	1.22
**7b**	9	LP (1) N7	73	RY (6) C2	0.80
**7b**	9	LP (1) N7	174	RY (3) C6	3.22
**7b**	9	LP (1) N7	290	RY (2) H13	1.10
**7b**	10	BD (1) N1–C2	29	BD*(1) C2–C3	1.53
**7b**	10	BD (1) N1–C2	30	BD*(1) C2–N7	0.51
**7b**	10	BD (1) N1–C2	31	BD*(1) C3–C4	0.76
**7b**	10	BD (1) N1–C2	39	BD*(1) C6–N7	2.62
**7b**	10	BD (1) N1–C2	198	RY (1) N7	0.78
**7b**	10	BD (1) N1–C2	200	RY (3) N7	0.51
**7b**	11	BD (2) N1–C2	27	BD*(2) N1–C2	1.63
**7b**	11	BD (2) N1–C2	32	BD*(2) C3–C4	8.37
**7b**	11	BD (2) N1–C2	96	RY (3) C3	0.59
**7b**	11	BD (2) N1–C2	199	RY (2) N7	1.07
**7b**	11	BD (2) N1–C2	276	RY (1) H12	0.73
**7b**	12	BD (1) N1–H12	29	BD*(1) C2–C3	0.93
**7b**	12	BD (1) N1–H12	30	BD*(1) C2–N7	13.94
**7b**	12	BD (1) N1–H12	68	RY (1) C2	0.82
**7b**	12	BD (1) N1–H12	71	RY (4) C2	0.90
**7b**	13	BD (1) C2–C3	26	BD*(1) N1–C2	0.89
**7b**	13	BD (1) C2–C3	31	BD*(1) C3–C4	0.94
**7b**	13	BD (1) C2–C3	35	BD*(1) C4–H10	3.15
**7b**	13	BD (1) C2–C3	41	BD*(1) N7–H13	2.79
**7b**	13	BD (1) C2–C3	42	RY (1) N1	1.37
**7b**	13	BD (1) C2–C3	120	RY (1) C4	1.12

Continued on next page

Table S8. Complete NBO donor–acceptor interactions (continued).

Compound	Donor no.	Donor NBO	Acceptor no.	Acceptor NBO	$E^{(2)}$
**7b**	13	BD (1) C2–C3	121	RY (2) C4	1.31
**7b**	13	BD (1) C2–C3	198	RY (1) N7	0.99
**7b**	14	BD (1) C2–N7	28	BD*(1) N1–H12	2.45
**7b**	14	BD (1) C2–N7	33	BD*(1) C3–H11	1.71
**7b**	14	BD (1) C2–N7	40	BD*(1) C6–H8	1.78
**7b**	14	BD (1) C2–N7	172	RY (1) C6	1.06
**7b**	14	BD (1) C2–N7	173	RY (2) C6	1.26
**7b**	15	BD (1) C3–C4	26	BD*(1) N1–C2	2.63
**7b**	15	BD (1) C3–C4	29	BD*(1) C2–C3	0.83
**7b**	15	BD (1) C3–C4	33	BD*(1) C3–H11	0.95
**7b**	15	BD (1) C3–C4	34	BD*(1) C4–C5	1.14
**7b**	15	BD (1) C3–C4	35	BD*(1) C4–H10	0.66
**7b**	15	BD (1) C3–C4	38	BD*(1) C5–H9	2.19
**7b**	15	BD (1) C3–C4	69	RY (2) C2	1.19
**7b**	15	BD (1) C3–C4	146	RY (1) C5	1.27
**7b**	16	BD (2) C3–C4	27	BD*(2) N1–C2	31.08
**7b**	16	BD (2) C3–C4	37	BD*(2) C5–C6	16.72
**7b**	16	BD (2) C3–C4	147	RY (2) C5	1.63
**7b**	17	BD (1) C3–H11	30	BD*(1) C2–N7	4.79
**7b**	17	BD (1) C3–H11	31	BD*(1) C3–C4	0.61
**7b**	17	BD (1) C3–H11	34	BD*(1) C4–C5	5.18
**7b**	17	BD (1) C3–H11	35	BD*(1) C4–H10	0.93
**7b**	17	BD (1) C3–H11	69	RY (2) C2	0.87
**7b**	17	BD (1) C3–H11	120	RY (1) C4	1.67
**7b**	18	BD (1) C4–C5	31	BD*(1) C3–C4	0.99
**7b**	18	BD (1) C4–C5	33	BD*(1) C3–H11	3.27
**7b**	18	BD (1) C4–C5	36	BD*(1) C5–C6	0.97
**7b**	18	BD (1) C4–C5	40	BD*(1) C6–H8	3.46
**7b**	18	BD (1) C4–C5	94	RY (1) C3	1.07
**7b**	18	BD (1) C4–C5	95	RY (2) C3	1.11
**7b**	18	BD (1) C4–C5	172	RY (1) C6	0.92
**7b**	18	BD (1) C4–C5	173	RY (2) C6	0.68
**7b**	19	BD (1) C4–H10	29	BD*(1) C2–C3	5.55
**7b**	19	BD (1) C4–H10	33	BD*(1) C3–H11	0.97

Continued on next page

Table S8. Complete NBO donor–acceptor interactions (continued).

Compound	Donor no.	Donor NBO	Acceptor no.	Acceptor NBO	$E^{(2)}$
**7b**	19	BD (1) C4–H10	36	BD*(1) C5–C6	3.39
**7b**	19	BD (1) C4–H10	38	BD*(1) C5–H9	0.61
**7b**	19	BD (1) C4–H10	94	RY (1) C3	2.19
**7b**	19	BD (1) C4–H10	100	RY (7) C3	0.53
**7b**	19	BD (1) C4–H10	146	RY (1) C5	1.50
**7b**	20	BD (1) C5–C6	34	BD*(1) C4–C5	1.13
**7b**	20	BD (1) C5–C6	35	BD*(1) C4–H10	2.17
**7b**	20	BD (1) C5–C6	38	BD*(1) C5–H9	0.80
**7b**	20	BD (1) C5–C6	39	BD*(1) C6–N7	0.57
**7b**	20	BD (1) C5–C6	40	BD*(1) C6–H8	0.80
**7b**	20	BD (1) C5–C6	41	BD*(1) N7–H13	2.47
**7b**	20	BD (1) C5–C6	120	RY (1) C4	1.35
**7b**	20	BD (1) C5–C6	198	RY (1) N7	1.01
**7b**	20	BD (1) C5–C6	200	RY (3) N7	0.73
**7b**	21	BD (2) C5–C6	27	BD*(2) N1–C2	0.73
**7b**	21	BD (2) C5–C6	32	BD*(2) C3–C4	28.77
**7b**	21	BD (2) C5–C6	37	BD*(2) C5–C6	1.75
**7b**	21	BD (2) C5–C6	122	RY (3) C4	1.32
**7b**	21	BD (2) C5–C6	199	RY (2) N7	1.67
**7b**	22	BD (1) C5–H9	31	BD*(1) C3–C4	3.34
**7b**	22	BD (1) C5–H9	35	BD*(1) C4–H10	0.53
**7b**	22	BD (1) C5–H9	39	BD*(1) C6–N7	5.61
**7b**	22	BD (1) C5–H9	40	BD*(1) C6–H8	0.69
**7b**	22	BD (1) C5–H9	120	RY (1) C4	1.27
**7b**	22	BD (1) C5–H9	172	RY (1) C6	1.49
**7b**	23	BD (1) C6–N7	26	BD*(1) N1–C2	1.74
**7b**	23	BD (1) C6–N7	36	BD*(1) C5–C6	0.71
**7b**	23	BD (1) C6–N7	38	BD*(1) C5–H9	2.14
**7b**	23	BD (1) C6–N7	68	RY (1) C2	1.56
**7b**	23	BD (1) C6–N7	148	RY (3) C5	0.65
**7b**	24	BD (1) C6–H8	30	BD*(1) C2–N7	5.64
**7b**	24	BD (1) C6–H8	34	BD*(1) C4–C5	4.08
**7b**	24	BD (1) C6–H8	38	BD*(1) C5–H9	0.70
**7b**	24	BD (1) C6–H8	41	BD*(1) N7–H13	0.86

Continued on next page

Table S8. Complete NBO donor–acceptor interactions (continued).

Compound	Donor no.	Donor NBO	Acceptor no.	Acceptor NBO	$E^{(2)}$
**7b**	24	BD (1) C6–H8	146	RY (1) C5	2.04
**7b**	24	BD (1) C6–H8	198	RY (1) N7	1.03
**7b**	24	BD (1) C6–H8	200	RY (3) N7	0.73
**7b**	25	BD (1) N7–H13	29	BD*(1) C2–C3	3.54
**7b**	25	BD (1) N7–H13	36	BD*(1) C5–C6	3.35
**7b**	25	BD (1) N7–H13	40	BD*(1) C6–H8	0.99
**7b**	25	BD (1) N7–H13	69	RY (2) C2	1.67
**7b**	25	BD (1) N7–H13	172	RY (1) C6	1.48
**Compound 10a**					
**10a**	8	LP (1) N1	29	BD*(1) C2–C3	12.19
**10a**	8	LP (1) N1	30	BD*(1) C2–H11	6.33
**10a**	8	LP (1) N1	36	BD*(1) C5–C6	13.80
**10a**	8	LP (1) N1	39	BD*(1) C6–C7	3.64
**10a**	8	LP (1) N1	40	BD*(1) C7–H12	0.54
**10a**	8	LP (1) N1	69	RY (1) C2	3.23
**10a**	8	LP (1) N1	173	RY (1) C6	4.05
**10a**	9	BD (1) N1–C2	29	BD*(1) C2–C3	0.71
**10a**	9	BD (1) N1–C2	33	BD*(1) C3–H10	1.75
**10a**	9	BD (1) N1–C2	39	BD*(1) C6–C7	4.06
**10a**	9	BD (1) N1–C2	174	RY (2) C6	3.29
**10a**	10	BD (2) N1–C2	32	BD*(2) C3–C4	22.36
**10a**	10	BD (2) N1–C2	37	BD*(2) C5–C6	44.37
**10a**	10	BD (2) N1–C2	96	RY (2) C3	0.68
**10a**	10	BD (2) N1–C2	175	RY (3) C6	1.11
**10a**	10	BD (2) N1–C2	177	RY (5) C6	0.62
**10a**	11	BD (1) N1–C6	30	BD*(1) C2–H11	2.56
**10a**	11	BD (1) N1–C6	36	BD*(1) C5–C6	0.76
**10a**	11	BD (1) N1–C6	38	BD*(1) C5–H8	2.01
**10a**	11	BD (1) N1–C6	40	BD*(1) C7–H12	0.91
**10a**	11	BD (1) N1–C6	70	RY (2) C2	2.73
**10a**	11	BD (1) N1–C6	199	RY (1) C7	0.52
**10a**	12	BD (1) C2–C3	31	BD*(1) C3–C4	1.01
**10a**	12	BD (1) C2–C3	35	BD*(1) C4–H9	2.98
**10a**	12	BD (1) C2–C3	43	RY (1) N1	1.02

Continued on next page

Table S8. Complete NBO donor–acceptor interactions (continued).

Compound	Donor no.	Donor NBO	Acceptor no.	Acceptor NBO	$E^{(2)}$
**10a**	12	BD (1) C2–C3	44	RY (2) N1	0.67
**10a**	12	BD (1) C2–C3	121	RY (1) C4	1.31
**10a**	12	BD (1) C2–C3	122	RY (2) C4	0.64
**10a**	13	BD (1) C2–H11	28	BD*(1) N1–C6	5.39
**10a**	13	BD (1) C2–H11	31	BD*(1) C3–C4	4.08
**10a**	13	BD (1) C2–H11	33	BD*(1) C3–H10	0.67
**10a**	13	BD (1) C2–H11	43	RY (1) N1	0.98
**10a**	13	BD (1) C2–H11	95	RY (1) C3	1.41
**10a**	14	BD (1) C3–C4	29	BD*(1) C2–C3	1.03
**10a**	14	BD (1) C3–C4	30	BD*(1) C2–H11	2.48
**10a**	14	BD (1) C3–C4	33	BD*(1) C3–H10	0.59
**10a**	14	BD (1) C3–C4	34	BD*(1) C4–C5	1.20
**10a**	14	BD (1) C3–C4	38	BD*(1) C5–H8	2.89
**10a**	14	BD (1) C3–C4	69	RY (1) C2	0.99
**10a**	14	BD (1) C3–C4	70	RY (2) C2	0.62
**10a**	14	BD (1) C3–C4	147	RY (1) C5	1.43
**10a**	14	BD (1) C3–C4	150	RY (4) C5	0.61
**10a**	15	BD (2) C3–C4	27	BD*(2) N1–C2	48.50
**10a**	15	BD (2) C3–C4	37	BD*(2) C5–C6	27.84
**10a**	15	BD (2) C3–C4	73	RY (5) C2	0.87
**10a**	15	BD (2) C3–C4	149	RY (3) C5	1.75
**10a**	16	BD (1) C3–H10	26	BD*(1) N1–C2	4.56
**10a**	16	BD (1) C3–H10	30	BD*(1) C2–H11	0.67
**10a**	16	BD (1) C3–H10	34	BD*(1) C4–C5	3.96
**10a**	16	BD (1) C3–H10	35	BD*(1) C4–H9	0.68
**10a**	16	BD (1) C3–H10	70	RY (2) C2	1.35
**10a**	16	BD (1) C3–H10	74	RY (6) C2	0.54
**10a**	16	BD (1) C3–H10	121	RY (1) C4	1.45
**10a**	17	BD (1) C4–C5	31	BD*(1) C3–C4	1.20
**10a**	17	BD (1) C4–C5	33	BD*(1) C3–H10	2.91
**10a**	17	BD (1) C4–C5	36	BD*(1) C5–C6	1.33
**10a**	17	BD (1) C4–C5	38	BD*(1) C5–H8	0.57
**10a**	17	BD (1) C4–C5	39	BD*(1) C6–C7	3.58
**10a**	17	BD (1) C4–C5	95	RY (1) C3	1.32

Continued on next page

Table S8. Complete NBO donor–acceptor interactions (continued).

Compound	Donor no.	Donor NBO	Acceptor no.	Acceptor NBO	$E^{(2)}$
**10a**	17	BD (1) C4–C5	97	RY (3) C3	0.52
**10a**	17	BD (1) C4–C5	173	RY (1) C6	1.27
**10a**	17	BD (1) C4–C5	174	RY (2) C6	0.59
**10a**	18	BD (1) C4–H9	29	BD*(1) C2–C3	3.88
**10a**	18	BD (1) C4–H9	33	BD*(1) C3–H10	0.70
**10a**	18	BD (1) C4–H9	36	BD*(1) C5–C6	4.08
**10a**	18	BD (1) C4–H9	38	BD*(1) C5–H8	0.73
**10a**	18	BD (1) C4–H9	95	RY (1) C3	1.67
**10a**	18	BD (1) C4–H9	147	RY (1) C5	1.72
**10a**	18	BD (1) C4–H9	151	RY (5) C5	0.57
**10a**	19	BD (1) C5–C6	34	BD*(1) C4–C5	1.09
**10a**	19	BD (1) C5–C6	35	BD*(1) C4–H9	2.83
**10a**	19	BD (1) C5–C6	43	RY (1) N1	1.11
**10a**	19	BD (1) C5–C6	121	RY (1) C4	1.33
**10a**	19	BD (1) C5–C6	122	RY (2) C4	0.89
**10a**	19	BD (1) C5–C6	199	RY (1) C7	0.73
**10a**	20	BD (2) C5–C6	27	BD*(2) N1–C2	31.05
**10a**	20	BD (2) C5–C6	32	BD*(2) C3–C4	44.04
**10a**	20	BD (2) C5–C6	41	BD*(1) C7–H13	3.26
**10a**	20	BD (2) C5–C6	42	BD*(1) C7–H14	3.21
**10a**	20	BD (2) C5–C6	45	RY (3) N1	1.49
**10a**	20	BD (2) C5–C6	124	RY (4) C4	1.66
**10a**	21	BD (1) C5–H8	28	BD*(1) N1–C6	5.53
**10a**	21	BD (1) C5–H8	31	BD*(1) C3–C4	3.98
**10a**	21	BD (1) C5–H8	35	BD*(1) C4–H9	0.70
**10a**	21	BD (1) C5–H8	121	RY (1) C4	1.35
**10a**	21	BD (1) C5–H8	174	RY (2) C6	1.44
**10a**	22	BD (1) C6–C7	26	BD*(1) N1–C2	3.86
**10a**	22	BD (1) C6–C7	34	BD*(1) C4–C5	2.63
**10a**	22	BD (1) C6–C7	43	RY (1) N1	1.24
**10a**	22	BD (1) C6–C7	147	RY (1) C5	0.95
**10a**	23	BD (1) C7–H12	28	BD*(1) N1–C6	4.93
**10a**	23	BD (1) C7–H12	176	RY (4) C6	0.50
**10a**	24	BD (1) C7–H13	36	BD*(1) C5–C6	2.05

Continued on next page

Table S8. Complete NBO donor–acceptor interactions (continued).

Compound	Donor no.	Donor NBO	Acceptor no.	Acceptor NBO	$E^{(2)}$
**10a**	24	BD (1) C7–H13	37	BD*(2) C5–C6	5.65
**10a**	24	BD (1) C7–H13	175	RY (3) C6	0.60
**10a**	25	BD (1) C7–H14	36	BD*(1) C5–C6	2.10
**10a**	25	BD (1) C7–H14	37	BD*(2) C5–C6	5.55
**10a**	25	BD (1) C7–H14	175	RY (3) C6	0.58
**Compound 10b**					
**10b**	8	LP (1) N1	30	BD*(2) C2–C3	65.90
**10b**	8	LP (1) N1	40	BD*(2) C6–C7	55.36
**10b**	8	LP (1) N1	70	RY (2) C2	3.25
**10b**	8	LP (1) N1	175	RY (3) C6	1.69
**10b**	8	LP (1) N1	178	RY (6) C6	0.68
**10b**	8	LP (1) N1	304	RY (2) H14	1.29
**10b**	9	BD (1) N1–C2	27	BD*(1) N1–C6	0.56
**10b**	9	BD (1) N1–C2	29	BD*(1) C2–C3	0.72
**10b**	9	BD (1) N1–C2	33	BD*(1) C3–H10	2.16
**10b**	9	BD (1) N1–C2	39	BD*(1) C6–C7	2.17
**10b**	9	BD (1) N1–C2	97	RY (3) C3	0.72
**10b**	9	BD (1) N1–C2	173	RY (1) C6	1.52
**10b**	9	BD (1) N1–C2	174	RY (2) C6	0.69
**10b**	10	BD (1) N1–C6	26	BD*(1) N1–C2	0.52
**10b**	10	BD (1) N1–C6	31	BD*(1) C2–H11	1.69
**10b**	10	BD (1) N1–C6	38	BD*(1) C5–H8	1.78
**10b**	10	BD (1) N1–C6	39	BD*(1) C6–C7	0.68
**10b**	10	BD (1) N1–C6	41	BD*(1) C7–H12	1.77
**10b**	10	BD (1) N1–C6	69	RY (1) C2	1.03
**10b**	10	BD (1) N1–C6	71	RY (3) C2	0.99
**10b**	10	BD (1) N1–C6	201	RY (3) C7	0.60
**10b**	11	BD (1) N1–H14	29	BD*(1) C2–C3	3.34
**10b**	11	BD (1) N1–H14	31	BD*(1) C2–H11	0.94
**10b**	11	BD (1) N1–H14	37	BD*(1) C5–C6	3.82
**10b**	11	BD (1) N1–H14	69	RY (1) C2	1.44
**10b**	11	BD (1) N1–H14	173	RY (1) C6	2.05
**10b**	12	BD (1) C2–C3	26	BD*(1) N1–C2	0.60
**10b**	12	BD (1) C2–C3	28	BD*(1) N1–H14	2.28

Continued on next page

Table S8. Complete NBO donor–acceptor interactions (continued).

Compound	Donor no.	Donor NBO	Acceptor no.	Acceptor NBO	$E^{(2)}$
**10b**	12	BD (1) C2–C3	31	BD*(1) C2–H11	0.86
**10b**	12	BD (1) C2–C3	32	BD*(1) C3–C4	1.12
**10b**	12	BD (1) C2–C3	33	BD*(1) C3–H10	0.84
**10b**	12	BD (1) C2–C3	36	BD*(1) C4–H9	2.08
**10b**	12	BD (1) C2–C3	43	RY (1) N1	1.20
**10b**	12	BD (1) C2–C3	121	RY (1) C4	1.38
**10b**	13	BD (2) C2–C3	30	BD*(2) C2–C3	1.66
**10b**	13	BD (2) C2–C3	35	BD*(2) C4–C5	24.73
**10b**	13	BD (2) C2–C3	40	BD*(2) C6–C7	0.54
**10b**	13	BD (2) C2–C3	44	RY (2) N1	1.62
**10b**	13	BD (2) C2–C3	123	RY (3) C4	1.46
**10b**	14	BD (1) C2–H11	27	BD*(1) N1–C6	5.87
**10b**	14	BD (1) C2–H11	28	BD*(1) N1–H14	0.93
**10b**	14	BD (1) C2–H11	32	BD*(1) C3–C4	4.19
**10b**	14	BD (1) C2–H11	33	BD*(1) C3–H10	0.72
**10b**	14	BD (1) C2–H11	43	RY (1) N1	1.18
**10b**	14	BD (1) C2–H11	95	RY (1) C3	2.07
**10b**	15	BD (1) C3–C4	29	BD*(1) C2–C3	0.94
**10b**	15	BD (1) C3–C4	31	BD*(1) C2–H11	3.63
**10b**	15	BD (1) C3–C4	34	BD*(1) C4–C5	1.02
**10b**	15	BD (1) C3–C4	38	BD*(1) C5–H8	3.38
**10b**	15	BD (1) C3–C4	69	RY (1) C2	0.85
**10b**	15	BD (1) C3–C4	71	RY (3) C2	0.75
**10b**	15	BD (1) C3–C4	147	RY (1) C5	1.17
**10b**	15	BD (1) C3–C4	148	RY (2) C5	1.09
**10b**	16	BD (1) C3–H10	26	BD*(1) N1–C2	5.88
**10b**	16	BD (1) C3–H10	29	BD*(1) C2–C3	0.51
**10b**	16	BD (1) C3–H10	31	BD*(1) C2–H11	0.71
**10b**	16	BD (1) C3–H10	34	BD*(1) C4–C5	3.17
**10b**	16	BD (1) C3–H10	69	RY (1) C2	1.52
**10b**	16	BD (1) C3–H10	121	RY (1) C4	1.27
**10b**	17	BD (1) C4–C5	32	BD*(1) C3–C4	1.15
**10b**	17	BD (1) C4–C5	33	BD*(1) C3–H10	2.12
**10b**	17	BD (1) C4–C5	36	BD*(1) C4–H9	0.74

Continued on next page

Table S8. Complete NBO donor–acceptor interactions (continued).

Compound	Donor no.	Donor NBO	Acceptor no.	Acceptor NBO	$E^{(2)}$
**10b**	17	BD (1) C4–C5	37	BD*(1) C5–C6	1.01
**10b**	17	BD (1) C4–C5	38	BD*(1) C5–H8	0.94
**10b**	17	BD (1) C4–C5	39	BD*(1) C6–C7	2.44
**10b**	17	BD (1) C4–C5	95	RY (1) C3	1.14
**10b**	17	BD (1) C4–C5	173	RY (1) C6	1.50
**10b**	17	BD (1) C4–C5	174	RY (2) C6	0.58
**10b**	18	BD (2) C4–C5	30	BD*(2) C2–C3	16.39
**10b**	18	BD (2) C4–C5	40	BD*(2) C6–C7	25.79
**10b**	18	BD (2) C4–C5	96	RY (2) C3	1.66
**10b**	18	BD (2) C4–C5	175	RY (3) C6	0.59
**10b**	19	BD (1) C4–H9	29	BD*(1) C2–C3	3.25
**10b**	19	BD (1) C4–H9	33	BD*(1) C3–H10	0.56
**10b**	19	BD (1) C4–H9	37	BD*(1) C5–C6	5.90
**10b**	19	BD (1) C4–H9	38	BD*(1) C5–H8	1.00
**10b**	19	BD (1) C4–H9	95	RY (1) C3	1.41
**10b**	19	BD (1) C4–H9	147	RY (1) C5	2.02
**10b**	19	BD (1) C4–H9	150	RY (4) C5	0.57
**10b**	20	BD (1) C5–C6	28	BD*(1) N1–H14	3.17
**10b**	20	BD (1) C5–C6	34	BD*(1) C4–C5	0.93
**10b**	20	BD (1) C5–C6	36	BD*(1) C4–H9	3.21
**10b**	20	BD (1) C5–C6	39	BD*(1) C6–C7	1.59
**10b**	20	BD (1) C5–C6	42	BD*(1) C7–H13	2.60
**10b**	20	BD (1) C5–C6	43	RY (1) N1	0.94
**10b**	20	BD (1) C5–C6	121	RY (1) C4	1.07
**10b**	20	BD (1) C5–C6	122	RY (2) C4	1.19
**10b**	20	BD (1) C5–C6	200	RY (2) C7	1.57
**10b**	21	BD (1) C5–H8	27	BD*(1) N1–C6	5.24
**10b**	21	BD (1) C5–H8	32	BD*(1) C3–C4	5.32
**10b**	21	BD (1) C5–H8	34	BD*(1) C4–C5	0.56
**10b**	21	BD (1) C5–H8	36	BD*(1) C4–H9	1.01
**10b**	21	BD (1) C5–H8	121	RY (1) C4	1.66
**10b**	21	BD (1) C5–H8	173	RY (1) C6	1.38
**10b**	22	BD (1) C6–C7	26	BD*(1) N1–C2	3.13
**10b**	22	BD (1) C6–C7	27	BD*(1) N1–C6	0.82

Continued on next page

Table S8. Complete NBO donor–acceptor interactions (continued).

Compound	Donor no.	Donor NBO	Acceptor no.	Acceptor NBO	$E^{(2)}$
**10b**	22	BD (1) C6–C7	34	BD*(1) C4–C5	1.36
**10b**	22	BD (1) C6–C7	37	BD*(1) C5–C6	1.85
**10b**	22	BD (1) C6–C7	41	BD*(1) C7–H12	0.59
**10b**	22	BD (1) C6–C7	42	BD*(1) C7–H13	0.54
**10b**	22	BD (1) C6–C7	43	RY (1) N1	1.19
**10b**	22	BD (1) C6–C7	45	RY (3) N1	0.54
**10b**	22	BD (1) C6–C7	147	RY (1) C5	1.11
**10b**	23	BD (2) C6–C7	35	BD*(2) C4–C5	13.63
**10b**	23	BD (2) C6–C7	40	BD*(2) C6–C7	1.48
**10b**	23	BD (2) C6–C7	44	RY (2) N1	1.15
**10b**	23	BD (2) C6–C7	149	RY (3) C5	1.07
**10b**	24	BD (1) C7–H12	27	BD*(1) N1–C6	9.30
**10b**	24	BD (1) C7–H12	39	BD*(1) C6–C7	0.66
**10b**	24	BD (1) C7–H12	174	RY (2) C6	1.59
**10b**	25	BD (1) C7–H13	27	BD*(1) N1–C6	0.93
**10b**	25	BD (1) C7–H13	37	BD*(1) C5–C6	7.38
**10b**	25	BD (1) C7–H13	174	RY (2) C6	1.62

S5 DFT validation

The benchmarking of the chosen level of theory was carried out using **R1** as a model system, due to the known challenges discussed in the main text. A geometry optimised at the PBE0/def2-TZVP level was used as a neutral reference. Based on this structure, energy differences were computed for several functionals with the def2-TZVP basis set and compared to the values predicted by DLPNO-CCSD(T) with CBS extrapolation (aug-cc-pVTZ/aug-cc-pVQZ).

Fig. S6a shows that most range-separated functionals achieve chemical accuracy, with ωB97M-D4rev standing out as the best performer relative to the DLPNO-CCSD(T) reference. Following the selection of the functional, a similar procedure was applied to determine the optimal basis set. The results obtained with ωB97M-D4rev using different basis sets were compared to the DLPNO-CCSD(T)/CBS reference. Fig. S6b indicates that all tested basis sets perform reasonably well. Among them, def2-TZVPP was selected as the best compromise between computational cost and accuracy.

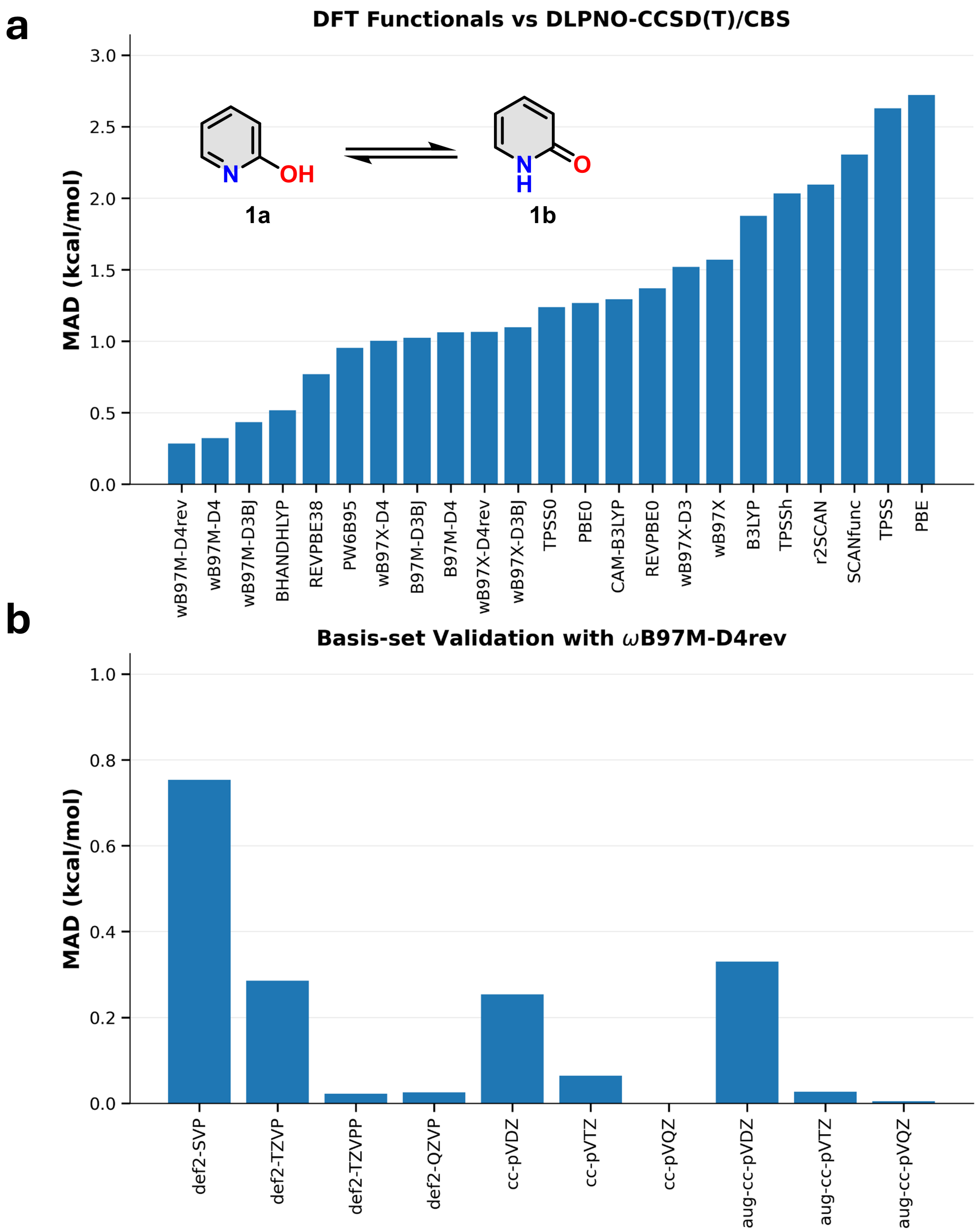

Figure S6. Mean absolute deviation with DLPNO-CCSD(T)/CBS as reference for (a) different DFT functionals with def2-TZVP basis set and (b) different basis sets using the ωB97M-D4rev functional.

S6 VB2000 example

```
 $CONTRL ICHARG=0 MULT=1 SCFTYP=RHF VBTYP=VB2000 ISPHER=1 MAXIT=200 $END
 $SYSTEM MEMORY=100000000 $END
 $BASIS GBASIS=CCD $END
 $DATA
Benzene SC
C1
CARBON          6.0     -1.207519644        -0.697161791         0.000000000
CARBON          6.0     -1.207519644         0.697161791         0.000000000
CARBON          6.0      0.000000000         1.394323583         0.000000000
CARBON          6.0      1.207519644         0.697161791         0.000000000
CARBON          6.0      1.207519644        -0.697161791         0.000000000
CARBON          6.0      0.000000000        -1.394323583         0.000000000
HYDROGEN        1.0     -2.153489568        -1.243317782         0.000000000
HYDROGEN        1.0     -2.153489568         1.243317782         0.000000000
HYDROGEN        1.0      0.000000000         2.486635563         0.000000000
HYDROGEN        1.0      2.153489568         1.243317782         0.000000000
HYDROGEN        1.0      2.153489568        -1.243317782         0.000000000
HYDROGEN        1.0      0.000000000        -2.486635563         0.000000000
$END
 $VB2000
#! VB(6)/cc-pVDZ

Benzene 2 GROUPS (1 HF + 1 Spin-Coupled(6,6) Group)

 $02PIVBO
 6
 1, 2, 3, 4, 5, 6

 $02VBSTR
 5
 1 2 3 4 5 6
 2 3 4 5 6 1
 1 4 2 3 5 6
 2 5 3 4 6 1
 3 6 4 5 1 2

 $NOTROT
 1
 1,2

 $MACROITER
 200

 $GPFEP
 partition.txt dmatrix.txt

 $MOLDEN

 $END
```

Figure S7. Example input file for benzene using SCGVB(6,6)/cc-pVDZ with the Rumer basis for the spin-coupling eigenfunctions. The input calls the GPF-EP subroutine used to compute the energy-partition terms. Further details are available in the VB2000 manual.

S7 DFT optimised cartesian coordinates and vibrational frequencies

Table S9. Optimized cartesian coordinates (Å) of all studied molecules at the ωB97M-D4rev/Def2-TZVPP level and their corresponding frequencies (cm^{-1}).

Pyridine

Atom	x	y	z
N	0.00000	0.00000	-1.36993
C	-1.13560	0.00000	-0.67654
C	-1.19054	0.00000	0.70963
C	0.00000	0.00000	1.41754
C	1.19054	0.00000	0.70963
C	1.13560	0.00000	-0.67654
H	-2.04876	0.00000	-1.25894
H	-2.14478	0.00000	1.21542
H	0.00000	0.00000	2.49843
H	2.14478	0.00000	1.21542
H	2.04876	0.00000	-1.25895

Frequencies (cm^{-1}):	388.57	420.66	612.62	668.31	723.15
	773.43	909.61	977.52	1021.69	1028
	1038.12	1060.5	1089.71	1102.73	1160.8
	1249.44	1264.44	1388.64	1486.24	1529.3
	1656.65	1663.71	3175.36	3177.7	3199.5
	3217.8	3225.25			

Triazine

Atom	x	y	z
N	-0.23010	-1.19305	0.00000
C	1.09376	-1.08952	0.00000
N	1.78492	0.04433	0.00000
C	1.03326	1.13901	0.00000
N	-0.29427	1.17066	0.00000
C	-0.86650	-0.02759	0.00000
H	-1.94916	-0.05703	0.00000
H	1.54914	2.09133	0.00000
H	1.66052	-2.01246	0.00000

Frequencies (cm^{-1}):	357.81	358.32	692.16	692.26	758.39
	969.26	1027.42	1063.73	1063.97	1150.28
	1166.76	1216.04	1216.08	1404.82	1458.82
	1458.9	1641.5	1641.55	3188.99	3189.18
	3194.07				

1a

Atom	x	y	z
C	0.00584	-0.00376	-0.00013
C	1.40135	0.00015	-0.00010
C	2.03078	1.22480	0.00002
C	1.26773	2.39041	0.00012
C	-0.10589	2.26312	0.00008
N	-0.73734	1.08560	-0.00004
O	-0.63471	-1.19256	-0.00025
H	1.94286	-0.93258	-0.00018
H	3.11042	1.27691	0.00004
H	1.72797	3.36617	0.00022
H	-0.74281	3.13823	0.00015
H	-1.57718	-0.99205	-0.00026

Frequencies (cm^{-1}):	218.01	422.31	424.38	495.46	560.3
	568.84	639.11	765.27	805.97	870.21
	892.43	1001.07	1015.6	1023.89	1074.58
	1118.4	1156.51	1208.36	1295.54	1340.15
	1371.46	1505.07	1528.79	1662.66	1685.8
	3186.03	3203.34	3231.36	3237.74	3816.55

1b

Atom	x	y	z
O	2.75479	0.31437	0.00002
C	1.54109	0.20956	-0.00004
C	0.77842	-1.02469	-0.00001
C	-0.57380	-1.01889	0.00000
C	-1.31502	0.19988	0.00000
C	-0.62237	1.35846	0.00000
N	0.73847	1.35377	-0.00001
H	-2.39196	0.20691	0.00001
H	-1.09238	2.33001	0.00001
H	1.34999	-1.93945	0.00001
H	-1.10951	-1.95852	0.00000
H	1.24906	2.22153	0.00000

Frequencies (cm^{-1}):	170.54	388.24	459.28	493.74	548.75
	617.22	702.21	746.87	782.49	833.43
	867.2	974.02	1003.03	1022.96	1028.42
	1119.68	1170.26	1227.7	1257.01	1394.82
	1462.75	1503.69	1615.83	1700.69	1782.46
	3196.53	3226.41	3237.72	3253.03	3639.36

2a

Atom	x	y	z
C	-0.05377	0.01649	0.02787
C	1.44140	-0.07212	0.14201
C	2.09911	1.24557	-0.25444
C	1.29170	2.40094	0.32306
C	-0.13564	2.34974	-0.20998
N	-0.77060	1.03974	-0.12888
O	-0.64198	-1.20238	0.12038
H	-1.59142	-1.04264	0.05790
H	1.78360	-0.90481	-0.47044
H	1.67801	-0.33271	1.17550
H	2.12237	1.33051	-1.34276
H	3.13186	1.26896	0.08948
H	-0.76762	3.05468	0.32930
H	-0.15271	2.66619	-1.25642
H	1.74243	3.36087	0.07220
H	1.27806	2.32574	1.41308

	123.82	238.01	318.95	396.02	464.08
	474.28	522.11	622.75	652.15	794.58
	842.08	887.73	928.34	948.95	980.11
	1080.01	1097.02	1122.6	1179.42	1210.3
Frequencies (cm^{-1}):	1232.36	1277.32	1308.53	1332.87	1367.03
	1380.97	1391.29	1431.05	1469.24	1484.95
	1492.03	1505.93	1795.12	3037.71	3045.96
	3055.54	3065.54	3088.48	3102.04	3106.3
	3116.11	3796.47			

2b

Atom	x	y	z
C	1.54459	0.25442	-0.04785
C	0.82381	-1.07400	-0.17727
C	-0.63307	-1.05034	0.27308
C	-1.33557	0.16154	-0.32424
C	-0.67290	1.43408	0.17477
N	0.77062	1.37535	-0.00645
O	2.75922	0.32262	-0.03215
H	1.29975	2.22291	0.11290
H	1.41578	-1.80579	0.36645
H	0.88561	-1.34620	-1.23307
H	-0.68564	-0.99685	1.36291
H	-1.12984	-1.97371	-0.02142
H	-2.39044	0.18136	-0.05394
H	-1.27714	0.12046	-1.41369
H	-1.05333	2.29979	-0.36616
H	-0.91702	1.57807	1.23195

Frequencies (cm^{-1}):	99.92	154.92	313.2	421.57	443.67
	492.2	534.36	650.7	668.62	780.95
	838.53	886.64	941.56	956.99	1008.75
	1077.46	1113.84	1134.22	1190.74	1209.79
	1280.55	1302.13	1330.95	1366.9	1388.42
	1389.96	1413.39	1461.11	1485.93	1493.8
	1507.78	1532.06	1779	3018.37	3048.99
	3057.73	3066.69	3090.89	3104.73	3111.06
	3135.11	3636.38			

3a

Atom	x	y	z
C	1.32413	0.08200	0.05870
C	1.09432	-1.27010	-0.02924
N	-0.12778	-1.80449	-0.11591
C	-1.12512	-0.93367	-0.11167
N	-1.04325	0.38878	-0.03220
C	0.17915	0.87962	0.05185
O	-2.36736	-1.42361	-0.19652
O	0.30467	2.20934	0.13356
H	1.91940	-1.97056	-0.03152
H	2.30998	0.50882	0.12830
H	-0.58757	2.57559	0.11341
H	-2.27770	-2.38152	-0.24688

Frequencies (cm^{-1}):	196.75	228.62	360.55	463.04	524.78
	557.93	563.09	566.45	601.91	739.88
	803.15	819.99	846.37	1013.12	1025.37
	1048.72	1109.65	1205.77	1243.54	1296.64
	1374	1412.45	1498.6	1536.35	1675.79
	1684.91	3192.48	3259.41	3802.08	3826.21

3b

Atom	x	y	z
C	1.42200	0.16181	-0.07209
C	1.21030	-1.15465	0.03253
N	-0.05156	-1.68777	0.10490
C	-1.20428	-0.92182	0.07644
N	-0.95816	0.42917	-0.03195
C	0.28397	1.07385	-0.11180
O	-2.31058	-1.40248	0.14085
O	0.34166	2.27800	-0.20367
H	2.01639	-1.87166	0.06600
H	-0.19669	-2.67800	0.18337
H	-1.77321	1.02156	-0.05584
H	2.41185	0.57900	-0.12874

Frequencies (cm^{-1}):	147.68	162.55	392.38	395.97	520.69
	546.02	562.35	563.64	680.46	737.22
	778.42	781.22	827.36	977.66	996.35
	996.83	1096.16	1211.22	1235.51	1396.82
	1423.56	1432.49	1517.15	1711.48	1807.91
	1831.46	3230.56	3272.75	3635.91	3681.34

4a

Atom	x	y	z
C	-2.87182	-2.08711	-0.12187
N	-1.74173	-2.10663	0.56772
C	-1.32991	-0.89751	0.93227
N	-1.92778	0.25515	0.67366
C	-3.04915	0.11811	-0.02509
N	-3.58137	-1.01504	-0.45604
O	-3.71423	1.22677	-0.32901
O	-3.33476	-3.26812	-0.51522
O	-0.20175	-0.82526	1.62933
H	-3.21524	1.96264	0.04437
H	0.11625	-1.72890	1.73984
H	-4.15175	-3.10044	-0.99930

Frequencies (cm^{-1}):	200.55	200.94	215.58	376.34	376.49
	584.73	585.86	586.88	586.98	589.65
	626.49	682.81	781.18	781.38	858.05
	1011.74	1110.65	1110.9	1239.88	1261.4
	1261.59	1377.67	1498.14	1498.37	1557.6
	1689.55	1689.63	3814.24	3814.4	3815.44

4b

Atom	x	y	z
O	-3.35791	-3.30617	-0.53359
C	-2.94244	-2.23483	-0.18065
C	-1.23899	-0.87958	0.98855
O	-0.21903	-0.80895	1.62074
C	-3.16892	0.22403	-0.08988
O	-3.77500	1.22453	-0.36668
N	-3.58406	-1.04199	-0.46272
N	-1.77632	-2.07548	0.54668
N	-1.98878	0.22722	0.63223
H	-1.64201	1.12393	0.92938
H	-1.26988	-2.91319	0.77995
H	-4.43881	-1.10129	-0.99048

Frequencies (cm^{-1}):	129.25	149.97	150.47	396.76	396.93
	519.55	519.69	595.34	595.62	638.49
	654.68	676.29	767.53	767.75	774.77
	995.33	1040.27	1040.29	1237.22	1384.75
	1414.88	1414.94	1487.49	1487.65	1842.17
	1842.43	1868.27	3658.11	3658.33	3661.16

5a

Atom	x	y	z
N	-0.20465	-1.19267	0.00000
C	1.11475	-1.08457	0.00000
N	1.81618	0.04603	0.00000
C	1.06572	1.14042	0.00000
N	-0.25886	1.17600	0.00000
C	-0.83333	-0.02489	0.00000
O	-2.16215	-0.07056	0.00000
H	1.57864	2.09426	0.00000
H	1.67640	-2.01054	0.00000
H	-2.47609	0.84099	0.00000

	189.88	377.14	464.12	558.16	596.72
	597.4	711.99	856.82	923.63	1009.63
Frequencies (cm^{-1}):	1021.24	1064.88	1156.88	1198.72	1247.11
	1374.91	1403.49	1438.82	1509.54	1646.64
	1666.77	3190.92	3194.13	3816.92	

5b

Atom	x	y	z
O	-2.21175	-0.09310	0.00000
C	-1.00976	-0.11114	0.00000
N	-0.21317	-1.24452	0.00000
C	1.06932	-1.08885	0.00000
N	1.79026	0.07967	0.00000
C	1.05492	1.14174	0.00000
N	-0.28705	1.10523	0.00000
H	1.51634	2.12209	0.00000
H	-0.83481	1.95065	0.00000
H	1.67175	-1.99002	0.00000

	128.79	365.43	497.83	505.69	571.93
	684.4	730.83	825.56	861.68	996.77
Frequencies (cm^{-1}):	997.08	1054.02	1071.98	1156.1	1238.05
	1400.01	1434.64	1483.26	1589.74	1705.53
	1840.73	3180.45	3189.05	3635.04	

6a

Atom	x	y	z
N	0.00000	0.00000	-0.99808
C	1.10292	0.00001	-0.27056
N	1.18022	0.00000	1.06267
C	0.00000	0.00000	1.66231
N	-1.18022	0.00000	1.06267
C	-1.10292	0.00000	-0.27056
O	2.24747	0.00000	-0.94494
O	-2.24746	0.00000	-0.94494
H	2.95685	-0.00001	-0.29210
H	-2.95684	0.00000	-0.29210
H	0.00000	0.00000	2.74517

Frequencies (cm^{-1}):	191.36	206.07	382.19	473.79	544.26
	585.67	588.1	595.62	607.37	784.35
	851.16	861.52	1015.45	1038.86	1061.24
	1205.11	1226.88	1316.26	1376.43	1394.85
	1487.22	1543.12	1663	1685.37	3193.81
	3817.88	3819.01			

6b

Atom	x	y	z
O	2.28136	0.95249	-0.00001
C	1.22967	0.36739	0.00001
C	-1.25342	0.30561	0.00003
O	-2.26675	0.94660	-0.00003
N	-1.21783	-1.09526	0.00000
C	-0.07308	-1.65940	0.00000
N	-0.00707	0.95886	0.00001
N	1.13170	-1.02176	0.00000
H	-0.01639	-2.74206	-0.00001
H	1.99702	-1.53310	0.00000
H	-0.02657	1.96617	-0.00001

Frequencies (cm^{-1}):	113.92	157.39	404.34	414.26	516.75
	569.41	598.76	604.93	679.09	776.54
	809.28	810.35	958.91	1007.72	1011.84
	1126.19	1202.01	1404.11	1406.57	1429.94
	1502.2	1720.3	1839.35	1857.54	3180.83
	3640.74	3667.91			

7a

Atom	x	y	z
N	1.02849	1.22572	0.14991
C	1.56967	0.00851	0.18919
C	0.85189	-1.16369	0.05446
C	-0.52347	-1.05795	-0.13015
C	-1.10897	0.18692	-0.16680
C	-0.28735	1.31419	-0.02102
H	2.64292	-0.02038	0.33290
H	1.34748	-2.12126	0.09122
H	-1.13246	-1.94440	-0.24129
H	-2.17540	0.30290	-0.29670
N	-0.82302	2.58674	-0.00345
H	-0.14401	3.31632	-0.13422
H	-1.68774	2.72341	-0.49403

Frequencies (cm^{-1}):	204.93	367.89	406.67	419.97	495.91
	556.78	578.86	639.81	760.72	801.46
	868.91	881.01	1000.75	1010.41	1017.82
	1066.52	1080.38	1150.58	1167.83	1292.33
	1347.06	1356.13	1491.4	1532.43	1635.1
	1652.71	1681.07	3174.87	3194.64	3211.4
	3231.16	3611.22	3723.94		

7b

Atom	x	y	z
N	-0.73036	2.57391	0.18895
C	-0.32854	1.36135	0.11520
C	-1.10111	0.14345	-0.07910
C	-0.50965	-1.06711	-0.13306
C	0.90874	-1.21172	-0.00174
C	1.63504	-0.08974	0.17863
N	1.04174	1.13199	0.23361
H	2.70875	-0.09302	0.28804
H	1.38715	-2.17534	-0.04402
H	-1.11757	-1.94964	-0.27862
H	-2.17125	0.24951	-0.17842
H	-1.73726	2.61794	0.09019
H	1.59496	1.96125	0.36858

	144.7	381.66	429.33	474.41	548.38
	623.78	669.71	725.9	759.77	776.12
	834.64	849.31	970.53	1004	1016.79
Frequencies (cm^{-1}):	1022.63	1087.65	1155.67	1187.52	1243.22
	1362.36	1409.33	1460.29	1513.89	1613.99
	1684.65	1739.86	3195.01	3219.41	3226.36
	3255.53	3554.86	3647.41		

7b'

Atom	x	y	z
N	-0.81410	2.53377	0.17627
C	-0.27905	1.37300	0.12141
C	-1.07094	0.16803	-0.07347
C	-0.50668	-1.05214	-0.13130
C	0.91296	-1.22423	-0.00261
C	1.66240	-0.12270	0.17832
N	1.09201	1.11955	0.23779
H	2.73600	-0.14732	0.28567
H	1.37237	-2.19720	-0.04792
H	-1.13134	-1.92241	-0.27745
H	-2.13490	0.31650	-0.16806
H	-0.12247	3.26536	0.31600
H	1.68945	1.91555	0.37419

	146.34	373.39	437.82	470.63	542.32
	594.53	623.42	714.41	757.50	792.28
	829.14	856.01	964.02	1000.06	1014.11
Frequencies (cm^{-1}):	1025.45	1090.02	1151.67	1182.74	1241.33
	1336.30	1412.98	1466.26	1501.67	1623.57
	1692.83	1747.70	3200.28	3225.28	3239.90
	3253.46	3501.12	3658.00		

8a

Atom	*x*	*y*	*z*
C	1.75054	-0.00250	0.17624
C	1.02663	-1.14424	-0.07137
C	-0.36273	-0.97678	-0.17632
N	-0.93500	0.21547	-0.05250
C	-0.11771	1.24714	0.18189
N	1.21050	1.20793	0.31130
H	2.82837	-0.05247	0.27645
H	1.49613	-2.11034	-0.16691
N	-1.20054	-2.03704	-0.37462
N	-0.70493	2.47459	0.26885
H	-2.14088	-1.80879	-0.64174
H	-0.81836	-2.88689	-0.74300
H	-0.14831	3.21193	0.65739
H	-1.69645	2.49739	0.41145

Frequencies (cm^{-1}):	182.16	218.07	330.98	363.84	373.18
	447.52	462.68	494.44	517.31	562.22
	607.87	729.53	794.87	806.72	835.11
	996.31	1009.78	1021.34	1057.19	1131.89
	1183.07	1280.78	1347.45	1405.89	1488.64
	1523.31	1625.41	1634.23	1659	1671.35
	3172.88	3231.59	3627.26	3639.13	3749.02
	3765.68				

8b

Atom	*x*	*y*	*z*
C	1.72252	-0.03525	0.18232
C	1.06115	-1.18426	0.00483
C	-0.38993	-1.16101	-0.13581
N	-0.94945	0.11750	-0.06638
C	-0.28864	1.31374	0.11634
N	1.08548	1.17325	0.23775
H	2.79568	0.00327	0.29180
H	1.58030	-2.12645	-0.03620
N	-1.18879	-2.13642	-0.30957
N	-0.79189	2.47946	0.18134
H	-1.79908	2.46302	0.08064
H	1.58899	2.03024	0.37160
H	-0.67707	-3.01008	-0.34433
H	-1.95031	0.14545	-0.16190

Frequencies (cm^{-1}):	112.75	134.69	370.92	375.4	504.98
	510.64	533.54	567.94	586.83	702.55
	720.9	760.85	769.98	780.2	841.03
	972.54	982.88	995.67	1088.95	1144.33
	1162.34	1228.23	1357.64	1416.83	1424.75
	1476.53	1523.97	1692.97	1752.07	1760.29
	3227.12	3258.26	3552.91	3565.93	3651.2
	3691.08				

9a

Atom	*x*	*y*	*z*
C	1.61888	-0.00980	0.00110
N	0.91267	-1.11521	-0.24492
C	-0.40751	-0.91961	-0.23769
N	-1.03419	0.24049	-0.03110
C	-0.20667	1.26438	0.18734
N	1.12624	1.21051	0.22359
N	2.96563	-0.14270	0.04773
N	-1.18766	-2.00748	-0.44154
N	-0.77433	2.48100	0.36887
H	-2.16366	-1.86117	-0.61059
H	-0.74717	-2.84948	-0.75706
H	-0.19236	3.22241	0.70730
H	-1.76323	2.51701	0.52275
H	3.36457	-1.00337	-0.27269
H	3.52031	0.69082	0.05908

Frequencies (cm^{-1}):	183.83	184.58	206.99	250.05	274.32
	288.62	339.36	339.46	515.02	539.67
	544.92	564.69	584.17	584.37	689.77
	767.25	768.05	850.22	1000.06	1013.84
	1014.74	1154.97	1185.45	1186.1	1312.75
	1484.73	1485.03	1516.34	1622.05	1622.15
	1648.2	1648.66	1670.67	3645.9	3647.28
	3648.75	3777.17	3779.96	3780.31	

9b

Atom	*x*	*y*	*z*
C	-3.71421	5.06297	0.33678
N	-2.39660	5.27536	-0.01346
N	-4.37304	4.16941	-0.49226
C	-3.83419	3.51423	-1.58048
H	-5.33384	4.00413	-0.25200
H	-1.84310	5.90733	0.53649
C	-1.72385	4.68801	-1.07283
N	-2.50333	3.82222	-1.81221
H	-2.09499	3.35236	-2.60004
N	-4.34981	5.58469	1.30148
H	-3.77356	6.21930	1.84003
N	-0.50596	4.98355	-1.26315
H	-0.11545	4.49858	-2.06134
N	-4.41552	2.69421	-2.35281
H	-5.38192	2.54307	-2.09195

Frequencies (cm^{-1}):	90.24	105.99	106.73	374.71	375
	471.56	471.72	529.94	529.98	567.3
	599.46	680.14	697.89	702.88	702.95
	777.71	777.92	779.39	990.29	1030.51
	1030.57	1137.09	1137.37	1159.13	1374.71
	1411.3	1423.02	1423.11	1515.59	1515.69
	1766.18	1772.13	1772.3	3567.49	3567.67
	3568.01	3677.02	3677.26	3679.71	

10a

Atom	x	y	z
N	0.66607	1.37375	0.04061
C	-0.66193	1.33354	0.03909
C	-1.39856	0.15937	0.00546
C	-0.70685	-1.03921	-0.02848
C	0.67726	-1.01040	-0.02726
C	1.33189	0.21707	0.00791
C	2.83091	0.31053	0.01159
H	1.25027	-1.92592	-0.05326
H	-1.23535	-1.98191	-0.05558
H	-2.47780	0.19016	0.00596
H	-1.16493	2.29274	0.06619
H	3.29562	-0.67187	-0.02350
H	3.16956	0.82815	0.90744
H	3.16993	0.89138	-0.84437

Frequencies (cm^{-1}):	59.5	202.71	357.75	417.73	485.92
	554.08	639.11	758.9	773.8	827.59
	912.67	998.64	998.72	1025.12	1032.82
	1065.21	1085.14	1132.35	1164.93	1260.57
	1277.61	1333.4	1410.23	1466.52	1473.75
	1497.29	1527.99	1651.54	1671.66	3069
	3132.26	3147.77	3172.52	3196.01	3213.06
	3224.58				

10b

Atom	x	y	z
N	0.618452	1.339603	0.155400
C	-0.746769	1.343742	0.114811
C	-1.451405	0.205433	-0.005223
C	-0.712784	-1.029142	-0.090331
C	0.629862	-1.039142	-0.050977
C	1.407139	0.185505	0.078753
C	2.748336	0.235847	0.123253
H	1.183493	-1.963798	-0.114779
H	-1.251681	-1.961240	-0.187552
H	-2.527528	0.226501	-0.035716
H	-1.208095	2.317099	0.185599
H	1.097539	2.216145	0.245944
H	3.280010	1.171133	0.220365
H	3.324102	-0.672423	0.061486

Frequencies (cm^{-1}):	125.19	363.77	369.59	441.83	536.31
	548.95	626.28	669.65	689.93	735.28
	759.01	824.84	834.20	949.58	960.53
	999.92	1013.44	1014.85	1135.72	1182.08
	1243.19	1337.00	1395.57	1418.15	1488.48
	1514.72	1622.54	1687.01	1737.97	3178.77
	3197.94	3222.78	3223.53	3253.30	3272.66
	3676.39				

11a

Atom	x	y	z
C	1.34718	0.27246	-0.03215
C	0.86214	-0.99422	0.25430
C	-0.51274	-1.14528	0.28481
N	-1.35018	-0.14320	0.05481
C	-0.79264	1.04123	-0.21280
N	0.51332	1.28901	-0.26546
C	2.81309	0.57694	-0.09712
H	1.52478	-1.82440	0.44477
H	-0.96096	-2.10771	0.50228
H	3.41706	-0.30407	0.10384
H	3.06009	1.35154	0.62671
H	3.06469	0.96492	-1.08255
C	-1.72838	2.18244	-0.47503
H	-1.17071	3.09087	-0.67582
H	-2.37891	2.33245	0.38486
H	-2.36797	1.94851	-1.32438

	19.65	69.48	184.38	204.88	287.71
	427.1	442.57	547.46	583.09	590.47
	762.39	782.75	851.52	942.4	999.92
	1022.1	1023.49	1051.3	1061.67	1071.86
Frequencies (cm^{-1}):	1137.49	1213.11	1286.22	1326.55	1398.66
	1405.71	1443.88	1472.53	1476.96	1477.76
	1488.44	1504.34	1646.61	1659.38	3072.07
	3075.7	3134.77	3136.71	3154.82	3172.8
	3177.25	3226.66			

11b

Atom	x	y	z
C	1.48575	0.20208	-0.01234
C	0.69611	-1.01884	0.07805
C	-0.63528	-0.95071	0.02703
N	-1.30400	0.24346	-0.10873
C	-0.64858	1.46372	-0.21159
N	0.72943	1.36819	-0.14696
C	2.82259	0.23015	0.02894
H	1.20038	-1.96351	0.18585
H	-1.26474	-1.82503	0.09013
C	-1.30342	2.62424	-0.35756
H	3.37557	1.15550	-0.04098
H	3.37724	-0.68732	0.13498
H	-2.37994	2.64507	-0.39934
H	-0.76626	3.55504	-0.43383
H	1.22913	2.23462	-0.21816
H	-2.30312	0.25626	-0.15503

	41.41	102.13	291.93	318.9	377.33
	433.82	462.36	524.5	571.06	625.03
	641.81	667.01	686.73	710.9	766.53
	770.29	801.62	918.52	938.65	964.95
Frequencies (cm^{-1}):	992.72	1064.23	1124.6	1230.84	1341.64
	1363.62	1417.7	1440.66	1442.11	1500.1
	1527.18	1687.94	1735.28	1755.65	3179.74
	3195.81	3227.93	3260.8	3273.64	3284.84
	3680.92	3717.4			

12a

Atom	x	y	z
C	1.18184	0.13002	0.11348
N	0.56818	-1.03355	0.28228
C	-0.76667	-0.99060	0.22588
N	-1.47721	0.10984	0.01815
C	-0.76520	1.23008	-0.13966
N	0.55901	1.29317	-0.10093
C	2.67625	0.15761	0.16314
C	-1.52896	2.49428	-0.37412
C	-1.49719	-2.28216	0.41237
H	-1.24481	-2.70748	1.38211
H	-1.17596	-2.99639	-0.34371
H	-2.56824	-2.12657	0.34389
H	-2.18739	2.68477	0.47177
H	-2.16131	2.38243	-1.25293
H	-0.85009	3.32924	-0.50827
H	3.06867	-0.83511	0.35437
H	3.00376	0.84404	0.94183
H	3.06595	0.53499	-0.78074

	18.02	23.74	31.19	180.64	181.27
	184.93	291.15	291.25	489.24	574.21
	574.26	598.3	598.47	623.88	798.15
	957.5	957.56	1016.54	1053.58	1054.47
Frequencies (cm^{-1}):	1054.52	1059.24	1071.51	1071.56	1189.65
	1371.78	1384.02	1384.12	1435.31	1435.39
	1455.63	1473.94	1475.35	1475.45	1480.11
	1480.24	1486.66	1635.84	1636.02	3077.12
	3077.16	3077.3	3137.69	3137.74	3137.77
	3180.63	3181.05	3181.22		

12b

Atom	x	y	z
C	1.29271	0.13331	0.15829
N	0.54699	-1.00848	0.39691
C	-0.82465	-1.11483	0.24193
N	-1.46726	0.10076	0.08126
C	-0.84175	1.30904	-0.17417
N	0.53097	1.27871	0.00239
C	2.63037	0.13568	0.11804
C	-1.50782	2.41537	-0.52538
C	-1.47449	-2.28410	0.28021
H	-2.58012	2.40181	-0.62822
H	-0.98478	3.34246	-0.69140
H	3.18629	-0.77464	0.26931
H	3.17356	1.05143	-0.04611
H	-2.54650	-2.32070	0.17992
H	-0.93829	-3.20601	0.43249
H	-2.45405	0.06486	-0.08754
H	1.05658	-1.86880	0.45805
H	1.02866	2.11791	-0.22474

	50.59	56.52	58.76	319.43	319.67
	347.8	348.5	494.03	494.54	535.79
	535.99	617.47	621.32	621.49	634.66
	667.27	667.8	676.35	712.72	713.13
Frequencies (cm^{-1}):	723.17	915.44	915.67	982.11	1035.03
	1087.86	1087.93	1347.39	1347.51	1407.03
	1433.27	1444.38	1470.65	1470.71	1516.24
	1516.3	1728.52	1749.8	1749.87	3196.98
	3197.24	3197.6	3286.59	3286.8	3286.97
	3698.11	3698.37	3699.87		

13a

Atom	x	y	z
C	1.64538	-0.01663	0.19700
N	0.97296	-1.11591	-0.07959
C	-0.34656	-0.91016	-0.19234
N	-0.97680	0.25904	-0.05863
C	-0.16732	1.28095	0.21575
N	1.16242	1.20881	0.36197
O	2.96755	-0.15969	0.32504
N	-1.10600	-1.99196	-0.45500
N	-0.73104	2.50035	0.34441
H	-2.08204	-1.86857	-0.63764
H	-0.65258	-2.86372	-0.64574
H	-0.16523	3.26195	0.66301
H	-1.72954	2.56912	0.35573
H	3.31617	0.71680	0.52062

Frequencies (cm^{-1}):	154.01	177.34	189.76	195.77	212.28
	340.05	360.83	547.21	563.29	567.83
	577.17	580.78	588.85	687.73	767.66
	775.3	851.75	1000.9	1010.43	1064.47
	1173.89	1179.27	1256.11	1333.52	1478.13
	1500.26	1523.91	1627.05	1633.03	1665.93
	1686.8	3652.19	3653.72	3786.87	3789.23
	3825.91				

13b

Atom	x	y	z
C	1.79906	-0.13279	0.22341
N	0.97479	-1.16771	-0.07409
C	-0.31021	-0.91579	-0.19796
N	-0.95645	0.28123	-0.05688
C	-0.17234	1.28343	0.22452
N	1.15762	1.13405	0.37549
O	2.99834	-0.17475	0.36787
N	-1.11994	-1.94881	-0.49397
N	-0.69731	2.52945	0.34068
H	-2.09914	-1.78900	-0.62084
H	-0.71635	-2.85476	-0.63057
H	-0.19069	3.23909	0.83543
H	-1.69945	2.57099	0.37856
H	1.76892	1.91241	0.55268

Frequencies (cm^{-1}):	116.64	145.54	186.36	194.93	316.96
	336.22	378.77	475.83	546.95	562.46
	578.36	589.73	605.45	687.49	745.02
	781.24	817.93	952.38	997.44	1070.72
	1108.52	1181.66	1270.69	1335.6	1485.77
	1504.9	1595.33	1626.75	1669.56	1690.69
	1822.61	3629.92	3652.97	3655.81	3749.24
	3792.47				

14a

Atom	x	y	z
C	1.64366	-0.01982	0.19140
N	0.96581	-1.12058	-0.09157
C	-0.34529	-0.90960	-0.19401
N	-0.98723	0.23227	-0.04673
C	-0.18159	1.26180	0.23652
N	1.15066	1.19618	0.36890
O	2.96331	-0.16466	0.30730
O	-1.09789	-1.97103	-0.47786
N	-0.75841	2.46400	0.40180
H	-0.49593	-2.72004	-0.55461
H	-0.19090	3.25952	0.61659
H	-1.75197	2.54447	0.31073
H	3.31906	0.70728	0.51143

	127.78	191.95	199.61	214.26	350.1
	375.94	568.73	578.05	580.07	583.64
	588.86	597.65	685.04	772	779.96
Frequencies (cm^{-1}):	854.42	1005.89	1035.51	1108.05	1174.99
	1246.66	1266.15	1352.33	1487.41	1501.02
	1535.42	1649.82	1655.75	1700.26	3654.21
	3790.76	3818.51	3823.7		

14b

Atom	x	y	z
C	1.86399	-0.02210	0.17798
N	1.01792	-1.06291	-0.07135
C	-0.38719	-0.98460	-0.20640
N	-0.95388	0.26188	-0.03662
C	-0.18166	1.26654	0.20666
N	1.18174	1.18243	0.33024
O	3.06441	-0.09590	0.26324
O	-1.00677	-1.98659	-0.44963
N	-0.71236	2.50841	0.32560
H	1.43340	-1.97094	-0.19704
H	-0.21066	3.23045	0.80725
H	-1.71541	2.54000	0.36351
H	1.75961	1.99603	0.44857

	117.55	138.11	199.15	297.43	352.83
	403.69	487	526.9	539.52	548.29
	632.75	642.65	682.2	745.42	775.21
Frequencies (cm^{-1}):	799.06	967.83	1010.38	1063.59	1140.66
	1264.19	1340.82	1406.69	1427.23	1554.16
	1632.78	1706.86	1829.57	1849.29	3626.25
	3654	3665.61	3746.75		

Phenol (enol form)

Atom	x	y	z
C	0.044771	-0.00834	-0.94166
C	0.021864	-1.20866	-0.24285
C	-0.00673	-1.19219	1.141107
C	-0.01278	0.009604	1.837795
C	0.010354	1.20177	1.131575
C	0.039062	1.199086	-0.25563
O	0.072478	-0.07476	-2.3054
H	0.087305	0.814054	-2.66536
H	0.026872	-2.1363	-0.79595
H	0.006057	2.145313	1.658961
H	-0.03518	0.015086	2.91745
H	0.057002	2.132769	-0.804
H	-0.0245	-2.12886	1.680451

	230.99	342.27	407.89	423	519.91
	535.99	631	705.04	772.52	838.25
	839.61	908.35	988.49	1003.7	1021.09
Frequencies (cm^{-1}):	1056.93	1102.79	1166.7	1191.92	1201.8
	1297.29	1332.99	1372.29	1517.42	1546.93
	1674.28	1684.28	3177.07	3195.15	3204.04
	3218.15	3224.95	3873.17		

Phenol (keto form)

Atom	x	y	z
C	-2.05711	0.276088	-0.09465
C	-1.26198	0.279991	-1.33268
C	0.074952	0.33207	-1.29522
C	0.824507	0.388933	-0.04376
C	0.191488	0.393039	1.127375
C	-1.29772	0.341022	1.21976
O	-3.26882	0.224622	-0.1064
H	-1.61212	-0.51827	1.817399
H	-1.81286	0.237061	-2.26101
H	0.750058	0.435456	2.052983
H	1.903189	0.42765	-0.08929
H	-1.67411	1.209715	1.766137
H	0.635038	0.332305	-2.22129

	49.03	267.57	444.08	458.33	493.16
	538.05	578.17	730.51	755.43	814.51
	948.23	953.04	958.99	1003.87	1020.55
Frequencies (cm^{-1}):	1042.24	1166.02	1195.83	1201.13	1255.33
	1352.19	1404.13	1409.24	1452.37	1663.18
	1734.31	1784.6	3054.22	3080.36	3184.37
	3190.24	3214.44	3221.38		

Resorcinol (enol form)

Atom	x	y	z
C	0.963992	0.080609	1.077724
C	1.646679	0.000447	-0.12808
C	0.918388	-0.08794	-1.30698
C	-0.46401	-0.09748	-1.30106
C	-1.12944	-0.01636	-0.0823
C	-0.4241	0.072703	1.10797
H	-1.03656	-0.16596	-2.21308
H	1.444635	-0.15069	-2.24895
H	2.728381	0.006953	-0.14521
H	-0.93131	0.136421	2.06128
O	-2.49255	-0.02825	-0.11037
H	-2.82921	0.03289	0.785654
O	1.605292	0.170083	2.278693
H	2.55275	0.166931	2.129573

Frequencies (cm^{-1}):	224.55	236.11	333.14	345.45	356.97
	465.41	481.1	529.35	545.07	637.67
	698.19	762.55	775.82	853.09	871.95
	984.65	990.03	1018.64	1106.01	1167.41
	1175.66	1209.08	1234.99	1340.72	1347.21
	1369.77	1533.42	1552.36	1682.9	1694.1
	3187.26	3198.27	3207.42	3231.19	3872.55
	3873.6				

Resorcinol (keto form)

Atom	x	y	z
O	1.504181	-0.75469	2.11156
C	0.971382	-0.20703	1.182444
O	-2.42177	-0.36981	-0.04551
C	-1.23618	-0.14255	-0.06424
C	-0.44935	-0.16302	-1.31636
C	0.875184	-0.03807	-1.32357
C	1.719143	0.120313	-0.09423
C	-0.47982	0.210171	1.206953
H	-1.01286	-0.30167	-2.22783
H	1.403962	-0.06436	-2.26793
H	2.611679	-0.50058	-0.14296
H	2.066449	1.156343	-0.02196
H	-0.5006	1.302899	1.280052
H	-0.98731	-0.19968	2.074309

Frequencies (cm^{-1}):	86.94	91.56	303.71	378.49	417.85
	457.59	525.24	557.94	586.93	697.67
	724.76	838.25	874.7	935.7	971
	998.24	1041.94	1107.66	1161.44	1210.82
	1231.21	1280.33	1300.2	1350.83	1414.4
	1427.12	1443.1	1719.35	1803.97	1842.76
	3040.8	3045.92	3119.63	3162.67	3184.02
	3218.3				

Phloroglucinol (enol form)

Atom	x	y	z
C	-1.98494	0.519952	-0.20441
C	-1.27544	0.216836	-1.35551
C	0.102973	0.063883	-1.2662
C	0.768869	0.207909	-0.05953
C	0.028406	0.511905	1.076595
C	-1.34704	0.670973	1.021023
O	-3.33725	0.684361	-0.216
H	-3.66202	0.557129	-1.10954
H	-1.76527	0.097807	-2.31142
H	0.108017	0.85186	2.950501
H	1.840418	0.090485	0.016979
H	-1.92879	0.907606	1.900469
H	1.700537	-0.31416	-2.23479
O	0.762905	-0.23351	-2.42029
O	0.720872	0.645021	2.242194

Frequencies (cm^{-1}):	205.95	238.21	238.33	331.67	331.95
	355.23	356.25	370.95	520.5	520.56
	558.81	609.56	631.9	632.32	688.59
	816.41	816.63	831.16	1017.43	1033.76
	1033.86	1166.75	1179.16	1179.38	1236.01
	1236.25	1329.92	1375.52	1414.24	1553.24
	1553.41	1698.23	1698.28	3208.41	3208.5
	3209.64	3872.65	3872.75	3873.12	

Phloroglucinol (keto form)

Atom	x	y	z
O	1.533908	-0.60804	2.173117
C	0.982337	-0.14789	1.210231
O	1.519205	-0.86945	-2.22272
C	0.974025	-0.29774	-1.31772
O	-2.28544	-0.80113	-0.00918
C	-1.21441	-0.25821	-0.04467
C	-0.50571	0.037016	-1.35359
C	1.727631	0.14767	-0.07821
C	-0.49744	0.188542	1.215879
H	2.715075	-0.30026	-0.05495
H	1.835888	1.235843	-0.14298
H	-0.57729	1.280199	1.259856
H	-0.97733	-0.23191	2.092903
H	-0.99116	-0.48337	-2.17199
H	-0.58589	1.116025	-1.52479

Frequencies (cm^{-1}):	74.74	75.59	94.5	368.13	368.22
	423.18	425.16	425.8	556.76	557.38
	591.03	619.72	801.91	802.12	854.9
	855.15	927.37	1017.76	1052.32	1162.73
	1191.52	1191.66	1239.14	1267.83	1268.01
	1295.92	1296.11	1427.18	1427.4	1441.58
	1827.95	1828.1	1862.47	3042.56	3042.9
	3049.42	3170.43	3170.77	3171.92	

Table S10. Optimized cartesian coordinates (Å) of all studied molecules at the ωB97M-D4rev/Def2-TZVPP level and CPCM (water) level and their corresponding frequencies (cm^{-1}).

1a

Atom	x	y	z
C	0.002251	-0.001462	-0.000126
C	1.398077	0.001863	-0.000098
C	2.031335	1.225434	0.000024
C	1.269734	2.393318	0.000117
C	-0.103671	2.270150	0.000084
N	-0.739855	1.090458	-0.000037
O	-0.633595	-1.194498	-0.000252
H	1.941944	-0.930165	-0.000176
H	3.111040	1.274803	0.000044
H	1.731734	3.368398	0.000214
H	-0.736682	3.148007	0.000152
H	-1.583278	-1.021861	-0.000260

Frequencies (cm^{-1}):	216.72	420.92	421.97	451.48	544.60
	566.28	637.26	764.48	806.10	864.59
	894.07	1008.39	1013.47	1035.28	1072.37
	1114.13	1149.54	1191.88	1284.62	1324.31
	1364.15	1492.38	1517.64	1649.93	1677.62
	3189.41	3208.28	3231.29	3236.02	3790.05

1b

Atom	x	y	z
O	2.760462	0.311054	0.000065
C	1.527943	0.211837	-0.000157
C	0.780909	-1.019914	-0.000011
C	-0.577112	-1.018312	-0.000006
C	-1.313806	0.195584	-0.000029
C	-0.617989	1.356509	0.000007
N	0.741529	1.348551	-0.000025
H	-2.391033	0.204392	-0.000012
H	-1.078655	2.331948	0.000057
H	1.348870	-1.937743	0.000048
H	-1.110160	-1.959357	0.000035
H	1.235823	2.228381	0.000027

Frequencies (cm^{-1}):	185.52	394.54	463.35	504.64	554.66
	622.58	708.40	759.63	790.42	848.45
	869.31	996.60	1003.63	1032.66	1036.70
	1119.94	1164.84	1227.83	1266.33	1392.63
	1459.79	1494.79	1607.95	1657.40	1712.31
	3202.70	3230.08	3239.52	3255.28	3608.56

3a

Atom	x	y	z
C	1.322729	0.083700	0.059141
C	1.102453	-1.268098	-0.029022
N	-0.121153	-1.810828	-0.116738
C	-1.121306	-0.945792	-0.112367
N	-1.045907	0.378912	-0.032482
C	0.175421	0.879644	0.052121
O	-2.368034	-1.426911	-0.197251
O	0.305440	2.205643	0.133957
H	1.933155	-1.961205	-0.030979
H	2.308747	0.511286	0.129016
H	-0.575272	2.603269	0.114290
H	-2.313395	-2.389410	-0.247798

Frequencies (cm^{-1}):	194.98	228.21	362.96	455.39	500.76
	510.23	527.35	562.22	601.03	737.03
	800.59	817.77	844.81	1011.89	1032.89
	1047.57	1110.95	1185.34	1233.30	1287.96
	1360.52	1402.58	1471.11	1516.42	1654.17
	1666.08	3202.19	3257.81	3774.20	3798.00

3b

Atom	x	y	z
C	1.416669	0.165228	-0.072122
C	1.204923	-1.155535	0.033159
N	-0.053283	-1.684941	0.105233
C	-1.191562	-0.919893	0.076140
N	-0.954981	0.430199	-0.030908
C	0.282145	1.063820	-0.111076
O	-2.312552	-1.392519	0.139943
O	0.337259	2.280880	-0.205109
H	2.007214	-1.876045	0.067140
H	-0.180508	-2.680869	0.181487
H	-1.773525	1.020807	-0.055392
H	2.409869	0.575889	-0.128482

Frequencies (cm^{-1}):	163.23	173.67	392.55	406.44	527.82
	553.12	570.14	595.30	659.78	738.89
	784.49	788.48	824.97	994.72	1003.03
	1013.21	1104.55	1214.23	1241.17	1402.20
	1429.05	1441.67	1521.66	1693.35	1712.43
	1759.65	3246.00	3268.45	3613.08	3646.81

4a

Atom	x	y	z
C	-2.882288	-2.083792	-0.128159
N	-1.749365	-2.112116	0.562091
C	-1.326852	-0.909586	0.932921
N	-1.919678	0.249885	0.678011
C	-3.041876	0.126777	-0.020360
N	-3.581928	-1.004328	-0.456027
O	-3.709703	1.229305	-0.326819
O	-3.339017	-3.265148	-0.517199
O	-0.202377	-0.830548	1.629268
H	-3.235544	1.989581	0.035775
H	0.146918	-1.722488	1.758817
H	-4.161530	-3.133884	-1.007658

Frequencies (cm^{-1}):	197.91	198.74	216.29	379.37	380.11
	534.24	535.64	538.56	586.33	586.53
	632.47	685.63	778.63	778.88	852.08
	1011.64	1108.65	1110.54	1234.79	1247.15
	1250.20	1363.34	1465.10	1465.19	1542.46
	1653.03	1653.81	3779.57	3780.63	3782.31

4b

Atom	x	y	z
O	-3.357681	-3.303166	-0.531565
C	-2.939034	-2.224915	-0.176576
C	-1.247999	-0.880256	0.982654
O	-0.220691	-0.808634	1.617600
C	-3.163433	0.215096	-0.087834
O	-3.773199	1.222443	-0.365676
N	-3.582589	-1.041644	-0.463349
N	-1.777552	-2.074729	0.547897
N	-1.989563	0.225884	0.632153
H	-1.642689	1.124291	0.931207
H	-1.268555	-2.914319	0.779438
H	-4.439184	-1.101811	-0.992417

Frequencies (cm^{-1}):	144.59	158.66	161.44	397.20	398.56
	526.76	526.86	559.50	571.12	620.96
	648.71	683.36	769.78	771.86	775.55
	1000.01	1058.10	1059.11	1252.45	1404.43
	1419.27	1420.91	1486.47	1487.08	1760.08
	1760.60	1822.58	3622.40	3626.74	3632.11

Table S11. Optimized cartesian coordinates (Å) of the 1a/1b pair at the PBE0/Def2-TZVP level used in the DFT validation.

1a

Atom	x	y	z
C	0.0062	-0.0079	-0.0001
C	1.4012	-0.0015	-0.0001
C	2.0298	1.2249	0.0000
C	1.2670	2.3888	0.0001
C	-0.1081	2.2595	0.0001
N	-0.7348	1.0839	0.0000
O	-0.6342	-1.1883	-0.0002
H	1.9470	-0.9358	-0.0002
H	3.1127	1.2777	0.0000
H	1.7281	3.3678	0.0002
H	-0.7479	3.1372	0.0002
H	-1.5779	-0.9817	-0.0002

1b

Atom	x	y	z
O	2.7539	0.3172	0.0000
C	1.5408	0.2074	0.0000
C	0.7805	-1.0205	0.0000
C	-0.5767	-1.0160	0.0000
C	-1.3135	0.1961	0.0000
C	-0.6177	1.3587	0.0000
N	0.7351	1.3502	0.0000
H	-2.3936	0.2048	0.0000
H	-1.0898	2.3332	0.0000
H	1.3533	-1.9385	0.0000
H	-1.1138	-1.9589	0.0000
H	1.2483	2.2192	0.0000

References

[1] K. Ruedenberg, *Rev. Mod. Phys.*, 1962, **34**, 326–376.

[2] R. P. Feynman, R. B. Leighton and M. Sands, *The Feynman lectures on physics: The Definitive Edition (Vol. 3)*, Pearson, 2009.

[3] T. M. Cardozo and M. A. C. Nascimento, *J. Chem. Phys.*, 2009, **130**, 104102.

[4] T. M. Cardozo, D. W. Oliveira De Sousa, F. Fantuzzi and M. A. Nascimento, *Compr. Comput. Chem.*, Elsevier, 2024, pp. 552–588.

[5] R. McWeeny, *Proc. R. Soc. London. Ser. A. Math. Phys. Sci.*, 1959, **253**, 242–259.

[6] W. Kutzelnigg, *Angewandte Chemie International Edition in English*, 1973, **12**, 546–562.
